# Molecular features of the serological IgG repertoire elicited by egg-based, cell-based, or recombinant haemagglutinin-based seasonal influenza vaccines: a comparative, prospective, observational cohort study

**DOI:** 10.1016/j.lanmic.2024.06.002

**Published:** 2024-12-09

**Authors:** Juyeon Park, Foteini Bartzoka, Troy von Beck, Zhu-Nan Li, Margarita Mishina, Luke S Hebert, Jessica Kain, Feng Liu, Suresh Sharma, Weiping Cao, Devon J Eddins, Amrita Kumar, Jin Eyun Kim, Justin S Lee, Yuanyuan Wang, Evan A Schwartz, Axel F Brilot, Ed Satterwhite, Dalton M Towers, Eric McKnight, Jan Pohl, Mark G Thompson, Manjusha Gaglani, Fatimah S Dawood, Allison L Naleway, James Stevens, Richard B Kennedy, Joshy Jacob, Jason J Lavinder, Min Z Levine, Shivaprakash Gangappa, Gregory C Ippolito, Suryaprakash Sambhara, George Georgiou

**Affiliations:** Department of Molecular Biosciences (J Park PhD, F Bartzoka PhD, L S Hebert BSc, J J Lavinder PhD, G C Ippolito PhD, Prof G Georgiou PhD), Department of Chemical Engineering (J Park, J Kain MSc, E Satterwhite PhD, D M Towers BSc, E McKnight BSc, J J Lavinder, Prof G Georgiou), and Department of Biomedical Engineering (J E Kim PhD, Prof G Georgiou), and Sauer Structural Biology Laboratory, Center for Biomedical Research Support (E A Schwartz PhD, A F Brilot PhD), The University of Texas at Austin, Austin, TX, USA; Department of Microbiology and Immunology, Emory Vaccine Center, School of Medicine, Emory University, Atlanta, GA, USA (T von Beck PhD, Prof J Jacob PhD); Centers for Disease Control and Prevention, Atlanta, GA, USA (Z-N Li PhD, M Mishina BSc, F Liu PhD, S Sharma PhD, W Cao PhD, D J Eddins PhD, A Kumar PhD, J S Lee PhD, Y Wang PhD, J Pohl PhD, M G Thompson PhD, F S Dawood MD, J Stevens PhD, M Z Levine PhD, S Gangappa PhD, S Sambhara DVM PhD); Baylor Scott & White Health, Baylor College of Medicine and Texas A&M University College of Medicine, Temple, TX, USA (Prof M Gaglani MBBS); Department of Medicine, Mayo Clinic, Rochester, MN, USA (R B Kennedy PhD); Kaiser Permanente Center for Health Research, Portland, OR, USA (A L Naleway PhD)

## Abstract

**Background:**

Egg-based inactivated quadrivalent seasonal influenza vaccine (eIIV4), cell culture-based inactivated quadrivalent seasonal influenza vaccine (ccIIV4), and recombinant haemagglutinin (HA)-based quadrivalent seasonal influenza vaccine (RIV4) have been licensed for use in the USA. In this study, we used antigen-specific serum proteomics analysis to assess how the molecular composition and qualities of the serological antibody repertoires differ after seasonal influenza immunisation by each of the three vaccines and how different vaccination platforms affect the HA binding affinity and breadth of the serum antibodies that comprise the polyclonal response.

**Methods:**

In this comparative, prospective, observational cohort study, we included female US health-care personnel (mean age 47·6 years [SD 8]) who received a single dose of RIV4, eIIV4, or ccIIV4 during the 2018–19 influenza season at Baylor Scott & White Health (Temple, TX, USA). Eligible individuals were selected based on comparable day 28 serum microneutralisation titres and similar vaccination history. Laboratory investigators were blinded to assignment until testing was completed. The preplanned exploratory endpoints were assessed by deconvoluting the serological repertoire specific to A/Singapore/INFIMH-16–0019/2016 (H3N2) HA before (day 0) and after (day 28) immunisation using bottom-up liquid chromatography–mass spectrometry proteomics (referred to as Ig-Seq) and natively paired variable heavy chain–variable light chain high-throughput B-cell receptor sequencing (referred to as BCR-Seq). Features of the antigen-specific serological repertoire at day 0 and day 28 for the three vaccine groups were compared. Antibodies identified with high confidence in sera were recombinantly expressed and characterised in depth to determine the binding affinity and breadth to time-ordered H3 HA proteins.

**Findings:**

During September and October of the 2018–19 influenza season, 15 individuals were recruited and assigned to receive RIV4 (n=5), eIIV4 (n=5), or ccIIV4 (n=5). For all three cohorts, the serum antibody repertoire was dominated by back-boosted antibody lineages (median 98% [95% CI 88–99]) that were present in the serum before vaccination. Although vaccine platform-dependent differences were not evident in the repertoire diversity, somatic hypermutation, or heavy chain complementarity determining region 3 biochemical features, antibodies boosted by RIV4 showed substantially higher binding affinity to the vaccine H3/HA (median half-maximal effective concentration [EC50] to A/Singapore/INFIMH-16–0019/2016 HA: 0·037 μg/mL [95% CI 0·012-0·12] for RIV4; 4·43 μg/mL [0·030–100·0] for eIIV4; and 18·50 μg/mL [0·99–100·0] μg/mL for ccIIV4) and also the HAs from contemporary H3N2 strains than did those elicited by eIIV4 or ccIIV4 (median EC50 to A/Texas/50/2012 HA: 0·037 μg/mL [0·017–0·32] for RIV4; 1·10 μg/mL [0·045–100] for eIIV4; and 12·6 μg/mL [1·8–100] for ccIIV4). Comparison of B-cell receptor sequencing repertoires on day 7 showed that eIIV4 increased the median frequency of canonical egg glycan-targeting B cells (0·20% [95% CI 0·067–0·37] for eIIV4; 0·058% [0·050–0·11] for RIV4; and 0·035% [0–0·062] for ccIIV4), whereas RIV4 vaccination decreased the median frequency of B-cell receptors displaying stereotypical features associated with membrane proximal anchor-targeting antibodies (0·062% [95% CI 0–0·084] for RIV4; 0·12% [0·066–0·16] for eIIV4; and 0·18% [0·016–0·20] for ccIIV4). In exploratory analysis, we characterised the structure of a highly abundant monoclonal antibody that binds to both group 1 and 2 HAs and recognises the HA trimer interface, despite its sequence resembling the stereotypical sequence motif found in membrane-proximal anchor binding antibodies.

**Interpretation:**

Although all three licensed seasonal influenza vaccines elicit serological antibody repertoires with indistinguishable features shaped by heavy imprinting, the RIV4 vaccine selectively boosts higher affinity monoclonal antibodies to contemporary strains and elicits greater serum binding potency and breadth, possibly as a consequence of the multivalent structural features of the HA immunogen in this vaccine formulation. Collectively, our findings show advantages of RIV4 vaccines and more generally highlight the benefits of multivalent HA immunogens in promoting higher affinity serum antibody responses.

**Funding:**

Centers for Disease Control and Prevention, National Institutes of Health, and Bill & Melinda Gates Foundation.

## Introduction

Seasonal influenza vaccination has been shown to attenuate the severity of symptomatic illness; however, the overall vaccine effectiveness of licensed influenza vaccines remains suboptimal, with only 35·7% average vaccine effectiveness in the past 10 years in the USA, according to the US Centers for Disease Control and Prevention. Among the influenza A viruses, the A/H3N2 strain is of particular importance due to its higher rate of antigenic drift than the A/H1N1 strain and associated lower vaccine effectiveness.^1^

Since the first report on growing influenza virus in embryonated chicken eggs in the 1930s, egg-produced viruses have been used to produce influenza vaccines. Drawbacks of egg-based inactivated quadrivalent seasonal influenza vaccine (eIIV4) include allergic responses to egg products and, separately, the need to introduce mutations around the haemagglutinin (HA) receptor binding site needed for propagation in chicken eggs and binding to avian receptors, namely α-2,3 linked sialic acids, as opposed to α-2,6 linked sialic acids in humans.^2^ Egg adaptation mutations in the eIIV4 vaccine can elicit antibodies that are unable to bind human influenza strains.^3,4^ More recently, we and others have shown that eIIV4 immunisation directs part of the antibody response towards avian antigens, such as sulfated type Galβ1–4GalNAcβ avian glycans, which are prevalent in egg allantoic fluid.^5,6^

To overcome these limitations, two alternate vaccine production platforms have been developed: inactivated subunit vaccines from virus grown in Madin–Darby Canine Kidney cells (cell culture-based inactivated quadrivalent seasonal influenza vaccine [ccIIV4]) and recombinant HA vaccines produced in insect cells (recombinant HA-based quadrivalent seasonal influenza vaccine [RIV4]), with ccIIV4 approved by the US Food and Drug Administration in November, 2012, and RIV4 in January, 2013. Some advantages of mammalian and insect cell tissue culture systems are their shorter vaccine production timelines and the lack of egg-adapted mutations and egg antigens. Similar to eIIV4, ccIIV4 vaccines use a standard dose of 15 μg HA per strain, whereas RIV4 vaccines are exclusively formulated with three times higher dose of HA (45 μg) per strain.

Although eIIV4, ccIIV4, and RIV4 have been in use for over 10 years, detailed comparative studies of vaccine effectiveness and serological responses are scarce. Izurieta and colleagues^7,8^ reported that ccIIV4 showed only minor improvement in relative vaccine effectiveness compared with eIIV4, and other studies found a similar titre of neutralising serum antibodies by ccIIV4 and eIIV4 immunisation against all four vaccine strains.^2,9^ Recently, Dawood and colleagues^9^ reported that RIV4 elicited higher neutralising antibody titres to A/H1N1, A/H3N2, and B/Yamagata strains than did standard-dose eIIV4 and, similarly, Wang and colleagues^2^ and Gouma and colleagues^10^ reported that RIV4 elicits broader H3N2 neutralisation breadth than either eIIV4 or ccIIV4. Importantly, a large clinical study of vaccine efficacy of more than 8000 adults aged 50 years or older (which provided key evidence used for licensure) revealed 30% higher protection against RT-PCR-confirmed influenza infections for RIV4 than for standard-dose eIIV4 during the H3N2-predominant 2014–15 season.^11^ Although it could be argued that the higher vaccine efficacy reported with RIV4 might be a consequence of the three times higher concentration of HA in this vaccine formulation (45 μg per strain) than in the standard-dose eIIV4 and ccIIV4 (15 μg per strain), this is not likely to be the case in light of the fact that with eIIV4, the Fluzone High-Dose Quadrivalent vaccine approved for the elderly (aged >65 years) has a four times higher HA dose (60 μg per strain), yet it does not result in higher neutralising antibody titres.^12^

To better understand how different vaccine platforms affect antibody clonal compositions and their respective quantities and qualities in the serum response, in this study we aimed to comprehensively profile the sequence identity, abundance, and binding affinity of H3/HA-specific circulating antibodies that comprise the polyclonal IgG serological repertoire in three vaccine cohorts who received either RIV4, eIIV4, or ccIIV4 during the 2018–19 influenza season.

## Methods

### Study design and participants

This comparative, prospective, observational cohort study is a preplanned exploratory analysis of the original randomised, open-label trial (NCT03722589)^9^ involving 727 US health-care workers. We selected 15 female (mean age 47·6 years [SD 8]) trial participants^9^ who received either RIV4 (Flublok Quadrivalent by Sanofi Pasteur, Swiftwater, PA, USA; 45 μg of HA per strain), eIIV4 (Fluzone Quadrivalent by Sanofi Pasteur; 15 μg of HA per strain), or ccIIV4 (Flucelvax Quadrivalent by Seqirus, Holly Springs, NC, USA; 15 μg of HA per strain; n=5 per cohort) during the 2018–19 influenza season at Baylor Scott & White Health, Temple, TX, USA. Participants were excluded if they had experienced any previous hypersensitivity to influenza vaccines or received any vaccination within 4 weeks before and after the initial visit.^9^ Eligible individuals were selected based on comparable day 28 serum microneutralisation titres and similar vaccination history. This strategy for selecting individuals allows for a direct comparison of molecular features in anti-H3/HA serum repertoires induced by different vaccine platforms while mitigating confounding effects that could arise from comparing individuals with large variations in post-vaccination titres. Another consideration in the inclusion of individuals was the availability of sufficient amounts of sera and peripheral blood mononuclear cells, as required for B-cell receptor sequencing (BCR-Seq) and immunoglobulin sequencing (Ig-Seq). 15 was the maximum number of individuals we could analyse, given sample availability and the cost of immunoglobulin sequencing experiments. Participants provided written informed consent before enrolment and trial participation. Baseline characteristics for all individuals were collected from electronic medical records. Investigators were blinded to vaccine groups until the completion of the study. Participants had serum (day 0 and day 28) and peripheral blood mononuclear cells (day 0 and day 7) collected before and after vaccination (appendix p 25).

### Procedures

Serum microneutralisation assays were performed using cell-grown A/Singapore/INFIMH-16–0019/2016 viruses propagated in MDCK-SIAT1 cells (MilliporeSigma, Burlington, MA, USA; appendix p 3). The microneutralisation titres were measured and reported by Dawood and colleagues^9^ as primary endpoints, which in turn guided donor selection in our preplanned exploratory analysis. Serum IgG binding titres were determined using ELISA to recombinant A/Singapore/INFIMH-16–0019/2016 HA (appendix p 3). Circulating T-follicular helper cells (CD4^+^CXCR5^+^PD1^+^CD25^−^) were identified by multiparametric fluorescence-activated cell sorting using fluorescent-labelled antibodies (appendix p 3).

**Figure 1:**
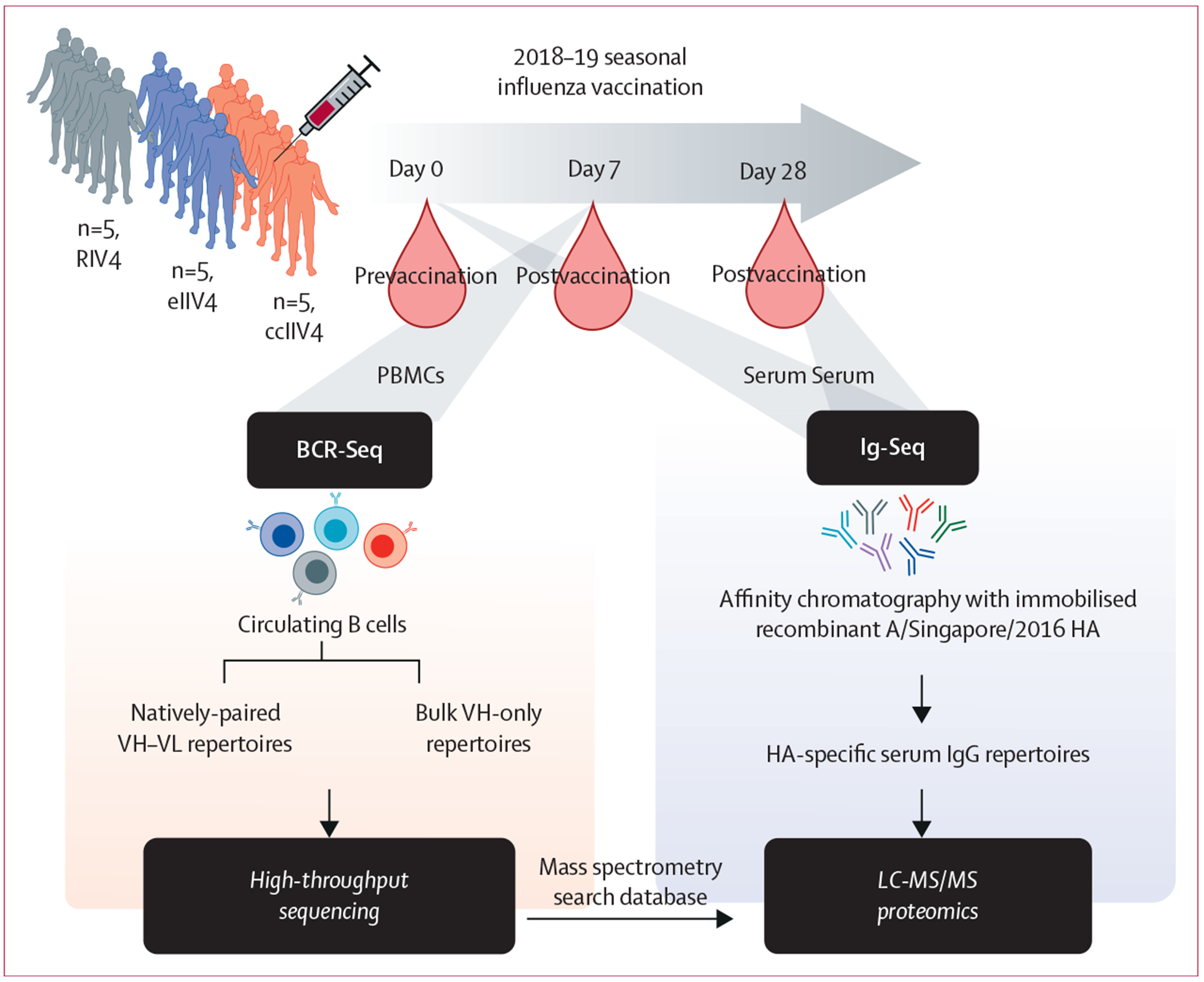
Strategy for proteomic profiling of serum IgG repertoires specific to A/Singapore/INFIMH-16–0019/2016 H3/HA BCR-Seq=B-cell receptor repertoire sequencing. ccIIV4=cell culture-based inactivated quadrivalent seasonal influenza vaccine. eIIV4=egg-based inactivated quadrivalent seasonal influenza vaccine. HA=haemagglutinin. Iq-Seq=high-resolution proteomics analysis of antigen-specific serum immunoglobulins repertoires. LC-MS/MS=liquid chromatography–tandem mass spectrometry. MS=mass spectrometry. PBMC=peripheral blood mononuclear cells. RIV4=recombinant haemagglutinin-based quadrivalent seasonal influenza vaccine.

We used the serum proteomics workflow, Ig-Seq, which capitalises on liquid chromatography–tandem mass spectrometry (LC-MS/MS)-based serum proteomics combined with subject-specific, natively paired sequencing of variable heavy chains (VH)–variable light chains (VL) in peripheral B cells that provides a database for mass spectra interpretation and full-length antibody sequences, which can in turn be recombinantly produced for biochemical and functional characterisation (figure 1). The VH-only or VH–VL paired high-throughput BCR-Seq was performed using bulk and single-cell day 7 circulating B-cell sequencing, respectively, as previously described (appendix pp 4–6).^13^ HA-binding antibodies were isolated from IgG plasma by affinity chromatography with immobilised A/Singapore/INFIMH-16–0019/2016 HA and analysed by LC-MS/MS, as described previously (appendix pp 6–7).^5,14,15^ The mass spectrometry search identified peptide spectra matches originating from heavy-chain complementarity determining region 3 (CDRH3) sequences, and the abundance of each clonotype was calculated by summing the extracted ion chromatogram (XIC) peak area of CDRH3 peptides mapped to a given clonotype (appendix pp 7–9). We selected monoclonal antibodies for which high-confidence CDRH3 peptides in serum were identified by LC-MS/MS at high abundance (as determined by XIC area), along with high peptide coverage of the VH, especially for the complementarity determining regions (appendix pp 26–27). The binding affinity of recombinant monoclonal antibodies was determined by ELISA against A/Texas/50/2012 and A/Singapore/INFIMH-16–0019/2016 HA (appendix pp 9–10). The high-throughput multiplex influenza antibody detection assay was conducted using multiplexed microsphere beads containing a broad panel of H3/HAs and nucleoprotein (appendix pp 4, 28). The binding kinetics of UT14 and its competition with known monoclonal antibodies were determined using biolayer interferometry (appendix pp 10–11). Cryo-electron microscopy structure of UT14 Fab in complex with A/Singapore/INFIMH-16–0019/2016 HA was determined using FEI Titan Krios G3 300kV cryo-EM (Thermo Fisher Scientific, Waltham, MA, USA) with a K3 direct detection camera (appendix pp 11, 31). Full details on sources and identifiers of reagents used in this study are in the appendix (pp 3–11).

### Outcomes

The primary exploratory outcome of this study was to compare the molecular composition of the HA-specific IgG antibody repertoire after vaccination by RIV4, eIIV4, or ccIIV4. As key secondary outcomes, the level of back-boosting, molecular features of serum clonotypes, binding affinity of representative monoclonal antibodies, HA serum-binding landscape against time-ordered H3 HA variants, correlation of antibody repertoire features with circulating T-follicular helper cell frequencies, and stereotypical B-cell receptor responses were evaluated. An additional exploratory outcome involved analysing the biochemical and structural features of an unusual near-stereotypical monoclonal antibody, which was detected at a high abundance in serum.

### Statistical analysis

For multiple comparisons, ordinary one-way ANOVA tests followed by Tukey’s post-hoc tests, Welch’s ANOVA tests followed by Dunnett’s T3 post-hoc tests, or Kruskal–Wallis tests followed by Dunn’s post-hoc tests were used based on the assessment of normality and homogeneity of variance assumptions (appendix p 11). Unpaired or paired comparisons between two groups were conducted using the two-sided Mann–Whitney U or Wilcoxon matched-pairs signed rank tests, respectively. The Pearson correlation tests were conducted using Scipy python package version 1.9.1. The Tukey-style box-and-whisker plot was drawn using default geom_boxplot function by ggplot2 version 3.4.2. All raw data points are shown in the box-and-whisker or violin plot. Data are presented as median with 95% CI estimates or mean (SD). Statistical analyses were conducted using GraphPad Prism version 10.2.1 using a threshold for significance of p<0·05.

### Role of the funding source

The funders of the study had no role in study design, data collection, data analysis, data interpretation, or writing of the report.

## Results

**Figure 2:**
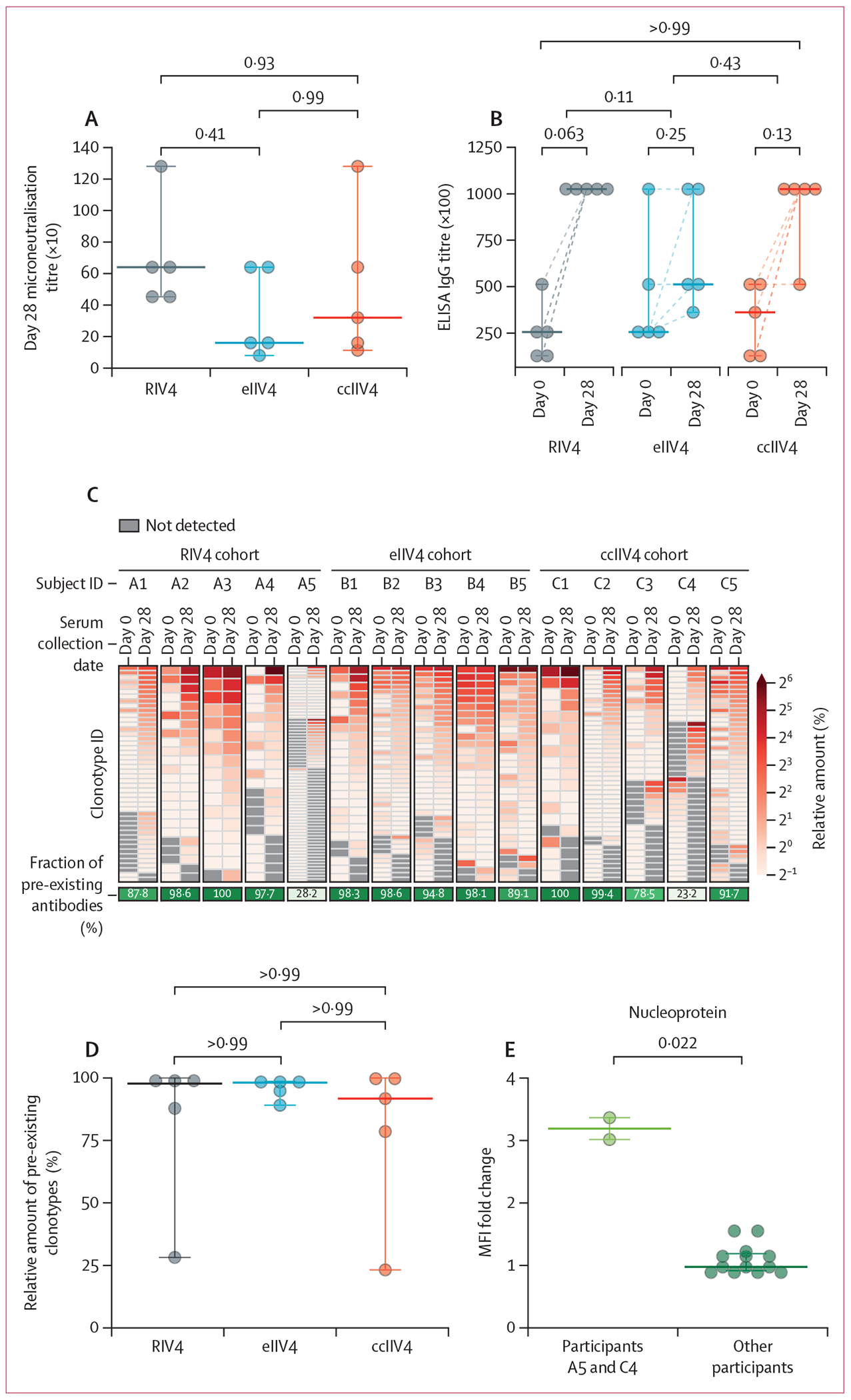
Proteomic analysis of serum IgG repertoires elicited by RIV4, standard-dose eIIV4, and ccIIV4 2018–19 quadrivalent vaccination (A) Day 28 serum microneutralisation titres against cell-grown A/Singapore/INFIMH-16–0019/2016 H3 vaccine viruses. (B) Serum ELISA binding titres to recombinant A/Singapore/INFIMH-16–0019/2016 H3/HA on day 0 or day 28 for each vaccine cohort. The dotted line between the two timepoints connects the same individual. Statistical analysis was performed using the two-tailed Wilcoxon matched-pairs signed rank test. (C) Clonal composition and relative abundance of serum IgG repertoires before and after immunisation. Each row in the repertoire heatmap represents a unique clonotype ID detected at day 0 or day 28 for each individual. The heatmap includes all clonotypes detected at >0·5% of the total amount (integrated extracted ion chromatogram peak areas). (D) Serum abundance of pre-existing clonotypes in the post-vaccination repertoire (the green box in panel C) was compared between different vaccine cohorts. (E) Fold change in MFI to nucleoprotein at day 28 relative to day 0 for the two atypical individuals (ID A5 and C4) and typical individuals (n=13). Statistical analysis was done using the two-tailed Mann–Whitney test. The horizontal line indicates the median, and the error bar indicates the 95% CI (A, B, D, and E). Each point represents an individual (A, B, D, and E). For multiple comparisons across the three vaccine cohorts, Kruskal–Wallis tests followed by Dunn’s post-hoc tests were performed (A, B, and D). ccIIV4=cell culture-based inactivated quadrivalent seasonal influenza vaccine. eIIV4=egg-based inactivated quadrivalent seasonal influenza vaccine. HA=haemagglutinin. MFI=mean fluorescence intensity. RIV4=recombinant haemagglutinin-based quadrivalent seasonal influenza vaccine.

We selected 15 female health-care personnel (n=5 per vaccine cohort) who were enrolled in a large clinical trial^9^ for cohorts RIV4 (participants A1–5; mean age 47·8 years [SD 4·7]), eIIV4 (participants B1–5; 46·8 years [9·5]), and ccIIV4 (participants C1–5; 48·2 years [11·3]). Participants received a single dose of RIV4, standard-dose eIIV4, or ccIIV4 during the 2018 September-to-October period of the 2018–19 influenza season (table).^9^ We chose our study cohorts to have statistically similar serum micro-neutralisation and ELISA binding titres to A/Singapore/INFIMH-16–0019/2016 (H3N2) virus on day 28 after vaccination (figure 2A, B). Serum IgG ELISA binding titres significantly correlated with the serum microneutralisation titres to the vaccine H3 strain (p=0·0001, Pearson *r*=0·64 [95% CI 0·21-0·86]) .

Ig-Seq serum proteomics analysis of A/Singapore/INFIMH-16–0019/2016 H3/HA affinity-purified IgGs showed that all three vaccines elicited a highly polarised serological repertoire, dominated by back-boosted antibodies that were also detectable at day 0 (median percentage preexisting: 98% [95% CI 23–100] for RIV4, 98% [89–99] for eIIV4, 92% [23–100] for ccIIV4; p=1·0 for all multiple comparisons; figure 2C, appendix pp 12–13). The serological repertoires comprised a few highly abundant clonotypes, with the top three most abundant clonotypes accounting for a median 58% (95% CI 46–69) of the post-vaccination repertoire by abundance. The back-boosted (pre-existing) antibodies constituted a median 98% (95% CI 88–99) of the anti-H3/HA serum response, with no significant differences observed among the three vaccine cohorts (figure 2D). Interestingly, two individuals (participant identifiers A5 in the RIV4 cohort and C4 in the ccIIV4 cohort) had an unexpectedly 3·8-times lower fraction of pre-existing antibodies at day 28 (28% and 23% by abundance, respectively) compared with the other 13 individuals who had a median 98% (95% CI 89–99) of back-boosted antibodies in their sera. We noticed that these two individuals, compared with the rest of the cohort, had a significantly higher increase in serum nucleoprotein (NP) titre by a median 3·2 times (95% CI 3·0–3·4) on day 28 comparedwith day 0, whereas the other individuals did not have appreciable changes in NP titre between the two timepoints (differences in NP titre change, p=0·02; figure 2E; appendix p 14). This finding suggests that the two donors with atypical fractions of pre-existing antibodies and NP titres might have had subdinical influenza infection around the time of vaccination, given that they received recombinant HA-based and inactivated subunit vaccines.

**Figure 3:**
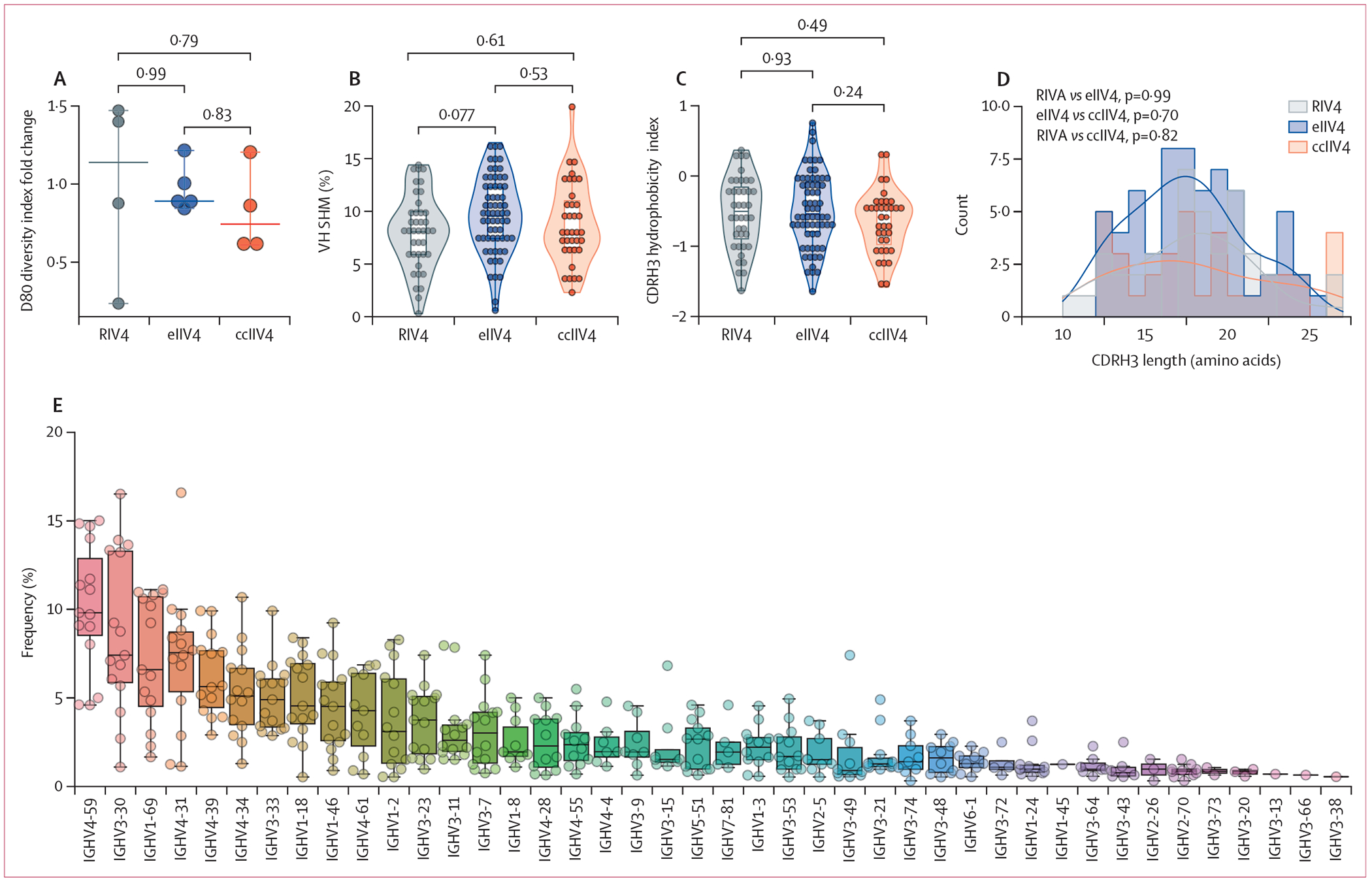
Comparative analysis of molecular features in the serological IgG repertoire to A/Singapore/INFIMH-16–0019/2016 (H3N2) HA (A) Fold change in the D80 diversity index of the anti-H3/HA serological repertoire at day 28 compared with day 0 (n=13 typical donors). Each dot represents each individual.The horizontal line and error bar represent the median and 95% CI. Comparison of percentage VH somatic hypermutation (B), CDRH3 hydrophobicity index (GRAVY index; C), and CDRH3 amino acid length (D) for day 28 serum antibody clonotypes specific to antigen. The violin and box-whisker plot are overlaid (B and C). The smooth distribution curve was calculated using kernel density estimation and was superimposed on the histogram (D). (E) Frequency of VH genes used in the anti-H3/HA serum repertoire after vaccination. Colours indicate different VH genes. Each dot represents all HA-specific day 28 IgG clonotypes (B, C, and E). Statistical analysis was performed using ordinary one-way ANOVA followed by Tukey’s multiple comparison tests (A–D). ccIIV4=cell culture-based inactivated quadrivalent seasonal influenza vaccine. CDRH3=complementarity determining region 3. eIIV4=egg-based inactivated quadrivalent seasonal influenza vaccine. HA=haemagglutinin. RIV4=recombinant haemagglutinin-based quadrivalent seasonal influenza vaccine. VH=variable heavy chain.

We analysed the molecular features of the serological IgG repertoire associated with different vaccine platforms, specifically among the 13 donors who showed no sign of infection. For these individuals, the clonal composition and the extent of repertoire polarisation on day 28 versus day 0 were not influenced by the type of vaccination received, as measured by D80 diversity index (p>0·05; figure 3A). Furthermore, the IgG antibodies comprising the anti-H3/HA serum response had the same molecular features in terms of VH somatic hypermutation, CDRH3 hydrophobicity, and the CDRH3 amino acid length across all three vaccine cohorts (p>0·05; figure 3B–D). In the case of the typical donors, pre-existing antibodies had a higher level of VH somatic hypermutation compared with newly elicited antibodies (p=0·030; appendix p 15) for all vaccine cohorts.

Given the indistinguishable features of the anti-H3/HA serum repertoires, we examined whether molecular characteristics of the day 28 repertoire correlate with immunological parameters regardless of the vaccine received. Across all 15 individuals, serum antibodies encoded by IGHV4–59, IGHV3–30, IGHV1–69, IGHV4–31, and IGHV4–39 were used with higher frequency than other VH gene families (figure 3E). IGHV1–2, IGHV5–51, and IGHV1–18 showed higher serum abundance when calculated by LC-MS/MS XIC peak area, albeit less frequently (appendix p 16). Additionally, we found that a reduction in the repertoire diversity (in other words, an increase in polarisation) correlated with an increase in circulating follicular helper T-cell frequency at day 7 versus day 0, although this observation did not reach statistical significance (p=0·068; appendix p 17).

**Figure 4:**
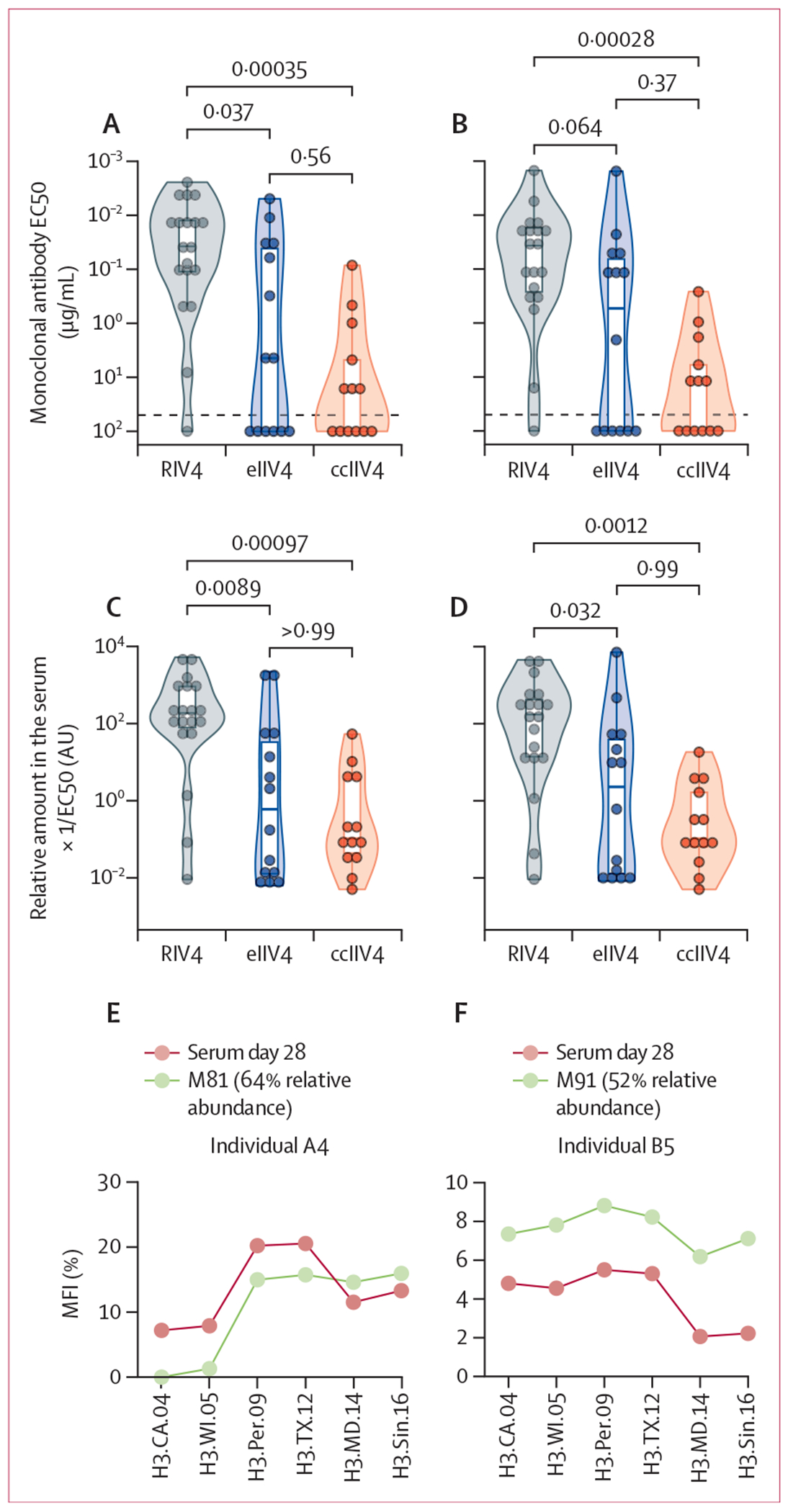

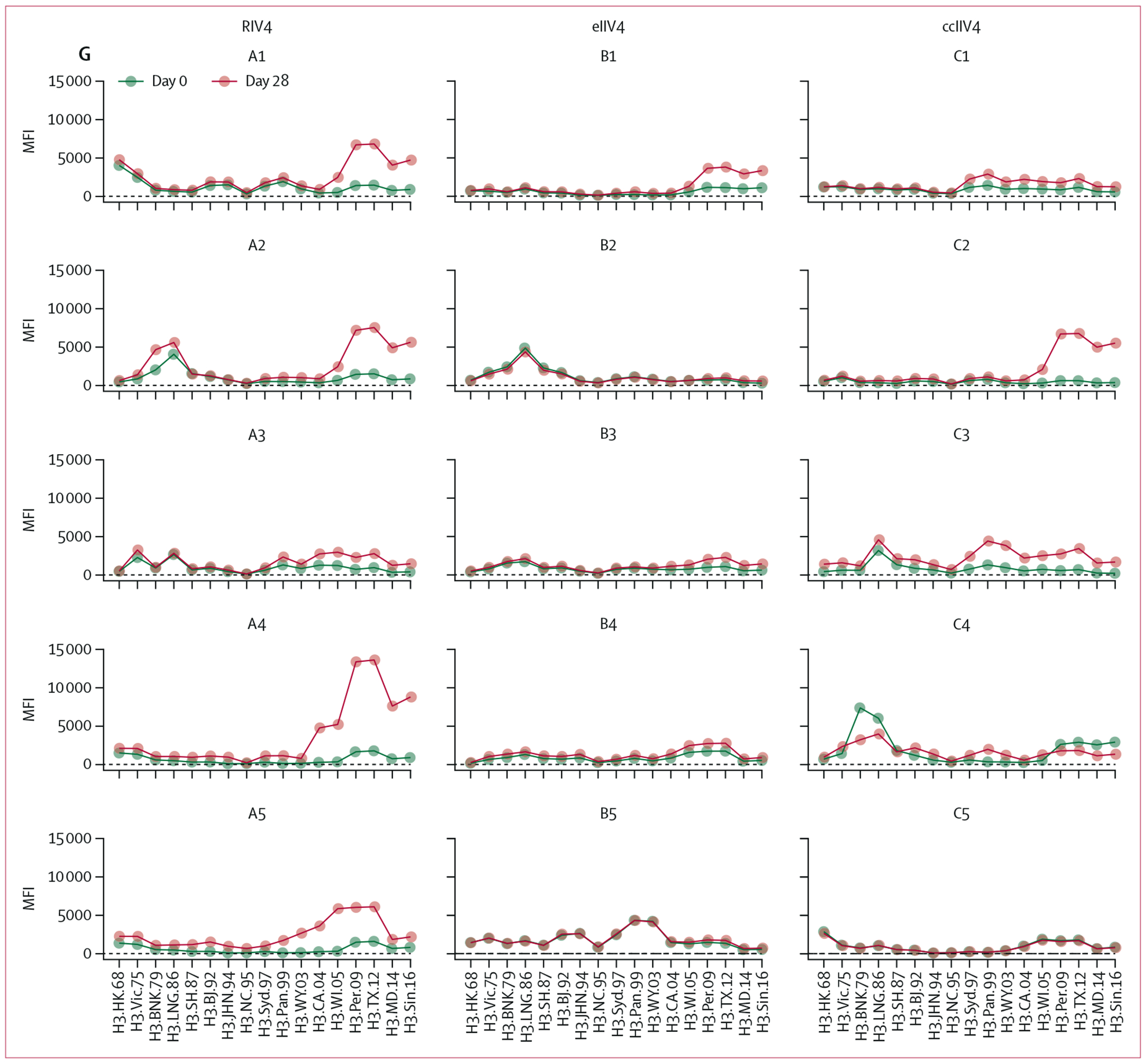
Different qualities of monoclonal antibodies elicited by distinct vaccine platforms The binding affinity to recombinant A/Singapore/INFIMH-16–0019/2016 (A) or A/Texas/50/2012 (B) H3/HAs for representative monoclonal antibodies identified at high abundance in serum was compared in the three vaccine cohorts. The relative abundance of monoclonal antibodies in the serum normalised by their binding potency (1/EC50) to either A/Singapore/INFIMH-16–0019/2016 (C) or A/Texas/50/2012 (D) HAs was compared across different vaccine cohorts. Each dot represents monoclonal antibodies selected from an individual immunised with either RIV4, eIIV4, or ccIIV4 (A–D). Comparison of the binding landscape of top-most abundant monoclonal antibodies with the binding landscape of complete serum in individuals A4 (E) and B5 (F). (G) The complete serum binding landscape to H3/HAs before and after vaccination for each individual immunised with RIV4, eIIV4, or ccIIV4. MFI was measured using monoclonal antibodies (E and F) or serum on day 0 or day 28 (G) via multiplexed Luminex assay. The line plot connects the mean fluorescence intensity across different HA antigens (G). The dotted lines indicate the limit of quantification (A, B, and G). Statistical analysis was performed using Kruskal–Wallis tests followed by Dunn’s post-hoc tests (A–D). AU=arbitrary unit. ccIIV4=cell culture-based inactivated quadrivalent seasonal influenza vaccine. EC50=half-maximal effective concentration. eIIV4=egg-based inactivated quadrivalent seasonal influenza vaccine. HA=haemagglutinin. MFI=Mean fluorescence intensity. RIV4=recombinant haemagglutinin-based quadrivalent seasonal influenza vaccine.

To compare the quality of monoclonal antibodies identified in the serum repertoire among different vaccine cohorts, we recombinantly expressed antibodies representative of dominant serum clonotypes (appendix p 18). Although monoclonal antibodies from all three vaccine cohorts had similar levels of VH somatic hypermutation (p>0·05; appendix p 18), we found that the monoclonal antibodies induced by RIV4 had a substantially higher affinity to the current vaccine A/Singapore/INFIMH-16–0019/2016 HA and A/Texas/50/2012 HA used in the preceding 2014–15 season than did those induced by the other two vaccines (figure 4A–B, appendix p 18). For the RIV4 cohort, the median half-maximal effective concentration (EC50) of monoclonal antibodies was 0·037 μg/mL (95% CI 0·012–0·12) and 0·037 μg/mL (0·017–0·32) for A/Singapore/INFIMH-16–0019/2016 and A/Texas/50/2012 HAs, respectively, which is approximately two orders of magnitude (30 to 500 times) lower than the median EC50 of monoclonal antibodies induced by either eIIV4 or ccIIV4 (H3 Singapore, 4·43 μg/mL [95% CI 0·030–100] for eIIV4, 18·50 μg/mL [0·99–100] for ccIIV4; H3 Texas, 1·10 μg/mL [0·045–100] for eIIV4, and 12·63 μg/mL [1·83–100] for ccIIV4; figure 4A–B). Notably, we found that higher affinity monoclonal antibodies boosted by RIV4 contributed a significantly larger fraction of the serum response than those elicited by eIIV4 or ccIIV4 (figure 4C–D). There was no significant difference in the quality of monoclonal antibodies constituting the serum response in the eIIV4 and ccIIV4 cohort (figure 4A–D).

We analysed the binding landscapes of bulk serum and also of top-dominant monoclonal antibodies (ie, detected at high concentrations in the serum) against a time-ordered panel of H3/HAs via multiplexed Luminex assay.^16^ In two individuals (A4 and B5) for whom we detected dominant clonotypes that accounted for more than 50% of the anti-H3/HA serum response, the binding landscape for these two dominant clonotypes, M81 and M91, closely mirrored the binding landscape observed with whole serum. The concordance in the binding pattern of sera and the dominant antibodies identified by Ig-Seq suggest that a single antibody lineage can largely dictate the functional properties of the polyclonal serum response (figure 4E–F). Deep scanning saturation mutagenesis could, in principle, further assist in delineating the role of non-dominant serum antibodies in shaping binding breadth and possibly viral escape.^17^ Additionally, consistent with the larger fraction of high affinity monoclonal antibodies boosted by RIV4, complete sera from RIV4 vaccine recipients had a substantially higher and broader increase in H3/HA binding response to contemporary strains that have been circulating after the year 2000 (figure 4G; appendix pp 19, 29), compared with eIIV4 and ccIIV4 recipients.

Given that we generated a very large set (> 10^6^) of antibody VH–VL paired sequences from day 7 total B cells, a population in which antigen-specific plasmablasts are highly enriched after influenza vaccination,^18^ we examined B-cell receptor clonotypes with known stereotypical HA binding sequence features within this dataset (appendix pp 20, 30). Although no significant differences were detected in the stereotypical B-cell responses targeting the central stalk, trimer interface, and group 1 and group 2 broadly neutralising stem epitopes, eIIV4 elicited a significantly higher frequency of canonical egg-glycan binding antibodies than did ccIIV4 (median 0·196% [95% CI 0·067–0·372] for eIIV4, 0·035% [0·000–0·062] for ccIIV4, p=0·0071; appendix p 20). Furthermore, stereotyped B-cell receptors associated with binding to membrane-proximal anchor epitope were three times less frequent in the RIV4 cohort than in the ccIIV4 cohort, although the difference was not statistically significant (median 0·062% [95% CI 0·000–0·084] for RIV4, 0·181% [0·016–0·195] for ccIIV4, p=0·064; appendix p 20). Interestingly, even though we detected multiple antibodies with stereotypical HA binding features in day 7 peripheral B cells, only two near stereotypical monoclonal antibodies, UT14 and M47 (a stereotypical trimer interface monoclonal antibody), were detected in the serum of two of the 15 individuals analysed.

**Figure 5:**
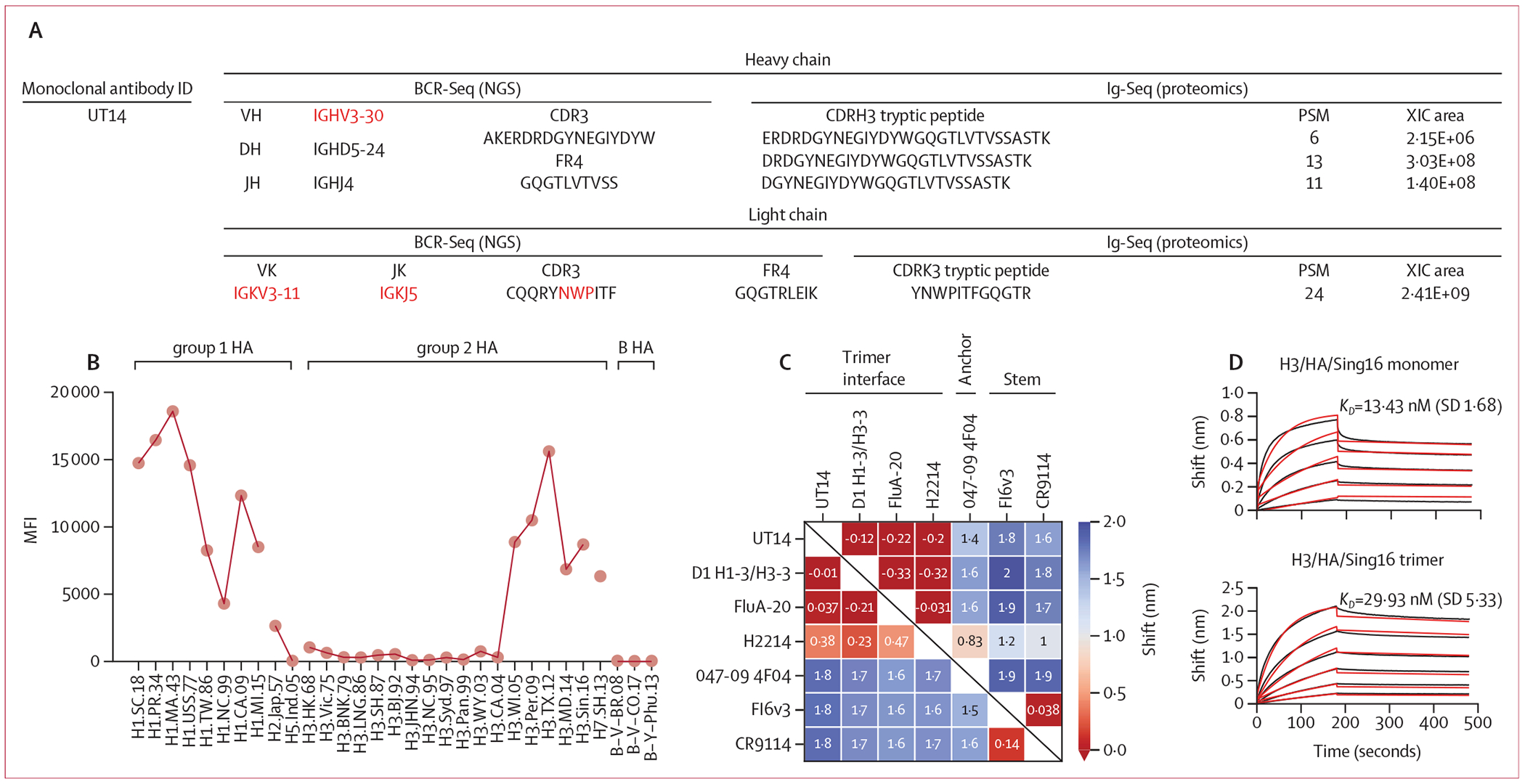
Molecular, biochemical, and structural characterisation of UT14 elicited by ccIIV4 (A) Stereotypical sequence features matching known membrane-proximal anchor antibodies (red) for UT14. CDRH3 and CDRK3 tryptic peptides were detected in the eluate of H3 HA affinity chromatography and absent in the flow-through on day 28, consistent with the presence of UT14 in serum and its binding to H3/HA. Amino acid sequences were defined as follows: AKERDRDGYNEGIYDYW=AlaLysGluArgAspArgAspGlyTyrAsnGluGlyIleTyrAspTyrTrp; GQGTLVTVSS=GlyGlnGlyThrLeuValThrValSerSer; CQQRYNWPITF=CysGlnGlnArgTyrAsnTrpProIleThrPhe; GQGTRLEIK=GlyGlnGlyThrArgLeuGluIleLys; ERDRDGYNEGIYDYWGQGTLVTVSSASTK=GluArgAspArgAspGlyTyrAsnGluGlyIleTyrAspTyrTrpGlyGlnGlyThrLeu.ValThrValSerSerAlaSerThrLys; DRDGYNEGIYDYWGQGTLVTVSSASTK=AspArgAspGlyTyrAsnGluGlyIleTyrAspTyrTrpGlyGlnGlyThrLeuValThr;ValSerSerAlaSerThrLys; DGYNEGIYDYWGQGTLVTVSSASTK=AspGlyTyrAsnGluGlyIleTyrAspTyrTrpGlyGlnGlyThrLeuValThrValSerSerAlaSerThrLys; and YNWPITFGQGTR=TyrAsnTrpProIleThrPheGlyGlnGlyThrArg. (B) Binding breadth of UT14 tested by multiplexed Luminex assay against A/group 1, A/group 2, and B/HAs. The dotted line indicates the limit of quantification. (C) Biolayer interferometry competition of UT14 with known trimer interface (D1 H1–3/H3–3, FluA-20, and H2214), membrane-proximal anchor region (047–09 4F04), or central stalk region (FI6v3 and CR9114) monoclonal antibodies for binding to A/California/07/2009 H1 HA. (D) Biolayer interferometry binding kinetics of UT14 Fab to monomer (top) and trimeric (bottom) forms of A/Singapore/INFIMH-16–0019/2016 H3/HA. Raw sensorgram data, black. Curve fit, red. Mean (SD) of the dissociation constant (*K_D_*) was shown. BCR-Seq=B-cell receptor repertoire sequencing. ccIIV4=cell culture-based inactivated quadrivalent seasonal influenza vaccine. CDRH3=complementarity determining region 3 of the heavy chain. CDRK3=complementarity determining region 3 of the kappa light chain. HA=haemagglutinin. Ig-Seq=bottom-up liquid chromatography–tandem mass spectrometry proteomics analysis of antigen-specific serum immunoglobulins. MFI=mean fluorescence intensity. NGS=next-generation sequencing. PSM=peptide spectra match. XIC=extracted ion chromatogram.

UT14 is a heterosubtypic monoclonal antibody that was found to be abundant in the serum of a ccIIV4 recipient and that possessed conserved anchor binding sequence features, such as the use of VH3–30, IGKV3–11, and IGKJ5 genes, along with Asn-Trp-Pro amino acid motif in the CDR3 of the kappa light chain (CDRK3; figure 5A, B).^19^ However, UT14 has a nine amino acid-long CDRK3, as opposed to a ten amino acid-long CDRK3 seen in other antibodies of this class (appendix p 21).^19^ In addition to detecting the UT14 CDRH3 peptide that defines the lineage in the serum, we also detected by LC-MS/MS unique tryptic peptides that contain the Asn-Trp-Pro region from the CDRK3 region, further confirming its serological relevance (figure 5A). Biolayer interferometry competition assays revealed that UT14 does not compete with anchor nor stem monoclonal antibodies; however, it competes with known trimer interface monoclonal antibodies (figure 5C; appendix p 21).^14,20–22^ The higher binding affinity of UT14 Fab towards monomeric HA than trimeric HA indicates that the UT14 epitope could be less readily accessible in trimeric HA (figure 5D).

**Figure 6:**
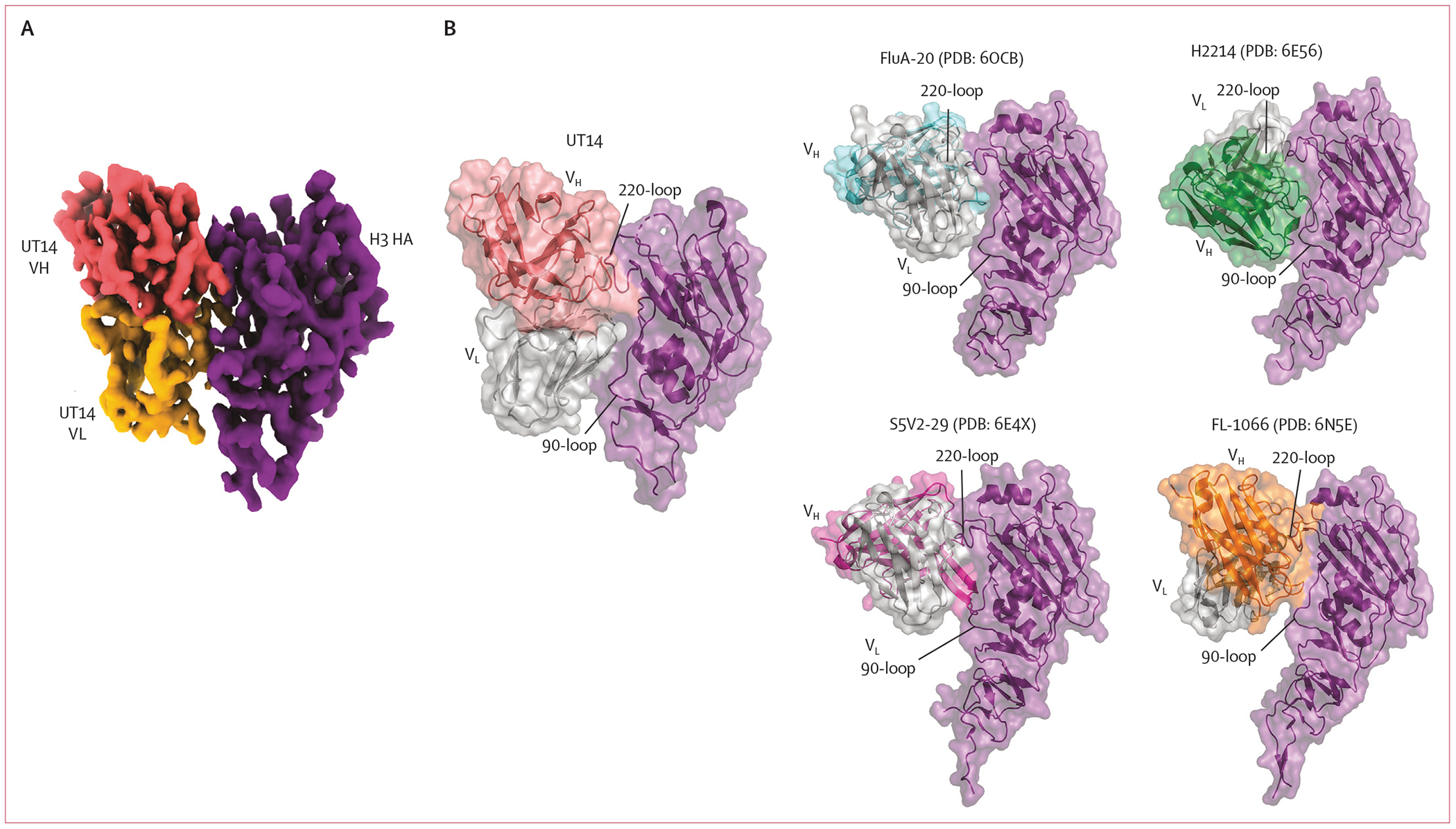
Cryo-EM structural analysis of UT14 binding to the trimer interface epitope of H3 HA head (A) Cryo-electron microscopy three dimensional reconstruction of a side view of UT14 variable region bound to A/Singapore/INFIMH-16–0019/2016 H3/HA head. (B) Comparison of the angles of approach among UT14, FluA-20 (PDB identifier: 6OCB), H2214 (PDB identifier: 6E56), S5V2–29 (PDB identifier: 6E4X), and FL-1066 (PDB identifier: 6N5E) monoclonal antibodies in complex with H3/HA heads (purple). HA regions with poor or missing electron microscopy density were excluded from the UT14-H3 model. PDB=Protein Data Bank. VH=variable heavy chain. VL=variable light chain.

We further obtained a 3·8Å resolution cryo-electron microscopy structure of UT14 with HA, revealing that this antibody buries 808Å^2^ lateral surface on the H3 head at the interface between two H3 protomers of the trimer (figure 6A; appendix pp 22–24). Similar to the human monoclonal antibodies, FluA-20,^21^ H2214,^22^ and S5V2–29,^22^ and the murine monoclonal antibody, FL-1066,^23^ the UT14 Fab interacts with the HA 220-loop and 90-loop via both its heavy (220 loop) and light chains (220 and 90 loop) while demonstrating a distinct angle of approach relative to the aforementioned trimer interface antibodies (figure 6B). UT14 utilises its light chain extensively for HA recognition, a feature that distinguishes UT14 and the murine FL-1066 from the human FluA-20, H2214, and S5V2–29-monoclonal antibodies, and faces the HA in a similar orientation with FL-1066. Unlike the Asn-Trp-Pro motif critical for binding to the anchor epitope,^19^ P95 in the Asn-Trp-Pro residues of UT14 CDRK3 does not interact with the HA. Instead, CDRK3 residues, including R91 and Y92, along with the N93 and W94, engage in epitope contact (appendix p 24).

## Discussion

Our findings suggest that for all three influenza A vaccines—RIV4, eIIV4, and ccIIV4—the serological repertoire was heavily shaped by back-boosting, with more than 80% of the antigen-specific clonal lineages found in serum having been elicited by previous exposures and thus detected at day 0. Due to this high degree of serological imprinting, vaccination with RIV4, eIIV4, or ccIIV4 results in serological anti-H3/HA clonotypes having similar repertoire diversity, VH somatic hypermutation, and CDRH3 features. These results are in line with the fact that A/Singapore/INFIMH-16–0019/2016 is antigenically similar to A/Hong Kong/4801/2014, which had been used as H3 vaccine strains in the two preceding 2016–17 and 2017–18 seasons, and that all individuals had extensive influenza vaccination records in the past 5 consecutive years. Furthermore, irrespective of the vaccine received, our findings support the notion that the serological response to H3/HA is driven by antibodies derived from a small set of select VH gene families.^14,24^ The trend in correlation between circulating T-follicular helper cell responses and the degree of polarisation suggests that highly polarised serum response, likely to be derived from a few dominant expanded B cells, might require more robust help from circulating T-follicular helper cells.

Importantly, from a clinical standpoint, we present data showing that the RIV4 vaccine preferentially boosts H3/HA-specific clonotypes that have much higher affinity for the vaccine HA as well as greater binding breadth to contemporary H3N2 strains than do eIIV4 and ccIIV4. Since we saw no statistical difference in the amount of HA-specific IgG that could be isolated from sera by affinity purification with immobilised H3/HA, we conclude that it is the preferential back-boosting of high-affinity monoclonal antibodies by RIV4 that is probably responsible for the greater increase in H3 serum landscape observed with RIV4 vaccination.

Clinical serological testing for eIIV4, ccIIV4, and RIV4^2,7–12^ cannot address the question of how antigenic and structural differences among the three licensed vaccines affect the composition and functional features of the serological repertoire at the molecular level. One recent study analysing plasmablast-encoded monoclonal antibodies reported that RIV4 elicited a broader homo-subtypic breadth relative to ccIIV4.^25^ However, the singlecell cloning from peripheral B cells, although immensely valuable, does not provide information about the antigen-specific serological repertoire that constitutes the polyclonal serum response for multiple reasons, including the fact that less than 5% of plasmablast-encoded antibodies are detectable in circulation and thus have a role in protection against viral infection.^15^

Extensive earlier studies and more recent mathematical modelling of the germinal centre reaction indicate a non-linear correlation between antigen dosage and antibody affinity, in which an optimally moderate dose, one that is not too high or too low, can lead to the production of high-affinity antibodies.^26,31^ A recent structural study revealed that the RIV4 vaccine exclusively contains starfish-like HA multivalent structures consisting of five to 12 copies of HA trimers clustered together to form a transmembrane core.^27^ By contrast, the ccIIV4 and eIIV4 vaccines contain comparable or higher fractions of individually isolated HA trimers than HA multimers.^27^ Given that the multivalent presentation of immunogens has been shown to enhance antibody responses and increase the affinity of bound monoclonal antibodies,^28^ we speculate that RIV4 immunogen structure probably affects the binding affinity of RIV4-boosted monoclonal antibodies observed in our study.

Furthermore, the crowding of HA stem domains within the starfish-like HA structures of RIV4 was estimated to occlude about 28% of stem epitopes due to steric clashes.^27^ This structural feature of the RIV4 immunogen might affect the ability to activate B cells binding to less accessible epitopes in the HA stem region, which is consistent with our finding of a decrease in the frequency of day 7 B-cell clonotypes with membrane-proximal anchor selective stereotypical features. By analogy, stabilised HIV-1 envelop antigens displayed on nanoparticles reduced the accessibility of epitopes proximal to the base of the antigen due to steric crowding with neighboring antigens on the nanoparticle surface.^29^ Multivalent display of antigens has been further shown to enhance trafficking to follicular dendritic cells and accumulation in germinal centres.^30^

Lastly, we report on UT14, a highly abundant monoclonal antibody, that has nearly all stereotypical features of membrane-proximal anchor antibodies reported by Guthmiller and colleagues,^19^ although it differs by having a nine amino acid-long CDRK3 instead of a canonical ten amino acid-long CDRK3.^19^ A closer inspection revealed that one more conserved proline adjacent to Asn-Trp-Pro (ie, Asn-Trp-Pro-Pro) is likely an essential feature of the anchor stereotype (Guthmiller JJ, personal communication). This additional Pro amino acid is 100% conserved in all anchor-binding antibodies and is likely critical for stabilising the Asn-Trp-Pro loop for epitope binding.^19^ Our findings thus highlight how antibodies with highly similar sequence features might have evolved to recognise distinct epitopes on the same HA antigen. The discovery of diverse heavy and light chain gene rearrangements in antibodies targeting the recurrent HA epitopes will continue refining our understanding of canonical and non-canonical antibody responses.

There are several limitations in our study, including the small sample size (n=5 individuals per cohort) and the fact that the cohorts comprise female health-care personnel (mean age 47·6 years [SD8]) and thus are not representative of diverse populations with different baseline characteristics, such as age, sex, ethnicity, or health status. Additionally, the anti-H1/HA serological repertoire could not be analysed due to limitations in the amount of serum and peripheral blood mononuclear cells that had been obtained under the institutional review board protocol. Further studies are needed to determine how the vaccine platform-specific repertoires are shaped in a diverse cohort of individuals and for different HA subtypes. Nevertheless, our finding of back-boosted antibodies dominant in all three vaccine cohorts, along with the prevalence of high-affinity monoclonal antibodies boosted by RIV4, points to strategies for designing more efficacious vaccines.

## Supplementary Material

Supplementary Material

## Figures and Tables

**Table:** Baseline characteristics for all individuals

	Individuals (n=15)
Age, years	49 (42–54)

Female	15 (100%)

White	13 (87%)

Hispanic	4 (27%)

BMI, kg/m^2^	30 (29–33)

Subjective health status*	2 (2–3)

Chronic medical condition	2 (13%)

Smoking status	
Every day	2 (13%)
Some days	1 (7%)
Not at all	12 (80%)

Received previous seasonal influenza vaccine	
Year 2013–14	13 (87%)
Year 2014–15	14 (93%)
Year 2015–16	15 (100%)
Year 2016–17	15 (100%)
Year 2017–18	15 (100%)

Data are n (%) or median (IQR).

* Original answer choice converted to numeric scale where 1=excellent and 5=poor.

## Data Availability

Raw FASTQ sequencing files from Illumina MiSeq and PacBio sequencing have been deposited in the National Center for Biotechnology Information under the bioproject accession number PRJNA1023018. The variable heavy chain and variable light chain monoclonal antibodies have been deposited in GenBank with accession numbers GenBank OR621197–OR621290. The mass spectrometry proteomics data have been deposited to the ProteomeXchange Consortium via the PRIDE partner repository with the dataset identifier PXD046473. Cryo-electron microscopy structure coordinates for the UT14 Fab in complex with HA ectodomain have been deposited in the Protein Data Bank with accession number 8UZC, and the corresponding cryo-electron microscopy maps have been deposited in the Electron Microscopy Data Bank with accession code EMD-42839. Custom Python scripts used in bioinformatic analysis are available on formal request to the corresponding author.

## Research in context

### Evidence before this study

We searched PubMed using the terms (“influenza”) AND (“vaccine”) AND (“comparison”) AND (“egg”) AND (“cell” OR “recombinant”) for articles without language restrictions published between Jan 1, 2013, and Dec 8, 2023. We identified fewer than 30 research papers comparing the bulk serum responses induced by different vaccine platforms. A recent clinical study reported that recombinant haemagglutinin (HA)-based quadrivalent seasonal influenza vaccine (RIV4) induced higher neutralising antibody titres against vaccine strains compared with the standard-dose egg-based inactivated quadrivalent seasonal influenza vaccine (eIIV4) in health-care personnel aged 18–64 years during the 2018–19 influenza season. In separate studies, cell culture-based inactivated quadrivalent seasonal influenza vaccine (ccIIV4) and eIIV4 were reported to result in comparable neutralising antibody titres in adults (aged ≥18 years) and the elderly (aged ≥65 years). However, the key questions regarding whether and how antigenic and structural differences among the three licensed vaccines influence the degree of back-boosting, the IgG serological repertoire diversity, and, importantly, the binding affinity and breadth of circulating antibodies following vaccination have not been addressed.

### Added value of this study

Our study provides detailed molecular insights into how distinct vaccine platforms impact the identity, quantity, and functionalities of the IgG antibodies comprising the polyclonal antigen-specific serological repertoire. We showed that all three vaccine platforms overwhelmingly back-boost pre-existing clonotypes, resulting in serum antibody repertoires of comparable complexity with respect to diversity, VH somatic hypermutation, and heavy-chain complementarity determining region 3 biochemical properties. Notably, however, the H3/HA-specific serum response after RIV4 immunisation is dominated by antibodies with higher binding affinity to H3/HAs from viruses circulating since 2000, which, in turn, translate into higher bulk serum binding breadth to contemporary H3N2 strains. Further, we identified differential frequencies of stereotypical B-cell clonotypes associated with egg-glycan or membrane-proximal HA anchor epitope binding features among distinct vaccine cohorts. The analysis of stereotypical antibody sequences in the serum led to the identification of highly abundant monoclonal antibodies possessing a near stereotyped sequence for HA membrane anchor binding antibodies, yet binding to the HA trimeric interface, underscoring the subtleties in defining and interpreting stereotyped antibodies.

### Implications of all the available evidence

Our findings suggest that RIV4 elicits a serum response preferentially dominated by back-boosted antibodies with higher affinity and breadth towards contemporary H3N2 strains relative to the other two licensed vaccines, eIIV4 and ccIIV4. Our study reveals how seasonal influenza vaccination does not affect the diversity of the circulating antibody repertoire, which is heavily shaped by immune imprinting. Our data further suggest that subtle differences in vaccine immunogen arising from the production system can affect the level of different classes of stereotyped anti-HA antibodies.

## References

[1] McLean HQ, Belongia EA. Influenza vaccine effectiveness: new insights and challenges. Cold Spring Harb Perspect Med 2021; 11: a038315. 31988202 10.1101/cshperspect.a038315 PMC8168527

[2] Wang W, Alvarado-Facundo E, Vassell R, Comparison of A(H3N2) neutralizing antibody responses elicited by 2018–2019 season quadrivalent influenza vaccines derived from eggs, cells, and recombinant hemagglutinin. Clin Infect Dis 2021; 73: e4312–20. 32898271 10.1093/cid/ciaa1352

[3] Zost SJ, Parkhouse K, Gumina ME, Contemporary H3N2 influenza viruses have a glycosylation site that alters binding of antibodies elicited by egg-adapted vaccine strains. Proc Natl Acad Sci USA 2017; 114: 12578–83. 29109276 10.1073/pnas.1712377114 PMC5703309

[4] Raymond DD, Stewart SM, Lee J, Influenza immunization elicits antibodies specific for an egg-adapted vaccine strain. Nat Med 2016; 22: 1465–69. 27820604 10.1038/nm.4223 PMC5485662

[5] Jung J, Mundle ST, Ustyugova IV, Influenza vaccination in the elderly boosts antibodies against conserved viral proteins and egg-produced glycans. J Clin Invest 2021; 131: 148763. 10.1172/JCI148763 PMC8245176 34196304

[6] Guthmiller JJ, Utset HA, Henry C, An egg-derived sulfated N-acetyllactosamine glycan is an antigenic decoy of influenza virus vaccines. MBio 2021; 12: e0083821. 34126773 10.1128/mBio.00838-21 PMC8263001

[7] Izurieta HS, Chillarige Y, Kelman J, Relative effectiveness of cell-cultured and egg-based influenza vaccines among elderly persons in the United States, 2017–2018. J Infect Dis 2019; 220: 1255–64. 30561688 10.1093/infdis/jiy716

[8] Izurieta HS, Chillarige Y, Kelman J, Relative effectiveness of influenza vaccines among the United States elderly, 2018–2019. J Infect Dis 2020; 222: 278–87. 32100009 10.1093/infdis/jiaa080

[9] Dawood FS, Naleway AL, Flannery B, Comparison of the immunogenicity of cell culture-based and recombinant quadrivalent influenza vaccines to conventional egg-based quadrivalent influenza vaccines among healthcare personnel aged 18–64 years: a randomized open-label trial. Clin Infect Dis 2021; 73: 1973–81. 34245243 10.1093/cid/ciab566 PMC8499731

[10] Gouma S, Zost SJ, Parkhouse K, Comparison of human H3N2 antibody responses elicited by egg-based, cell-based, and recombinant protein-based influenza vaccines during the 2017–2018 season. Clin Infect Dis 2020; 71: 1447–53. 31598646 10.1093/cid/ciz996 PMC7486837

[11] Dunkle LM, Izikson R, Patriarca P, Efficacy of recombinant influenza vaccine in adults 50 years of age or older. N Engl J Med 2017; 376: 2427–36. 28636855 10.1056/NEJMoa1608862

[12] Naleway AL, Kim SS, Flannery B, Immunogenicity of high-dose egg-based, recombinant, and cell culture-based influenza vaccines compared with standard-dose egg-based influenza vaccine among health care personnel aged 18-65 years in 2019–2020. Open Forum Infect Dis 2023; 10: ofad223. 37305842 10.1093/ofid/ofad223 PMC10249269

[13] McDaniel JR, DeKosky BJ, Tanno H, Ellington AD, Georgiou G. Ultra-high-throughput sequencing of the immune receptor repertoire from millions of lymphocytes. Nat Protoc 2016; 11: 429–42. 26844430 10.1038/nprot.2016.024

[14] Lee J, Boutz DR, Chromikova V, Molecular-level analysis of the serum antibody repertoire in young adults before and after seasonal influenza vaccination. Nat Med 2016; 22: 1456–64. 27820605 10.1038/nm.4224 PMC5301914

[15] Lavinder JJ, Wine Y, Giesecke C, Identification and characterization of the constituent human serum antibodies elicited by vaccination. Proc Natl Acad Sci USA 2014; 111: 2259–64. 24469811 10.1073/pnas.1317793111 PMC3926051

[16] Li Z-N, Liu F, Gross FL, Antibody landscape analysis following influenza vaccination and natural infection in humans with a high-throughput multiplex influenza antibody detection assay. MBio 2021; 12: e02808–20. 33531397 10.1128/mBio.02808-20 PMC7858056

[17] Lee JM, Eguia R, Zost SJ, Mapping person-to-person variation in viral mutations that escape polyclonal serum targeting influenza hemagglutinin. eLife 2019; 8: e49324. 31452511 10.7554/eLife.49324 PMC6711711

[18] Wrammert J, Smith K, Miller J, Rapid cloning of high-affinity human monoclonal antibodies against influenza virus. Nature 2008; 453: 667–71. 18449194 10.1038/nature06890 PMC2515609

[19] Guthmiller JJ, Han J, Utset HA, Broadly neutralizing antibodies target a haemagglutinin anchor epitope. Nature 2022; 602: 314–20. 34942633 10.1038/s41586-021-04356-8 PMC8828479

[20] McCarthy KR, Lee J, Watanabe A, A prevalent focused human antibody response to the influenza virus hemagglutinin head interface. MBio 2021; 12: e0114421. 34060327 10.1128/mBio.01144-21 PMC8262862

[21] Bangaru S, Lang S, Schotsaert M, A site of vulnerability on the influenza virus hemagglutinin head domain trimer interface. Cell 2019; 177: 1136–52.e18. 31100268 10.1016/j.cell.2019.04.011 PMC6629437

[22] Watanabe A, McCarthy KR, Kuraoka M, Antibodies to a conserved influenza head interface epitope protect by an IgG subtype-dependent mechanism. Cell 2019; 177: 1124–35.e16. 31100267 10.1016/j.cell.2019.03.048 PMC6825805

[23] Bajic G, Maron MJ, Adachi Y, Influenza antigen engineering focuses immune responses to a subdominant but broadly protective viral epitope. Cell Host Microbe 2019; 25: 827–35.e6. 31104946 10.1016/j.chom.2019.04.003 PMC6748655

[24] Qiu Y, Stegalkina S, Zhang J, Mapping of a novel H3-specific broadly neutralizing monoclonal antibody targeting the hemagglutinin globular head isolated from an elite influenza virus-immunized donor exhibiting serological breadth. J Virol 2020; 94: e01035–19. 10.1128/JVI.01035-19 PMC7158729 31826999

[25] Henry C, Palm AE, Utset HA, Monoclonal antibody responses after recombinant hemagglutinin vaccine versus subunit inactivated influenza virus vaccine: a comparative study. J Virol 2019; 93: e01150–19. 31434733 10.1128/JVI.01150-19 PMC6803255

[26] Molari M, Eyer K, Baudry J, Cocco S, Monasson R. Quantitative modeling of the effect of antigen dosage on B-cell affinity distributions in maturating germinal centers. eLife 2020; 9: e55678. 32538783 10.7554/eLife.55678 PMC7360369

[27] Myers ML, Gallagher JR, Kim AJ, Commercial influenza vaccines vary in HA-complex structure and in induction of cross-reactive HA antibodies. Nat Commun 2023; 14: 1763. 36997521 10.1038/s41467-023-37162-z PMC10060936

[28] Kar U, Khaleeq S, Garg P, Comparative immunogenicity of bacterially expressed soluble trimers and nanoparticle displayed influenza hemagglutinin stem immunogens. Front Immunol 2022; 13: 890622. 35720346 10.3389/fimmu.2022.890622 PMC9204493

[29] Ueda G, Antanasijevic A, Fallas JA, Tailored design of protein nanoparticle scaffolds for multivalent presentation of viral glycoprotein antigens. eLife 2020; 9: e57659. 32748788 10.7554/eLife.57659 PMC7402677

[30] Tokatlian T, Read BJ, Jones CA, Innate immune recognition of glycans targets HIV nanoparticle immunogens to germinal centers. Science 2019; 363: 649–54. 30573546 10.1126/science.aat9120 PMC6420719

[31] Victora GD, Nussenzweig MC. Germinal centers. Annu Rev Immunol 2012; 30: 429–57. 22224772 10.1146/annurev-immunol-020711-075032

