# Supplementary material for: Molecular features of the serological IgG repertoire elicited by egg-based, cell-based, or recombinant haemagglutinin-based seasonal influenza vaccines: a comparative, prospective, observational cohort study
Source: Lancet Microbe. Author manuscript; Available in PMC 2025 Feb 10. (PMC11807745; doi:10.1016/j.lanmic.2024.06.002)
Supplement: Supplementary Material [file NIHMS2042160-supplement-Supplementary_Material.pdf]

THE LANCET
Microbe

Supplementary appendix

This appendix formed part of the original submission and has been peer reviewed.
We post it as supplied by the authors.

Supplement to: Park J, Bartzoka F, von Beck T, et al. Molecular features of the serological IgG repertoire elicited by egg-based, cell-based, or recombinant haemagglutinin-based seasonal influenza vaccines: a comparative, prospective, observational, cohort study. *Lancet Microbe* 2024. https://doi.org/10.1016/j.lanmic.2024.06.002

Appendix.

Molecular Features of the Serological IgG Repertoire Elicited by Egg-based, Cell-based, or Recombinant HA-based Seasonal Influenza Vaccines: a Comparative Prospective Observational Cohort Study

Juyeon Park, PhD[1,2+], Foteini Bartzoka, PhD[1+], Troy von Beck, PhD[4], Zhu-Nan Li, PhD[5], Margarita Mishina, BSc[5], Luke S. Hebert, BSc[1], Jessica Kain, MSc[2], Feng Liu, PhD[5], Suresh Sharma, PhD[5], Weiping Cao, PhD[5], Devon J. Eddins, PhD[5], Amrita Kumar, PhD[5], Jin Eyun Kim, PhD[3], Justin S. Lee, PhD[5], Yuanyuan Wang, PhD[5], Evan A. Schwartz, PhD[8], Axel F. Brilot, PhD[8], Ed Satterwhite, PhD[2], Dalton M. Towers, BSc[2], Eric McKnight, BSc[2], Jan Pohl, PhD[5], Mark G. Thompson, PhD[5], Prof. Manjusha Gaglani, MBBS[6], Fatimah S. Dawood, MD[5], Allison L. Naleway, PhD[9], James Stevens, PhD[5], Prof. Richard B. Kennedy, PhD[7], Prof. Joshy Jacob, PhD[4], Jason J. Lavinder, PhD[1,2], Min Z. Levine, PhD[5], Shivaprakash Gangappa, PhD[5], Gregory C. Ippolito, PhD[1*], Suryaprakash Sambhara, DVM PhD[5*], and Prof. George Georgiou, PhD[1,2,3*#]

1. Department of Molecular Biosciences, University of Texas at Austin, Austin, TX, USA
2. Department of Chemical Engineering, University of Texas at Austin, Austin, TX, USA
3. Department of Biomedical Engineering, The University of Texas at Austin, Austin, TX, USA
4. Department of Microbiology and Immunology, Emory Vaccine Center, School of Medicine, Emory University, Atlanta, GA, USA
5. Centers for Disease Control and Prevention, Atlanta, GA, USA
6. Baylor Scott & White Health, Baylor College of Medicine and Texas A&M University College of Medicine, Temple, TX, USA
7. Department of Medicine, Mayo Clinic, Rochester, MN, USA
8. Sauer Structural Biology Laboratory, Center for Biomedical Research Support, University of Texas at Austin, Austin, TX, USA
9. Kaiser Permanente Center for Health Research, Portland, OR, USA

[+] Equal contribution
* Senior authors
# Correspondence.

Table of contents.

1. Supplemental Methods.

1.1. Clinical Trial Approval and Study Design. 3
1.2. Microneutralization (MN) Assay. 3
1.3. Serum ELISA. 3
1.4. Circulating TFH Cell Responses. 3
1.5. Expression and Purification of Recombinant HA. 4
1.6. High Throughput Multiplex Influenza Antibody Detection Assay (MIADA). 4
1.7. Sample Preparation for Bulk VH-Only BCR-Seq. 4
1.8. Sample Preparation for Natively Paired VH:VL BCR-Seq. 4, 5
1.9. Bioinformatic Analysis of BCR-Seq. 5, 6
1.10. Sample Preparation for Bottom-Up Proteomics Analysis of Serum IgG Repertoires (Ig-Seq). 6, 7
1.11. Sample Injection to Liquid Chromatography Coupled with Tandem Mass Spectrometry (LC-MS/MS). 7
1.12. MS Searches for the Proteomic Identification of CDRH3 Peptides. 7, 8
1.13. Bioinformatic Analysis of Ig-Seq Proteomics Data. 8, 9
1.14. Calculation of Molecular Features of Serum Repertoires. 9
1.15. Design and Production of Antibody Expression Plasmids. 9, 10
1.16. MAb ELISA assay 10
1.17. Bio-Layer Interferometry for Kinetics and Epitope Binning Assay. 10, 11
1.18. Cryo-EM Sample Preparation and Imaging. 11
1.19. Cryo-EM Data Processing, Model Building, and Final Refinement. 11

2. Supplemental Figures and Tables.

2.1. Supplemental Figure 1. Clonotypic composition and relative abundance of pre- and post-vaccination serum IgG repertoires across RIV4, eIIV4, and ccIIV4 vaccine groups. 12, 13
2.2. Supplemental Figure 2. K-means clustering of vaccine cohort subjects. 14
2.3. Supplemental Figure 3. Comparison of VH SHM of clonotypes identified in the anti-H3/HA serological repertoire on day 28. 15
2.4. Supplemental Figure 4. VH gene usage of clonotypes identified in the H3/HA-specific serological repertoire on day 28. 16
2.5. Supplemental Figure 5. Circulating follicular helper T cells (cTFH) responses elicited by different vaccine platforms. 17
2.6. Supplemental Figure 6. Biochemical characterization of representative serum mAbs elicited by distinct vaccine platforms. 18
2.7. Supplemental Figure 7. H3/HA serum binding landscapes grouped by different vaccine cohorts 19
2.8. Supplemental Figure 8. Comparison of stereotypical anti-HA BCR clonotypes across three vaccine cohorts. 20
2.9. Supplemental Figure 9. Sequence information of UT14 and BLI epitope binning test 21
2.10. Supplemental Figure 10. Workflow for Cryo-EM data processing. 22
2.11. Supplemental Figure 11. Validation of UT14 – HA Cryo-EM structure. 23
2.12. Supplemental Figure 12. Map to model fit and the interaction of UT14 CDRH3 or CDRK3 with the HA antigen 24
2.13. Supplemental Table 1. Baseline characteristics of individuals in each vaccine cohort (n=15) 25
2.14. Supplemental Table 2. Representative serum mAbs selected for recombinant expression. 26, 27
2.15. Supplemental Table 3. Antigens used in multiplexed Luminex MIADA assay 28
2.16. Supplemental Table 4. Complete serum binding responses and breadth determined by multiplexed Luminex assay 29
2.17. Supplemental Table 5. Stereotypical sequence signatures of antibodies binding to public HA epitopes.....30
2.18. Supplemental Table 6. UT14–H3/HA Cryo-EM Statistics. 31

3. References. 32, 33, 34

1. Supplemental Methods.

Clinical Trial Approval and Study Design.

Eligible female healthcare personnel (n=15 subjects; n=5 per cohort) enrolled in a larger randomized trial were selected within the middle-aged group (mean ± SD; RIV4, 47·8 ± 4·7; eIIV4, 46·8 ± 9·5; ccIIV4, 48·2 ± 11·3 years) who received a single dose of quadrivalent cell-based inactivated vaccine (ccIIV4; Flucelvax™ Quadrivalent by Seqirus, Holly Springs, NC, USA; 15 µg of HA per strain), quadrivalent recombinant HA vaccine (RIV4; Flublok™ Quadrivalent by Sanofi Pasteur, Swiftwater, PA, USA; 45 µg of HA per strain), or standard-dose egg-based inactivated vaccine (eIIV4; Fluzone™ Quadrivalent by Sanofi Pasteur, Swiftwater, PA, USA; 15 µg of HA per strain) during the 2018-19 influenza season. The information regarding the trial design, study procedures, and participant details have been thoroughly described by Dawood et al.[1]

Briefly, this study is preplanned exploratory study of randomized open-label clinical trial (ClinicalTrials.gov; number NCT03722589) conducted among United States (US) healthcare personnel (HCPs) and aims to compare the molecular features of serological repertoire elicited by RIV4 or ccIIV4 compared to standard-dose eIIV4. The study protocol was reviewed and approved by the institutional review board (IRB) of the study site and Abt Associates, which provided site oversight and data management support. The IRB of the CDC relied upon the single IRB review of the BSWH IRB. Participants provided written informed consent before enrollment and trial participation. Laboratory investigators were blinded to vaccine arms until the completion of laboratory testing. Participants had sera collected just before vaccination and at approximately 28 days post-vaccination and had PBMCs collected on day 0 and day 7.

Microneutralization (MN) Assay.

The methodology for the microneutralization (MN) assay has been comprehensively described in Dawood et al.[1] Briefly, heat-inactivated sera was serially diluted by 2-fold in PBS and incubated with 100 tissue culture infectious dose per 50µl of cell-grown A/Singapore/ INFIMH-16–0019/2016 (H3N2) viruses at 37°C 5% CO_2 for 1 hour (hr) as previously described.[2] The cell-grown viruses were propagated in MDCK-SIAT1 cells (MilliporeSigma, Burlington, MA, USA, Cat 05071502). Subsequently, the MDCK cells were infected with the virus-sera mixtures, incubated for 18-20hr at 37°C 5% CO_2, and fixed using cold 80% acetone in PBS. MN titers were determined by calculating the reciprocal of the highest dilutions of serum that showed 50% virus neutralization.

Serum ELISA.

To measure the serum binding geometric mean titers (GMT), insect cell-expressed trimeric ectodomain HA from A/Singapore/INFIMH-16-0019/2016 (H3N2) was captured via C-terminus 6X-histidine tag on HisGrab™ Nickel coated plates (Thermo Fisher Scientific, Airport City, IL, USA) at 200 ng/well at 4 °C overnight. All sera were diluted in antibody diluent buffer (PBS containing 2 % (w/v) BSA (MilliporeSigma, Burlington, MA) and 0.05 % (v/v) Tween® 20 (MilliporeSigma, Burlington, MA, USA). An in-house control sera pool was included in each plate as an intra- and inter-assay control. All sera were 2-fold diluted in antibody diluent buffer starting from 1:800. The diluted sera were added to each well in duplicate, followed by incubation at room temperature for 1hr. The plates were washed with wash buffer (PBS containing 0·05 % (v/v) Tween® 20), and excess horseradish peroxidase (HRP)-labeled goat anti-human IgG (Kirkegaard & Perry Laboratories, Gaithersburg, MD, USA) was added to each well followed by incubation at room temperature for 1hr. The plates were washed with wash buffer, then SureBlueTM TMB Microwell Peroxidase Substrate (KPL, Gaithersburg, MD, USA) was added to each well, and after 4 minutes (min), the reaction was stopped with TMB stop solution (KPL, Gaithersburg, MD, USA). Plates were read at 450 nm with a SPECTRAmax plate spectrophotometer (Molecular Devices, San Jose, CA, USA). Serum ELISA IgG titer was defined by the reciprocal of the highest dilution that achieved OD450nm > 0·2 and > 2-fold higher than OD450nm of no serum control well.

Circulating TFH Responses.

cTFH cell responses (CD4+CXCR5+PD1+CD25-) were assessed by multiparametric flow cytometry via FACSymphony A5. 1-2·5x10^6 of PBMCs collected at day 0 and day 7. The following antibodies were used for cell staining: Live/Dead, BV570, Invivogen, San Diego, CA, USA, Cat L34959; CD3, BUV805, BD Biosciences San Jose, CA, USA, Cat 742053; CD45, BV480, BD Biosciences, Cat 566115; CD161, APC-R700, BioLegend, Cat 339942; TCR Vα7·2, BB700, BioLegend, Cat 351710; TCR Vδ2, BV605, BD Biosciences, Cat 331430; TCRγ/δ, APC, BioLegend, Cat 331212; CD4, BUV661, BD Biosciences, Cat 612963; CD8, BV786, BD Biosciences, Cat 563823; CXCR5, BB515, BD Biosciences, Cat 558112; PD1, BV650, BD Biosciences, Cat 564104; CD25, APC-Cy7, BD Biosciences, Cat 557753.

Expression and Purification of Recombinant HA.
Trimeric ectodomain and/or globular head HA1 antigens from influenza A viruses: pre-2009 A(H1N1), A(H1N1)pdm09, post-2009 A(H1N1), A(H2N2), A(H3N2), A(H5N1), A(H7N9), influenza B Victoria and Yamagata lineages, and NP from A(H3N2) were either obtained from International Reagent Resource or expressed using a in house baculovirus expression system.[3,4,5] See **Supplemental Table 3** for detailed strain information.

High Throughput Multiplex Influenza Antibody Detection Assay (MIADA).
Multiplexed MIADA assay was developed using HA, NP antigens, and a protein A (PA) control. The standard protocol (http://www.bio-rad.com/webroot/web/pdf/lsr/literature/4110012C.pdf) was optimized as described previously.[6,7,8] The human serum samples were diluted at 1:10,000, and recombinant mAbs were prepared at 300ng/ml in assay buffer (1× PBS with 0·05% Tween-20, 1% BSA, 0·5M NaCl, and 0·05% Sodium azide). Fifty microliters of microspheres containing two thousand microspheres for each bead region were resuspended in assay buffer and added to each well of a black wall plate (BD, San Jose, CA, USA) followed by 1hr incubation of properly diluted human sera or mAbs in duplicates. Two in-house serum pools were included on each plate as intra- and inter-assay controls. After washing with assay buffer, phycoerythrin (PE)-conjugated goat F(ab)2 anti-human IgG (Southern Biotech Inc, Brimingham, AL, USA) reporter was added to the plate, and the plate was incubated for one hour. After washing three times with 100µl of reading buffer (1× PBS with 0·05% Tween-20, 1% BSA, and 0·05% Sodium azide), plates were read by a Bio-Plex® MAGPIX™ Multiplex Reader. If the differences in median fluorescent intensity (MFI) in duplicates were more than 20%, the samples were re-tested. The limit of quantification for the MIADA assay was defined as the median plus two standard deviations of all negative blank controls. See **Supplemental Table 3** for antigen construct and strain information used in Luminex assay.

Sample Preparation for Bulk VH-Only BCR-Seq.
The bulk RNA was isolated from ≥ 1 million PBMCs on day 7. The cDNA was synthesized using 500ng of RNA, 1µl of 0·5µg/µl oligo dT, and 1µl of 10mM dNTP, and the final volume was brought up to 13µl. Primers were annealed at 65°C for 5min, and the reverse transcription (RT) reaction was performed using SuperScript™ IV First-Strand Synthesis System (Invitrogen, Carlsbad, CA, USA; Cat 18091050) by adding 4µl of 5x Superscript IV reverse transcriptase buffer, 1µl of 0·1M dithiothreitol (DTT), 1µl of ribonuclease inhibitor, and 1µl Superscript IV reverse transcriptase. The RT reaction was performed at 50°C for 10min, followed by 80°C for 10min. After adding 1µl of RNase H, samples were incubated at 37°C for 20min.

To amplify the VH amplicon, ten PCR reactions were prepared for IgG and IgA isotypes or for IgM isotypes as reported previously.[9] To amplify IgG and IgA VH transcripts, master mixes were prepared using FastStart™ High Fidelity PCR System (Roche, Indianapolis, IN, USA; Cat 3553400001) by adding 400µl of H_2O, 50µl of 10x FastStart high Fidelity reaction buffer, 12·5µl of each of forward primer sets and IgG/IgA reverse primer sets, 10µl of 10mM dNTP, and 5µl of FastStart High Fidelity enzyme blend. We used VH-only primer sequences described previously.[10] Master mixes for the IgM VH amplification followed the same recipe, with the exception of using IgM reverse primers as previously described.[10] To set up the reaction, 50µl of each master mix was transferred to one of the PCR tubes, which serves as a negative control with no template cDNA added. Then, the remaining 9µl of cDNA template was added to the master mixes, which were then split into nine different PCR tubes for 9 x 50µl PCR reactions. The PCR was performed as follows: 95°C for 2min; [92°C for 30 seconds (s), 50°C for 30s, 72°C for 1min] x 4 cycles; [92°C for 30s, 54°C for 30s, 72°C for 1min] x 4 cycles; [92°C for 30s, 63°C for 30s, 72°C for 1min] x 4 cycles; 72°C for 7min. After concentrating and purifying PCR products (Zymo Research, Tustin, CA, USA; Cat D4031), eluate was run on a 1% agarose gel to gel-purify the combined IgA and IgG amplicon bands or IgM amplicon bands. After gel extraction (Zymo Research, Tustin, CA, USA; Cat D4008), amplicons were eluted in 15µl of H_2O and subsequently sequenced on an Illumina MiSeq 2x300bp platform with a minimum of 1 million reads at the Genomic Sequencing and Analysis Facility (GSAF) of UT Austin Center for Biomedical Research Support following the attachment of Illumina adaptor barcodes.

Sample Preparation for Natively Paired VH:VL BCR-Seq.
Two million PBMCs were encapsulated in water-in-oil emulsions with oligo(dT)25 magnetic beads (New England Biolabs, Ipswich, MA, USA; Cat S1419S) and lysis buffer, containing 100mM Tris pH7·5, 500mM LiCl, and 10mM EDTA, using custom flow-focusing device as reported previously.[11] The subsequent RT-PCR and nested PCR reactions were performed as previously described.[11,12] Briefly, after single-cell emulsification, mRNAs from lysed cells were captured by the magnetic beads. The beads were rescued and re-emulsified for emulsion-based overlap extension (OE) RT-PCR using multiplexed primer sets, including (a) VH and VL framework region 1 overlap extension primers

appended with linker sequences, (b) constant region primers for IgM, IgG, and IgA isotypes heavy chains, and (c) constant region primers for lambda and kappa light chains. We previously reported the primer sequences required for paired VH:VL BCR-Seq.[12] The OE RT-PCR was carried out using the exonuclease-deficient version of thermostable reverse transcription xenopolymerase (RTX N210D) known for its exceptional resistance to cell lysate.[13] The RTX enzyme performs reverse transcription (RT) and cDNA amplification with potent proofreading capabilities. The reaction conditions were as follows: 68°C for 30min; 94°C 2min; [94°C for 30s, 60°C for 30s, and 68°C for 2min] x 25 cycles; 68°C for 7min. The OE RT-PCR products were rescued from the emulsion using hydrated ether. Any remaining ether was completely evaporated using speed-vacuuming (Eppendorf Vacufuge plus) at room temperature for 45min. The aqueous phase contains reverse-transcribed DNA products, which were then pooled into a new conical tube and diluted using 5x DNA binding buffer (Zymo Research, Tustin, CA, USA; Cat D4031) after the removal of poly dT magnetic beads.

A small-scale PCR cycle test was conducted to determine the optimal number of PCR cycles needed for nested PCR amplification as described previously.[11] Each test run included 0·5µl of template DNA, 0·5µl of 10mM dNTP, 1µl of 10µM nested primer mix,[12] 2·5µl of 10x ThermoPol Buffer (New England Biolabs, MA; Cat B9004S), 0·12µl of Platinum Taq DNA polymerase (Invitrogen, Carlsbad, CA, USA; Cat 15966005), and 20·5µl of H_2O. PCR was performed under the following conditions: 94°C for 2min; [94°C for 30s, 62°C for 30s, and 72°C for 20c] x for one of the 25, 30, and 35 cycles; 72°C for 7min. The optimal number of PCR cycles was selected based on the cycle number yielding the most clear gel bands without noisy smears around the target size. Next, four times scaled-up nested PCR was carried out using 5µl of template DNA, 10µl of nested primer mix,[12] 25µl of 10x ThermoPol buffer, 5µl of 10mM dNTP, 1·25µl of Platinum Taq polymerase (Invitrogen, Carlsbad, CA, USA; Cat 10966083), and 204µl of H_2O. The large-scale nested PCR were carried out using the optimal PCR cycle number determined above. The nested PCR products were PCR-cleaned up and gel-purified. This yielded physically linked VH:VL paired DNA amplicons with sufficient amount of DNA for the subsequent Illumina MiSeq and Pacific Biosciences HiFi long-read sequencing.

For the Illumina MiSeq sequencing, five different reactions were set up, each with a specific set of primers.[12] The reactions include: (a) IgG and IgA heavy chain isotypes forward primers in combination with kappa and lambda light chain reverse primers, (b) IgG and IgA heavy chain isotypes forward primers with VH framework 1 reverse multiplexed primers, (c) IgM heavy chain isotypes forward primers with kappa and lambda light chain reverse primers, (d) IgM heavy chain isotypes forward primers with VH framework region 1 reverse primers, and (e) kappa and lambda light chain forward primers with VL framework region 1 primers as previously described.[12] Each PCR reaction consisted of 25µl of NEB NEXT RXN mix (New England Biolabs, Ipswich, MA, USA; Cat M0541S), 4µl of forward primer, 4µl of reverse primer, and 30ng of template DNA, with a total reaction volume of 50µl. The PCR conditions were as follows: 98°C for 30s; [98°C for 10s, 62°C for 30s, and 72°C for variable extension time] for variable number of cycles, where the extension time and cycle parameters were as follows for each reaction: 33s, 8 cycles for reaction (a); 20s, 8 cycles for reaction (b); 33s, 5 cycles for reaction (c); 20s, 5 cycles for reaction (d), and 20s, 5 cycles for reaction (e).[12] Following the isotype-specific amplification, the final round of PCR was carried out to append the Illumina MiSeq barcodes to the PCR products. This step yielded amplicon bands of approximately 1100bp, 600bp, 1100bp, 600bp, and 550bp size for reactions (a) to (e), respectively. The five separate PCR products generated from day 7 PBMCs for each subject were submitted at GSAF UT Austin and sequenced on the Illumina Miseq 2x300bp platform, with a minimum of 1 million reads for each amplicon. For the Pacific Biosciences HiFi long-read sequencing, the nested PCR products were appended with two adapter sequences – "TATTCCCATCGCGGCGC" and "GCGCCGCGATGGGAATA" – to each end and then sequenced using Pacbio HiFi platform at the CDC site.

Bioinformatic Analysis of BCR-Seq.
Quality control (QC) was performed using PEAR v·0·9·6 software[14] for the raw fastq files containing VH-only or VL-only amplicon sequences. QC was carried out using a default setting and specific modifications: minimum overlap of 10, maximum assembly length of 700, and minimum assembly length of 50. This resulted in R1 and R2 stitched, paired-end reads that passed the QC threshold. The merged paired-end reads were subjected to V, D, and J annotation using the align and exportAlignments functions available in MiXCR software v·2·1·6.[15] MIXCR was also utilized to calculate the VH SHM and VL SHM rate (%) for each VDJ annotated read. To cluster the VH reads into individual clonotypes, a single-linkage hierarchical clustering was employed using a custom C programming language script, whereby VH sequences with a minimum read count of 2 and sharing at least 90% identity in the CDRH3 amino acid (aa) sequences, measured by Levenshtein distance, were clustered into the same clonal lineages (or clonotypes).

For the raw fastq files containing natively paired VH:VL amplicon sequences, QC step was carried out using Trimmomatic software v·0·27[16] with a default setting and additional specifications as follows: (i) implementation of a 5-base pair wide sliding window for read quality assessment, (ii) truncation of reads when the average quality per base drops below 20 (SLIDINGWINDOW:5:20), and (iii) removal of reads shorter than 100 bases (MINLEN:100). After removing the low-quality reads, R1 and R2 paired-end reads were stitched and VDJ-annotated using MIXCR. Subsequently, paired VH:VL BCR clustering was performed using USEARCH software 10·0·240,[17] in which the paired VH:VL sequences with a minimum read count of 2 and sharing ≥ 90% CDRH3 nucleotide identity were clustered into identical clonal lineages using a centroid-based UCLUST clustering algorithm.[18]

For the dataset from Pacific Biosciences sequencing, the single long-read fastq file was split into two sub-files, one for VH and the other for VL-only sequences. The complete long-read sequences containing both the VH and VL region and the appropriate PacBio adapter sequences were used in the downstream analysis. The MIXCR software 2·1·10 "align" function was used to annotate the V, D, and J regions. The annotated VH and VL sequences were then stitched together based on the matching read IDs, and the single-linkage hierarchical clustering algorithm was implemented to cluster the VH:VL paired reads based on a ≥ 90% Levenshtein identity within the CDRH3 amino acid sequences using the custom C programming language script as described above.

To identify clonotypes that possess conserved germline genes or motifs commonly observed in stereotypical anti-HA antibodies, we examined the published literature to collect the conserved genetic motifs that associate with egg-glycan binding, group 1 HA anchor-targeting, central-stem targeting, trimer-interface targeting, and multi-donor group 1+2 bNAbs. The clonotypic frequency for each class of stereotypical antibodies was determined by dividing the number of unique clonotypes matching with the known stereotyped genetic feature by the total number of clonotypes detected in each donor's day 7 BCR repertoire.

Sample Preparation for Bottom-Up Proteomics Analysis of Serum IgG Repertoires (Ig-Seq).
Circulating polyclonal IgG antibodies from pre-vaccination (day 0) and post-vaccination (day 28) serum were purified using Protein G Agarose (Thermo Fisher Scientific, Waltham, MA, USA; Cat 22852). Purified IgGs were then cleaved into F(ab)2 fragments by incubating with 100µg of IdeS protease (expressed in-house) per 5mg of IgG at 37°C for 2 hours. The 50mg of NHS-activated dry agarose resin (Thermo Fisher Scientific, MA; Cat 26196) was incubated with 1mg of HA antigens (A/Singapore/INFIMH-16-0019/2016; expressed from HEK cells, the custom order from Native Antigen Co) in 0·5ml of PBS overnight at 4°C. The HA-conjugated NHS resin was then incubated with 1 ml of 1M ethanolamine (pH 8·2) to block any empty active sites.

After blocking, the antigen-conjugated resin was packed into affinity column (Thermo Fisher Scientific, Waltham, MA, USA; Pierce Centrifuge Columns, 0·8mL; Cat 89868) and equilibrated with 12 column volumes (CV) of PBS by centrifuging at 1000g for 30s per spin. The cleaved polyclonal F(ab)2 fragments were added to the affinity column, and the column was sealed and incubated on an end-over-end rotator at room temperature for 1hr to allow the F(ab')2 fragments to associate with the HA antigens. The bottom cap of the affinity column was then removed and centrifuged at 1000g for 30s to collect the flow-through. The flow-through contains F(ab')2 fragments and Fc that did not bind to HAs. This step was repeated twice to remove any antibodies that are not specific to antigens. Subsequently, the affinity column was washed with 12 CVs of PBS at 1000g for 30s per spin.

For the affinity pull-down, HA-binding F(ab)2 fragments were eluted with 360µl of fresh 1% formic acid. This elution step was repeated six times to ensure the complete elution of HA-binding antibodies. For each elution step, 30µl of elution fraction was directly neutralized in a separate collection tube containing 20µl of 1M Tris buffer, pH 8·0. This allowed the test for pre-column, flow-through, and elution fractions in ELISA assay. Briefly, the ELISA plate was coated with the same HA antigen used in the affinity chromatography. By comparing the OD450nm among the pre-column, flow-through, and elution fractions from the ELISA test, we confirmed the depletion of HA-binding antibodies in the flow-through and validated that the affinity column was not oversaturated with antibodies.

To evaporate 1% formic acid (Fisher Scientific, Waltham, MA, USA; Cat A117-50), all elution fractions were speed-vacuumed for approximately 1·5hr at 45°C until less than 5µl of volume remained in each tube. The remaining volume was resuspended with 25µl of LC-MS-grade H_2O, followed by the addition of 8µl of 1M Tris pH 8·5. To ensure the complete neutralization, a small volume of 3M NaOH was added to adjust the pH into 7 using pH indicator strips. After pH adjustment, LC-MS-grade H_2O was added to each sample to bring the final volume into 50µl.

To denature the protein, an equal volume (50µl) of 99+% TFE (2,2,2-trifluoroethanol; Sigma-Aldrich, Taunton, MA, USA; Cat. T63002) was added to the eluate or flow-through containing approximately 25µg and 10µg of proteins, respectively. Samples were then reduced by adding TCEP (Bond-Breaker TCEP solution; Thermo Fisher Scientific, Waltham, MA, USA; Cat 77720) at a working concentration of 5mM. The mixture was then incubated at 55°C for 1hr. Subsequently, reduced samples were alkylated by iodoacetamide (Sigma-Aldrich, Taunton, MA, USA; Cat I1149) at a working concentration of 16·5mM at room temperature for 20min in the dark. Samples were then diluted 10-fold by adding 40mM Tris, pH 8, and the final volume was brought up 1ml. The denatured, reduced samples were then digested into tryptic peptides by incubating with 2µg of Trypsin Gold (Promega, Fitchburg, WI, USA; Cat V5280) overnight at 37° C on an end-to-end rotator. The tryptic peptides were speed-vacuumed for 4hr at 45°C until the final volume in each tube was evaporated down to less than 20µl. After the evaporation step, samples were resuspended in 0·1% formic acid solution and submitted to the University of Texas at Austin CBRS Biological Mass Spectrometry Facility for bottom-up protein identification by LC-MS/MS using Thermo Ultimate 3000 RSLCnano UPLC coupled to a Thermo Fisher Scientific Orbitrap Fusion Tribrid mass spectrometer.

Sample Injection to Liquid Chromatography Coupled with Tandem Mass Spectrometry (LC-MS/MS).
Before LC-MS/MS analysis, peptides were manually or robotically desalted using Millipore U-C18 ZipTip Pipette Tips (MilliporeSigma, Burlington, MA, USA; Cat ZTC18S096) following the manufacturer's protocol. The dried peptides were resuspended in 0·1% formic acid and transferred to the LC instrument autosampler. The samples were loaded onto a C18 trap column (2 cm × 75 µm inner diameter) for pre-concentration and desalting, then transferred to a C18 analytical column (25 cm × 75 µm inner diameter) packed with 3 µm material (Thermo Acclaim PepMap 100). A gradient from 5% to 45 % mobile phase B (0·1 % formic acid in acetonitrile) was used to elute peptides, increasing hydrophobicity over 100 min with a total run time of 120 min. MS data in positive mode were acquired in the orbitrap with the following settings: a 3-second cycle time, 120,000 resolution, scan range of 400-1600 m/z, maximum injection time of 60 milliseconds, and a 60 % RF lens value. A minimum signal intensity of 5E3 was required to trigger a data-dependent scan with charge states 2 to 6 inclusive. Dynamic exclusion properties were set to allow each precursor to be selected up to two times before being added to the dynamic exclusion list, with additional properties of +/- 25 ppm and 30-second exclusion. A targeted mass exclusion list containing the m/z values for 88 IgG constant region peptides was included. Centroid MS/MS data were acquired in the linear ion trap using quadrupole isolation followed by higher energy collisional dissociation (HCD) fragmentation. The settings for MS/MS acquisition included a 1·6 m/z isolation window, stepped HCD collision energies (27%, 31%, and 35 %), and a rapid ion trap scan rate with dynamic maximum injection time mode.

MS Searches for the Proteomic Identification of CDRH3 Peptides.
Experimental MS spectra were searched against the custom search database created by concatenating (1) ≥ 10^6 bulk VH-only sequences, (2) VH sequences from the paired VH:VL amplicon (reaction (b)), (3) VL-only sequences from the paired VH:VL amplicon (reaction (e)), (4) A/Singapore/INFIMH-15-0019/2016 HA antigen sequences, (5) Ensembl human proteome database, and (6) the list of common contaminant sequences as previously described.[9] Thermo Scientific Proteome Discoverer software v·1·4 was used for MS search. See the **supplemental method** section *"Sample preparation for Natively Paired VH:VL BCR-Seq"* for the information of reactions (b) and (e).

MS search was conducted using three different software sequentially as described previously.[19] First, Spectrum Selector was executed by applying the following settings: [precursor selection using MS1 precursor, use new precursor re-evaluation], and [spectrum properties filter to 0 except for the lowest charge state of 2]. Other settings include a minimum precursor mass of 600Da; maximum precursor mass of 6000Da; minimum peak count of 1; MS order of 2 (MS2); any activation type, minimum collision energy of 0; maximum collision energy of 1000; full scan type, nanospray ionization source; positive polarity mode; S/N threshold for peak filters set to 1; automatic unrecognized charge replacements; unrecognized MS order replacements to MS2; unrecognized activation type replacements to collision-induced dissociation, and unrecognized polarity replacements to +.

After specifying the precursor spectra for MS2, Sequest HT was executed with the following parameters: trypsin as a digestion enzyme; a maximum of 2 missed cleavage sites; minimum peptide length of 6; a maximum peptide length of 144; a maximum delta Cn of 0 for scoring options; reporting a maximum 10 peptides; a precursor mass tolerance of 5 ppm; a fragment mass tolerance of 0·5 Da; average precursor mass and fragment mass were not used (set to False), and neutral loss a, b, y, as well as flanking ions, were used. The weight values assigned to ions were set accordingly: a to 0, b to 1, c to 0, x to 0, y to 1, and z to 0, with a maximum of 3 equal modifications per peptide and a maximum

of 4 dynamic modifications per peptide. Dynamic modifications include oxidized methionine with a mass shift of +15·99995 Da, and static modifications included carbamidomethyl cysteine with a mass shift of +57·021Da.

Finally, Percolator (64Bit) was executed to improve the identification of reliable peptides. Percolator was run with the following parameters: a maximum delta Cn of 0·05; strict target false discovery rate of 0·01; relaxed target false discovery rate of 0·05, and a validation based on q-value. Throughout the MS search, the precursor ions quantifier node in Proteome Discoverer was set following the event detector node to determine the extracted-ion chromatogram (XIC) peak area for any quantifiable peptides.

Bioinformatic Analysis of Ig-Seq Proteomics Data.
We selected PSMs identified with high confidence for which the peptides were detected with a false discovery rate <1%. We excluded two or more peptides matching with the same spectrum due to their inherent ambiguity. Since leucine and isoleucine are indistinguishable by traditional tandem MS, both amino acid residues are treated as a single peptide group. After QC, PSMs originating from antibody VH regions and having an average mass deviation of less than 1·5 ppm were retained for downstream analysis.

The serum abundance of clonotype was calculated by summing all the unique extracted ion chromatogram (XIC) peak areas for CDRH3 peptides belonging to a given clonotype as previously described.[9,19,20] Any promiscuous CDRH3 peptides mapped to more than one clonotype were considered non-informative and excluded from the downstream analysis. After summing all the informative CDRH3 peptide PSMs and XIC areas for each clonotype, "5-fold cut-off" rule was applied to define the clonotypes specific to A/Singapore/INFIMH-16-0019/2016 HAs. The 5-fold cut-off samples serum clonotypes whose XIC abundance in the eluate is more than 5-fold higher than the XIC of the flow-through and defines such clonotypes as antigen-specific. Using this method, we evaluated the antigen-specificity for all clonotypes detected at each time point by comparing the abundance in the flow through versus eluate. To this end, clonotypes that passed the Elu/FT ratio ≥ 5-fold cut-off at least at one time point - either in the day 0 or in day 28 serum - were included in the final list of antigen-specific clonotypes to be analyzed, and, importantly, these clonotypes are considered as constituents of anti-A/Singapore/INFIMH-15-0019/2016 HA serological repertoire.

Relative abundance (%) of individual clonotypes identified in the serum repertoire was calculated by dividing the elution XIC area for a given clonotype by the total sum of the elution XIC area for all HA-specific clonotypes that have passed the 5-fold cut-off. To account for different serum binding GMT between day 0 and day 28 serum for each subject, the relative abundance (%) was normalized using the serum ELISA IgG titer measured on day 28 (day 28 GMT) as a reference. The normalized relative abundance is referred to as "relative amount" as described previously.[9] Specifically, the relative amount was calculated as follows:

- Day 0 relative amount (%) = Day 0 relative abundance (%) x (Day 0 GMT/ Day 28 GMT)
- Day 28 relative amount (%) = Day 28 relative abundance (%) x (Day 28 GMT/ Day 28 GMT) = Day 28 relative abundance (%). See the **supplemental method** "*Serum ELISA*" for the measurement of serum GMT to H3/HA Singapore 2016.

The relative amount of individual HA-specific clonotypes detected in the pre- and/or post-vaccination serum repertoire was plotted using red-colored heatmap. In the heatmap, each row corresponds to the same clonotype IDs for each donor. The grey color in the heatmap indicates that tryptic CDRH3 peptides belonging to a given clonotype were not detected in LC-MS/MS. Clonotypes with a relative abundance ≥ 0·5% at least on one time point were included in the heatmap or the repertoire histogram. Of note, the 0·5% cut-off was due to our empirical observations and the inherent limitations of traditional shotgun tandem proteomics experiments where the clonotypes with abundance below this 0·5% threshold are more prone to be stochastically sampled, which involve peptide desalting, speed vacuuming, and triplicate injections, etc.

To assess the impact of pre-existing immunity on humoral vaccine responses, we categorized day 28 clonotypes into two groups: (1) pre-existing and (2) newly-elicited clonotypes. Specifically, day 28 clonotypes detectable in the day 0 pre-vaccination serum are considered pre-existing, while clonotypes not detectable in the day 0 serum and only present in the day 28 serum are considered newly-elicited. It's important to note that this categorization was based on the presence or absence of clonotypes in the day 0 serum, regardless of HA antigen-specificity on day 0 before vaccination. For example, suppose clonotype A passes the 5-fold cut-off on day 28 but not day 0 due to higher abundance detected in the day 0 flow-through compared to the abundance in the day 0 eluate. In that case, this

particular clonotype will still be considered as pre-existing because it is indeed detectable in the day 0 serum ("pre-existing") despite its relatively lower abundance in the elution than in the flow-through.

Subject ID A5 and C4 showed an unexpectedly high fraction of newly-elicited antibodies in the post-vaccination serum repertoire. Thus, we termed these two subjects as "atypical" and analyzed their repertoires separately. The two atypical subjects were excluded in the repertoire analysis of typical donors. K-means clustering guided by silhouette scoring was used to assess the validity of the author's categorization of "typical" and "atypical" subjects based on (1) pre-existing clonal abundance in the day 28 serum repertoire and (2) changes in serum MFI to NP on day 28 relative to day 0. K-means clustering is a method for grouping multidimensional data into K discrete clusters. Silhouette scores measure how well samples are classified when assigned to said clusters according to both the tightness of the clusters and the separation between clusters, with a range from -1 (worst grouping) to 1 (best grouping). The KMeans and silhouette score functions from the Python3 package scikit-learn v·1·2·2) were used to iterate over cluster numbers from K = 2 to K = 10 and to generate corresponding silhouette scores in tandem. The highest silhouette score was used to conclude how many clusters best represent the suspected data groups (K = 2, silhouette score of 0·9).

Calculation of Molecular Features of Serum Repertoires.
To assess the diversity of the anti-HA serum Ab repertoires, we calculated the D80 diversity index for each time point and each donor. The D80 diversity index represents the frequency of clonotypes contributing to 80% of the total repertoire based on their abundance of XIC area. To calculate the D80 index, we sampled serum clonotypes with a relative abundance ≥ 0·5 % at each time point and recalculated their relative abundance based on XIC areas within the total XIC areas represented by the clonotypes passing the >0·5% threshold. The clonotypes were then sorted in descending order of relative abundance, and the number of top clonotypes, whose cumulative sum of abundance was ≤ 80 %, were divided by the total number of sampled clonotypes with a relative abundance ≥ 0·5%. We have confirmed a strong linear correlation between the calculated D80 diversity index and the Shannon entropy of the serum repertoire with ≥ 0·5 % relative abundance (Pearson correlation test: p-value < 0·0001, r = 0·94 [0·76–0·99]).

To calculate the weighted average of VH SHM for each clonotype, we averaged the SHM rates of VH sequences clustered and weighted them by the number of read counts for each VH sequence. The CDRH3 hydrophobicity was calculated based on Kyte and Doolittle index (Gravy index) by using the gravy argument in the Bio·SeqUtils·ProtParam module of the Biopython python package v1·72. To calculate the weighted average of the CDRH3 Gravy index for each clonotype, we averaged the GRAVY index of VH sequences clustered within the same clonotype and weighted them by the read counts for each VH sequence. The same weighted average method was applied to calculate representative CDRH3 aa length for each clonotype.

To assess the serum VH gene usage, we counted the number of VH genes used by individual clonotypes identified in the day 28 anti-HA repertoire and calculated their frequencies for each subject. This VH gene frequency analysis considered all anti-HA serum clonotypes detected, regardless of their relative abundance on day 28. To determine the VH gene abundance (in terms of XIC area), HA-specific serum antibody clonotypes were grouped based on the same VH gene usage, and the sum of abundance was calculated for each VH gene. We used the Scipy python package v·1·9·1 to perform Pearson correlation tests to evaluate the association between the increase in serum repertoire diversity (D80 index FC, day 28/day 0) and cTFH frequencies (FC, day 7/day 0).

Design and Production of Antibody Expression Plasmids.
The VH and VL aa sequences were codon-optimized for expression in Cricetulus griseus (hamster) using the IDT Codon Optimization Tool. The 5' and 3' restriction sites were added to each sequence for cloning into pFUSE expression plasmids. These sequences were synthesized as gBlock gene fragments (IDT, Coralville, IA, USA). EcoRI and NheI restriction sites were used to insert the VH gBlocks into the pFUSEss-CLIg-hgG1 vector (Invivogen, San Diego, CA, USA). For the light chain, EcoRI and BsiWI restriction sites were used to insert VL gBlocks into either pFUSE2ss-CLIg-hK (Invivogen, San Diego, CA, USA) or a modified pFUSE2ss-CLIg-hK vector bearing the human lambda light chain constant region in place of the kappa light chain constant region. Heavy and light chain pFUSE expression plasmids were grown in HB101 Escherichia coli (Zymo Research, Tustin, CA, USA; Cat T3011), purified by anion-exchange chromatography, and Sanger-sequenced to confirm the successful insertion of the target variable region.

To express mAbs, the paired heavy and light chain expression plasmids were co-transfected into Expi-CHO-S suspension cells (Thermo Fisher Scientific, Waltham, MA, USA; Cat A29127) at a 1:1 mass ratio using the

ExpiFectamine CHO Transfection Kit (Thermo Fisher Scientific, Waltham, MA, USA; Cat A29129) according to the manufacturer's instructions. The transfected cells were harvested after 10 days of culture in ExpiCHO Expression Medium (Thermo Fisher Scientific, Waltham, MA, USA; Cat A2910001) in 125mL polycarbonate Erlenmeyer flasks (Corning, Corning, NY, USA; Cat 431143) in a humidified cell incubator at 37°C, 8% CO_2 and on an orbital shaker with 19mm shaking diameter set to 125 rpm. Supernatants were collected by centrifugation at 12,000xg to remove any cellular debris. The supernatants were concentrated using a 50kDa molecular weight cut-off spin filter (MilliporeSigma, Burlington, MA, USA; Cat UFC9050) at 3,000xg centrifugation and buffer-exchanged with 1x PBS containing 0·02% Sodium azide. Before storage at -80°C, the concentration of each mAb in the supernatant was determined by nanodrop and quantitative ELISA using a human IgG control mAb.

MAb ELISA Assay.
H3/HA antigens were codon-optimized for expression in insect cells. HA antigens were expressed as trimers with a C-terminal Foldon motif and His-tag in a baculovirus expression system. HAs were expressed as full-length ectodomains. The secreted recombinant HA protein was recovered from the cell culture supernatant by tangential flow filtration through a 30kDa molecular weight cut-off membrane, followed by metal affinity chromatography and gel filtration chromatography. The H3 viral strains used to express HA ectodomain were as follows: A/Singapore/INFIMH-16-0019/2016 and A/Texas/50/2012 HA ectodomain.

For each recombinant flu antigen, 0·05µg of the antigen was dissolved in 10mL of 1x PBS. Next, 100µL of antigens was added to each well of a 96-well Maxisorp plate (Thermo Fisher Scientific, Waltham, MA, USA; Cat 442404). Plates were coated overnight at 4°C without shaking and subsequently washed 3 times with PBST and blocked at room temperature for 2hr with 200µL of PBST + 3% BSA. Serial dilutions of mAb supernatants were prepared in a separate plate, starting with a 10µg/ml concentration. The dilutions were made at a 1:5 ratio, while the bottom row always served as a PBST-only control. Then, 100µL of each mAb antibody dilution or the PBST control was added to allow association. The plates were incubated at room temperature with shaking for 90min, and the unbound monoclonal was removed by washing 3 times with PBST. Subsequently, 100µl of HRP-conjugated goat anti-hIgG secondary antibody (Southern Biotech, AL; Cat 2045-05) diluted 1:1000 in PBST was added to each well and incubated for 60min at room temperature with shaking. Following another round of washing with PBST, 50µL of TMB substrate (Thermo Fisher Scientific, Waltham, MA, USA; Cat N301) was added to each well and allowed to react for 2min prior to the addition of 25µL of 1M H2SO4. The negative cut-off was defined as the average absorbance value of the 12 PBST-only control wells plus two standard deviations. The endpoint titer for each mAb/antigen pair was determined as the lowest dilution with a 450nm reading above the negative cut-off. The half-maximal effective concentration (EC50) with 95% confidence interval estimate was calculated using a sigmoidal four-parameter logistic nonlinear regression model in GraphPad Prism v·10·2·1. The lower and upper limits of quantification for EC50 were defined as 1:5 (dilution factor) or 5:1 of the lowest or highest concentration of mAbs tested, respectively. M105, which displayed >10 µg/ml endpoint titers to all antigens tested, was colored in grey and excluded from downstream analysis. Low-affinity mAbs whose EC50 estimates are above the upper limit of quantification were assigned to an EC50 value of 100µg/ml. None of the mAbs tested fell below the lower limit of quantification.

Bio-Layer Interferometry for Kinetics and Epitope Binning Assay.
To express the Fab version of the UT14 IgG mAb, the stop codon was introduced in the IgG1 hinge region (EPKSCD*) of the heavy chain plasmids. The Fab expression construct was a kind gift from Professor Jason McLellan's lab at the University of Texas at Austin. Heavy and light chain plasmids were co-transfected into Expi293F cell lines at a 1:1 ratio. After 6 days, supernatants were harvested, and UT14 Fab was purified using CaptureSelect CH1-XL Affinity (Thermo Fisher Scientific, Waltham, MA, USA; Cat 1943462010) according to the manufacturer's instructions. The Octet RED96e system (ForteBio, Fremont, CA, USA) was used for kinetics and epitope binning assay. To measure the binding kinetics of UT14 Fab to A/Singapore/INFIMH-16–0019/2016 HA trimers or monomers, Octet FAB2G biosensors (Sartorius, Bohemia, NY, USA; Cat 18-5125) were equilibrated with PBS for 10min and loaded with 2µg/ml of UT14 Fab. The initial loading response was monitored until a 0·3nm shift was achieved within a 10-minute time window. After establishing a baseline in PBS for 1min, A/Singapore/INFIMH-16-0019/2016 HA monomer (Sino Biological, Wayne, PA, USA; Cat 40580-V08H) and A/Singapore/INFIMH-16-0019/2016 HA trimers (The Native Antigen Co, Oxford, UK) were 2-fold serially diluted from the initial concentrations of 1500nM and 3000nM, respectively, and associated with the UT14 Fab for 3min, followed by dissociation step in PBS for 5min. Kinetics measurement was repeated three times with a fresh biosensor tip for each run. K_D values were calculated using a 1:1 binding model in BIAevaluation software v·3·0.

For the epitope binning assay, Octet AHC2 Biosensor tips (Sartorius, Bohemia, NY, USA; Cat 18-5142) were equilibrated with PBS for 10min at room temperature and loaded with 45µg/ml of primary full-length IgG mAbs for 10min. Tips were then dipped into a fresh PBS well for 1min to establish a baseline and blocked using 50µg/ml of IgG isotype control (rituximab) for 5min. After blocking and 1min washing with PBS, 50µg/ml recombinant A/California/07/2009 HA monomer (Sino Biological, Wayne, PA, USA; Cat 11085-V08H) was loaded onto the tips for 5min to associate with the primary IgG mAbs. After washing and establishing the baseline in fresh PBS for 1min, tips were dipped into wells containing 45µg/ml of competing secondary IgG mAbs for 5min to test for epitope competition. After the competition, the tips were neutralized and regenerated in PBS and in 10mM glycine (pH 1·7) three times. For positive controls, D1 H1-3/H3-3[9,21], FluA-20[22], H2214[23], 047-09 4F04[24], FI6v3[25] and CR9114[26] were used.

Cryo-EM Sample Preparation and Imaging.
To obtain a high-resolution cryo-EM structure of the UT14-HA complex, a 2:1 molar ratio of UT14 Fab and A/Singapore/INFIMH-16-0019/2016 HA monomers were incubated at room temperature for 1hr and then 4°C overnight. The immune complexes were resuspended in a buffer containing 2mM Tris pH 8·0, 200mM NaCl, 0·02% NaN3 at a final concentration of 1mg/ml. A total of 3µl of the specimen was applied to Au-flat 1·2/1·3-hole pattern 300 mesh grids (Electron Microscopy Sciences, Hatfield, PA, USA; Cat AUFT313-50) that had been plasma cleaned in a PELCO easiGlow plasma cleaner (Ted Pella Inc, Redding, CA, USA) for 4min and was plunge-frozen into liquid ethane using Thermo Fisher/FEI Vitrobot Mark IV at 4°C under 100% humidity. Excess liquid was blotted for 4-6s. Cryo-EM was performed at the University of Texas at Austin Sauer Structural Biology Laboratory. The grids were imaged using a FEI Titan Krios G3 300kV cryo-TEM (Thermo Fisher Scientific, Waltham, MA, USA) equipped with a K3 direct electron detection camera (Gatan, Pleasanton, CA, USA) with a slit width 10eV Gatan BioContinuum Imaging Filter. A total of 3,198 micrographs were collected using SerialEM software at a magnification of 105000x with a stage tilt of 30° and an estimated defocus range of 0·91 – 2·55 µm. The calibrated pixel sizes were binned to 1·0415Å/pixel with a total dose of 80e/Å^2. See **Supplemental Table 6** for data collection statistics.

Cryo-EM Data Processing, Model Building, and Final Refinement.
The 3198 micrographs were motion-corrected using MotionCor2 (UCSF),[27] and CTF-estimation was performed using CryoSPARC v·4·2·1.[28] Exposures displaying CTF fit resolution (min: 1·67; max: 4·37) and relative ice thickness (min: 0·55; max: 1·16) were curated for further processing. Particle picking, 2D classification, 2D selection, *ab initio* reconstruction, and 3D refinement, including heterogeneous refinement and non-uniform refinement, were performed using CryoSPARC v4·2·1. The final refined 3D electron density map consists of 122281 particles with a global 3·8Å resolution and C1 symmetry. The initial H3 Singapore model was generated using AlphaFold v·2·3·2,[29] and the homology model for UT14 was generated using ABodyBuilder2.[30] The initial model was docked into the cryo-EM map using ChimeraX.[31] The model was iteratively refined using Coot v·0·9·8,[32] Phenix v·1·16,[33] and ISOLDE v·1·0·1.[34] Interface and contact analysis was performed with the PDBePISA interactive tool v·1·52 (https://www.ebi.ac.uk/pdbe/pisa/), ChimeraX v·1·5, and PyMOL v·2·5·5. The final volume sharpened map was exported from Cryosparc v·4·2·1 and flipped in ChimeraX, and the constant region of the Fab was removed. See **Supplemental Table 6** for Cryo-EM statistics. Residues 52A, 53, 55, and 56 of the UT14 heavy chain were set to alanine for fitting purposes.

Statistical analysis.
The normality of residuals was assessed using the D'Agostino-Pearson omnibus, Anderson-Darling, Shapiro-Wilk, and Kolmogorov-Smirnov tests. Visual inspection of the QQ plot was also conducted to further assess the assumptions of normality. Additionally, we performed the Brown-Forsythe and Bartlett's tests to assess the homogeneity of variances. Based on these tests, we used the following statistical tests for multiple comparisons: (1) Ordinary One-Way ANOVA, if the data passed all four normality tests ($P > 0.05$) and also passed the assumptions of equal SDs for both Brown-Forsythe and Bartlett's tests ($P > 0.05$), we used an ordinary one-way ANOVA followed by Tukey's multiple comparison tests; (2) Welch's ANOVA, if the data passed all four normality tests but did not pass the homogeneity of variance tests, we used Welch's ANOVA followed by Dunnett's T3 multiple comparisons test; (3) Kruskal-Wallis Test, if the data did not pass multiple normality and homoscedasticity tests, we used the non-parametric Kruskal-Wallis test followed by Dunn's multiple comparison tests.

2. Supplemental Figures and Tables.

Supplemental Figure 1. Clonotypic composition and relative abundance of pre- and post-vaccination serum IgG repertoires across RIV4, eIIV4, and ccIIV4 vaccine groups.

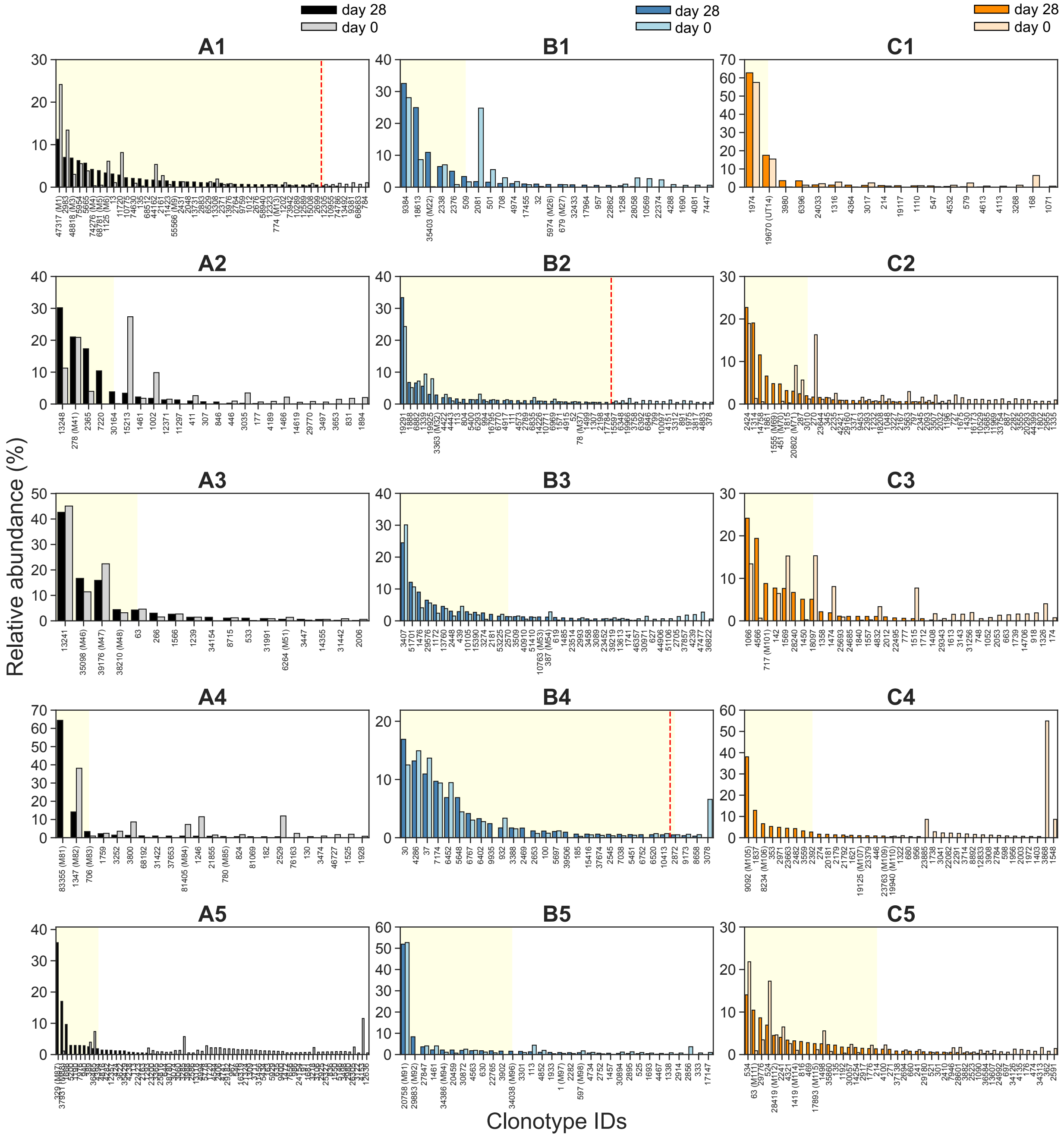

The serological repertoires specific to recombinant A/Singapore/INFIMH-15-0019/2016 (H3N2) HAs before and after vaccination with different types of vaccines that include RIV4 (left), eIIV4 (middle), and ccIIV4 (right) during the 2018-2019 influenza season. Subject IDs are shown on the top of each histogram. In each histogram, the x-axis represents unique clonotype IDs detected in the serum collected on day 0 and/or day 28 before and after vaccination, respectively. The representative mAbs selected from the corresponding clonotype IDs are shown inside the parenthesis. The y-axis displays the relative abundance of each clonotype at each time point (see **figure 1** legend for additional explanation). Clonotype IDs are listed in descending order of relative abundance at day 28 followed by the clonotypes detectable only at day 0. Only serum clonotypes with a relative abundance >0·5% are shown in the histogram. See the **supplemental method** for the Ig-Seq repertoire analysis. Yellow shade in the histogram indicates the day 28

clonotypes that account for 80% of the entire repertoire by abundance following vaccination (referred to as D80 diversity). For subjects A1, B2, and B4, the number of clonotypes comprising the 80% abundance of the repertoire falls below the 0·5% relative abundance cut-off in the histogram. As a result, the sum of the relative abundance of the day 28 clonotypes displayed in the histogram for these donors is less than 80%. To represent this, a red dotted line is included at the right edge of the yellow shade, indicating that the long tail of less abundant clonotypes is omitted in the histogram.

Supplemental Figure 2. K-means clustering of vaccine cohort subjects.

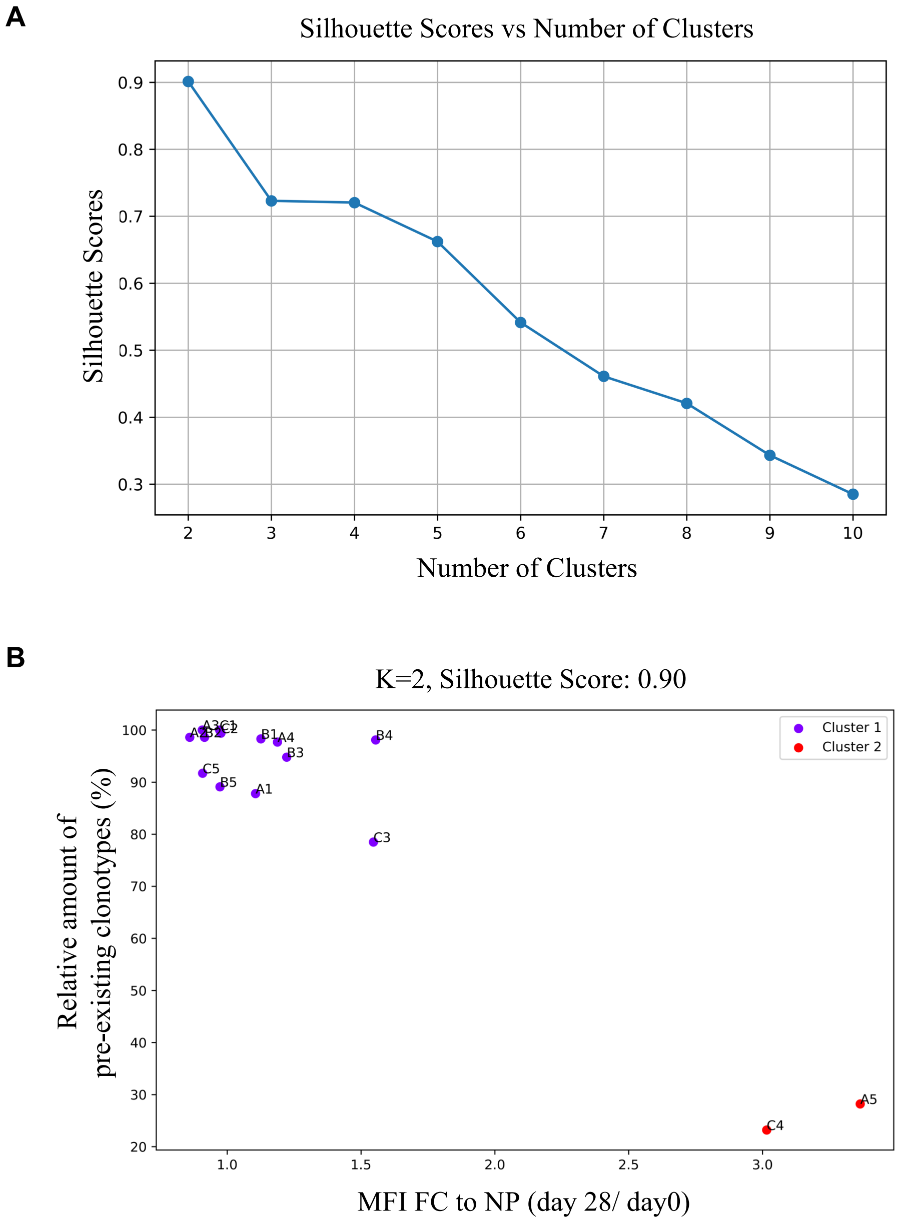

(A) The silhouette scores are generated from each cluster number K. **(B)** Subjects are plotted as labeled points according to their pre-existing clonotype abundance and the fold change (FC) in nucleoprotein titers. The highest silhouette score was 0·9 at K = 2, so these two groups are colored separately, with subjects A5 and C4 falling into their own group (colored in red, Cluster 2). Utilizing K-means clustering (guided impartially by silhouette scoring) of pre-existing clonotype prevalence and changes in NP titers suggests the subject data should be partitioned into two groups, with A5 and C4 in their own cluster and all the other subjects in another cluster.

Supplemental Figure 3. Comparison of VH SHM of clonotypes identified in the anti-H3/HA serological repertoire.

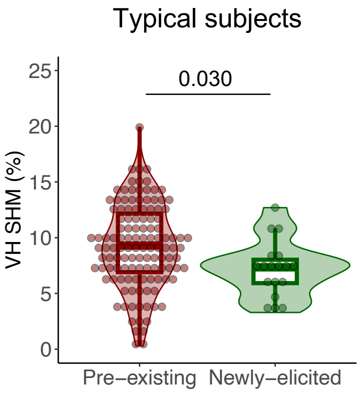

Analysis of VH SHM(%) for pre-existing and newly-elicited antibodies detected in the post-vaccination serum repertoires among typical donors, irrespective of the vaccine platform received. The violin and box-and-whisker plot were overlaid, and their schematics are described in the main method section. Statistical analysis was performed using a two-tailed unpaired t test.

Supplemental Figure 4. VH gene usage of clonotypes identified in the H3/HA-specific serological repertoire on day 28.

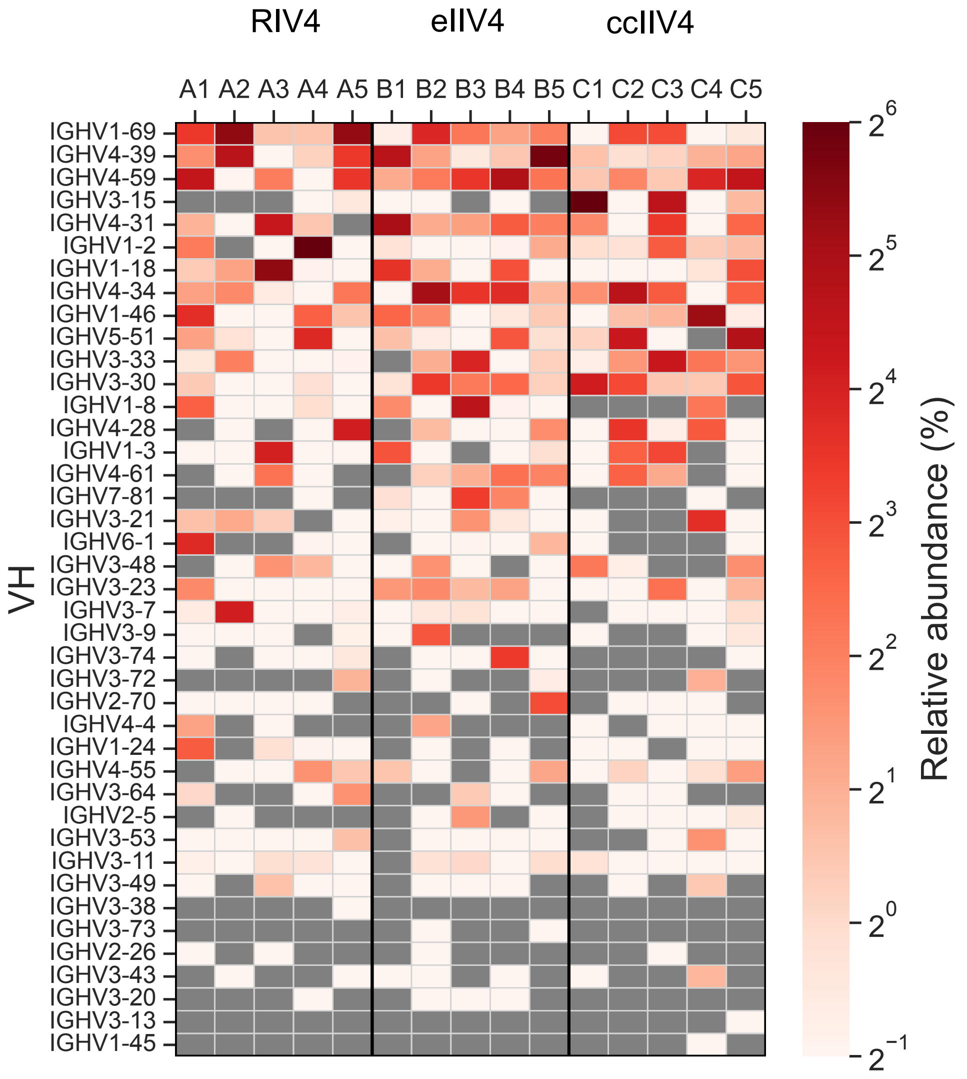

The serological abundance of VH genes in the serum IgG repertoire specific to A/Singapore/INFIMH-15-0019/2016 (H3N2) HA on day 28 following vaccination. The serum abundance of each IGHV gene was determined using the XIC peak area of LC-MS/MS. VH genes not found in the day 28 serological repertoire are shaded in grey. Each column represents each subject, with the VH gene usage of the top-most abundant clonotype highlighted with a blue boundary. VH genes on the vertical axis are sorted based on their mean abundance across n=15 subjects.

Supplemental Figure 5. Circulating follicular helper T cells (cTFH) responses elicited by different vaccine platforms.

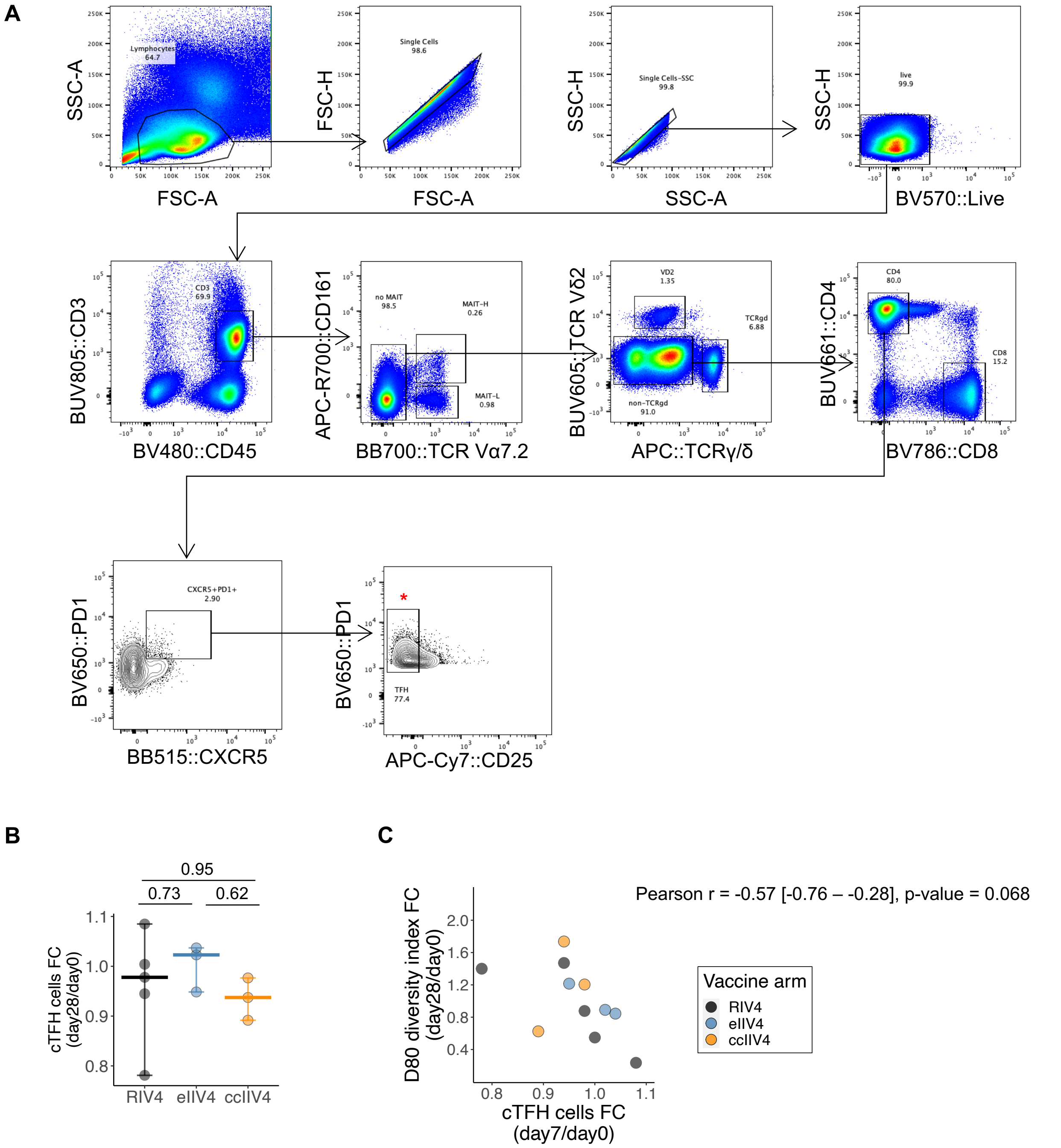

(A) Gating strategies for cTFH cells: CD3+CD4+CXCR5+PD1+CD25-. **(B)** Comparison of cTFH cells fold change (FC) on day 7 compared to day 0 across three different vaccine cohorts. The horizontal line and error bar indicate the median and 95% CI, respectively. **(C)** Changes in the diversity index of serological repertoire as a function of changes in circulating TFH cells (CD4+CXCR5+PD1+CD25-). cTFH responses were not measured for subject ID B2, B4, C2, and C3 due to the lack of sample availability. Statistical analysis was performed using an ordinary one-way ANOVA followed by Tukey's multiple comparisons test **(B)**. The Pearson correlation coefficients with 95% confidence interval estimates and p-value were shown **(C)**.

Supplemental Figure 6. Biochemical characterization of representative serum mAbs elicited by distinct vaccine platforms.

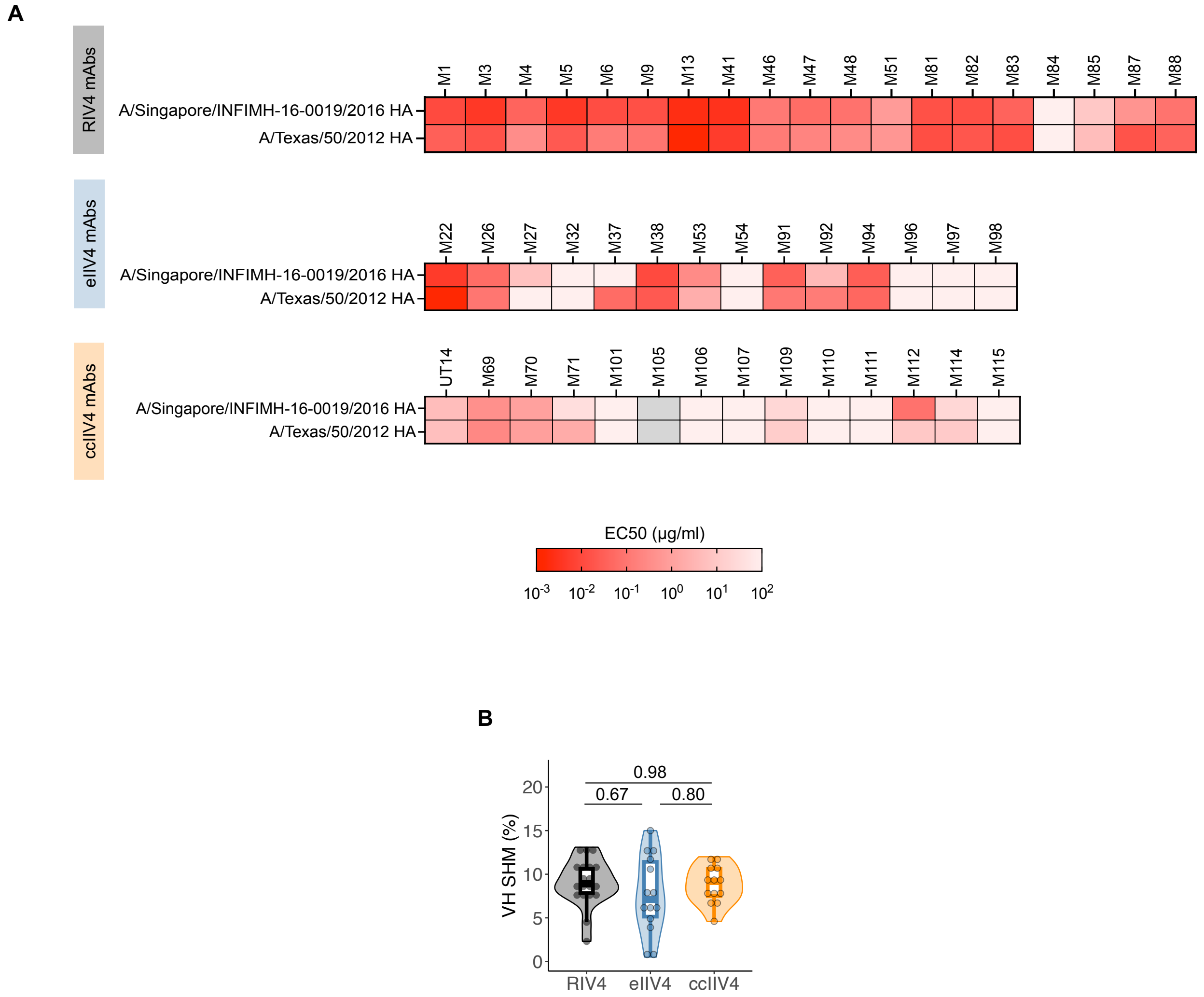

(A) Binding affinity of representative mAbs detected by serum proteomics of subjects immunized with RIV4 (top), eIIV4 (middle), and ccIIV4 (bottom) vaccines. M105, which displayed >10 µg/ml endpoint titers to all antigens tested, was colored in grey and excluded from downstream analysis. **(B)** Comparison of VH SHM (%) of mAbs across the three vaccine cohorts. The violin and box-whisker plot schematics are overlaid. For statistical analysis, Welch's ANOVA test followed by Dunnett's T3 multiple comparisons test was used for multiple comparisons among the three vaccine groups **(B)**.

Supplemental Figure 7. H3/HA serum binding landscapes grouped by different vaccine cohorts.

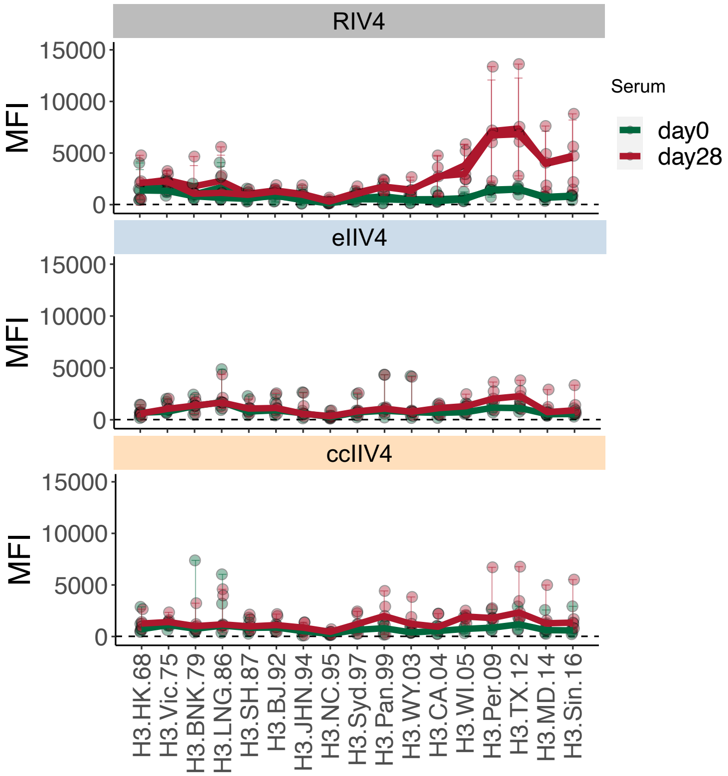

The serum IgG binding landscape to H3/HAs before and after vaccination. MFI was measured using 1:10,000 diluted serum at day 0 (green) or day 28 (red) via multiplexed Luminex assay. The line plot connects the median MFI across different HA antigens, and each dot represents individual subjects. The error bar shows the 95% confidence interval estimates of median for each antigen at a given time point. The dotted lines indicate the limit of quantification. See **Supplemental Table 4** for raw data.

Supplemental Figure 8. Comparison of stereotypical anti-HA BCR clonotypes across three vaccine cohorts.

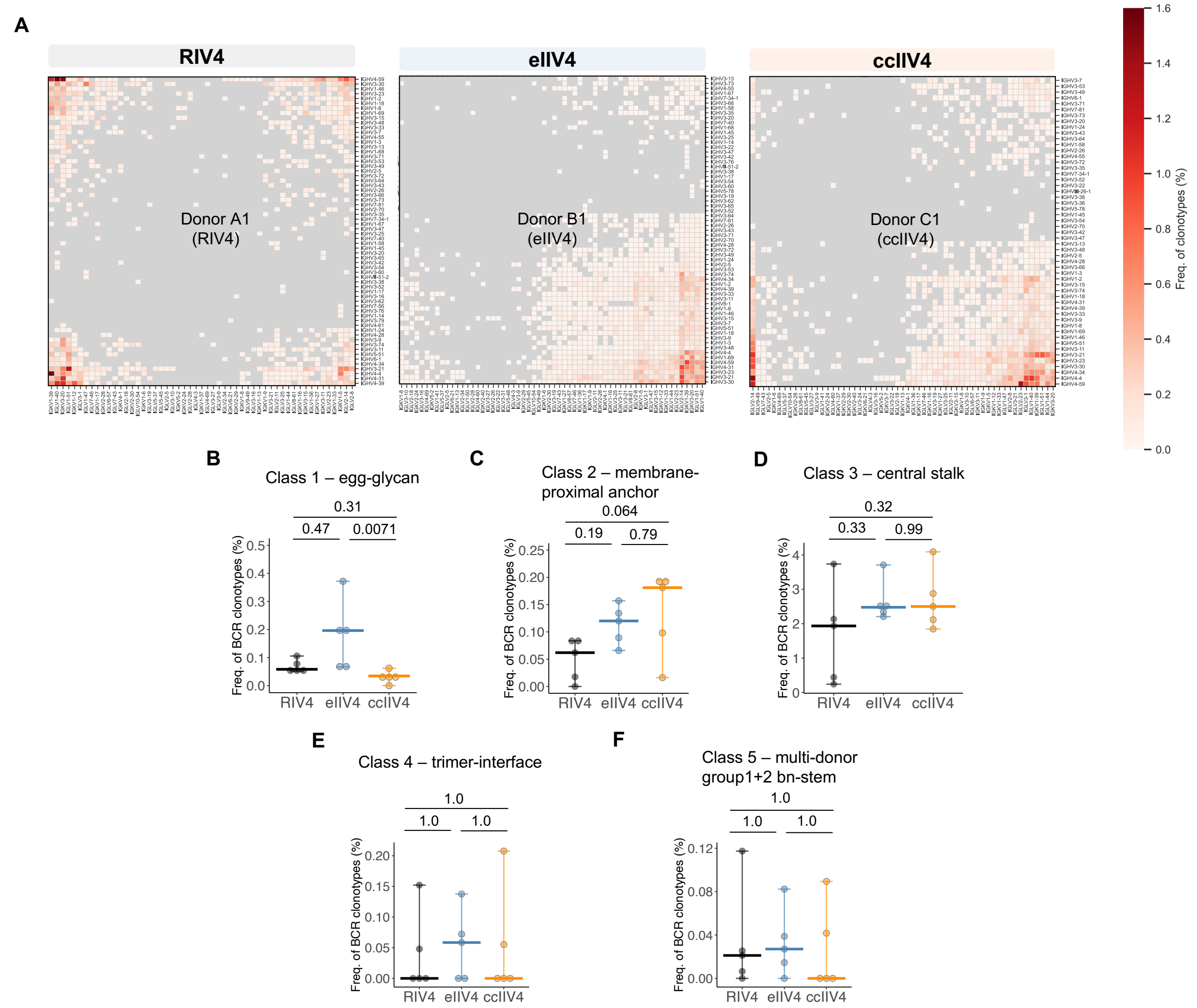

(A) Representative paired VH:VL usage of B cell receptor (BCR) repertoires in subjects A1, B1, and C1, immunized with RIV4 (left), eIIV4 (middle), and ccIIV4 (right), respectively. Subject IDs are indicated in the middle of the heatmap. The x-axis represents different VL gene usage, while the y-axis represents different VH gene usage. It should be noted that the order of VL or VH genes on the x-axis or y-axis, respectively, differs among the three represented heatmaps. The red color scheme represents the frequency of B cell clonotypes that utilize a specific combination of VH:VL pairs. Grey cells indicate the absence of clonotypes using such VH:VL combinations. **(B – F)** Comparative analysis on the frequencies of stereotypical BCR clonotypes sequenced by day 7 total B cells. Frequencies of BCR clonotypes matching the canonical sequence signatures reported from **(B)** egg-glycan targeting antibodies (Abs), **(C)** membrane-proximal anchor-targeting Abs, **(D)** central stalk-targeting Abs, **(E)** trimer-interface-targeting Abs, and **(F)** multi-donor group 1 and 2 broadly neutralizing Abs were compared across the three different vaccine cohorts. The horizontal line and error bar indicate the median and 95% CI, respectively. Each dot represents an individual donor. Statistical analyses were performed using Kruskal-Wallis tests followed by Dunn's post-hoc tests **(B, E, and F)** or ordinary one-way ANOVA tests followed by Tukey's post hoc tests **(C and D)** for multiple comparisons across the three vaccine groups.

Supplemental Figure 9. Sequence information of UT14 and BLI epitope binning test.

A

```
                                  IGHV3-30*18
Germline_VH    QVQLVESGGG VVQPGRSLRL SCAASGFTFS SYGMHWVRQA PGKGLEWVAV
UT14_VH        .......... .......... ...T...... ...I...... .....G...M

Germline_VH    ISYDGSNKYY ADSVKGRFTI SRDNSKNTLY LQMNSLRAED TAVYYCAKXX
UT14_VH        ..F...KT.. ....R..... .........S .......T.. ........ER

               IGHD5-24*01  IGHJ4*02
Germline_VH    XRDGYNXXXX DYWGQGTLVT VSS
UT14_VH        D.....EGIY .......... ...
```

B

```
                                  IGKV3-11*01
Germline_VK    EIVLTQSPAT LSLSPGERAT LSCRASQSVS SYLAWYQQKP GQAPRLLIYD
UT14_VK        ...M...... .......... ........AG F......... ..........
                                                             IGKJ5*01
Germline_VK    ASNRATGIPA RFSGSGSGTD FTLTISSLEP EDFAVYYCQQ RSNWPITFGQ
UT14_VK        T......... ....R..... .....N.... .......... .Y........

Germline_VK    GTRLEIK
UT14_VK        .......
```

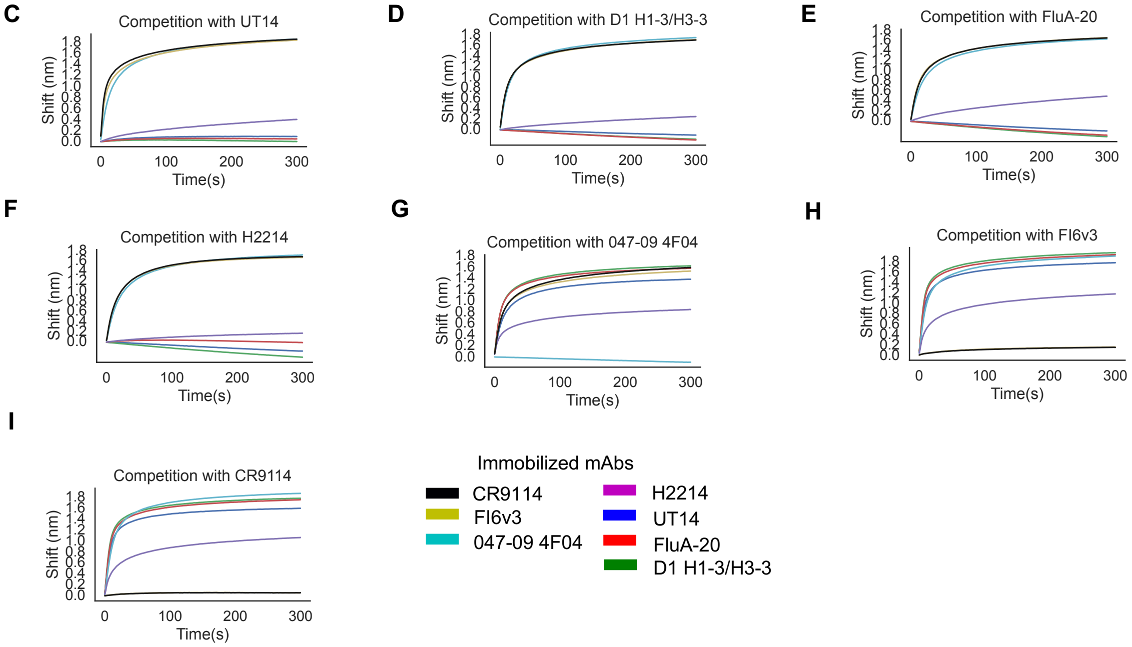

Sequence alignment of UT14 VH (A) and VK **(B)** amino acid sequences against IGHV3-30*18+IGHD5-24*01+IGHJ4*02 and IGKV3-11*01+IGKJ5*01 germlines, respectively. UT14 sequence matching known stereotypical motifs for membrane-proximal anchor Abs were shaded in red. **(C – I)** The raw BLI sensorgram from epitope competition binning test. Primary mAbs were immobilized on the tip of the anti-hIgG Fc capture (AHC) biosensor, followed by association with the A/California/2009 (H1N1) HA protomer. Subsequently, secondary mAbs (shown as a title for each sensorgram) were associated with the biosensor to determine the competition for binding to H1/California/07/2009 HA.

Supplemental Figure 10. Workflow for Cryo-EM data processing.

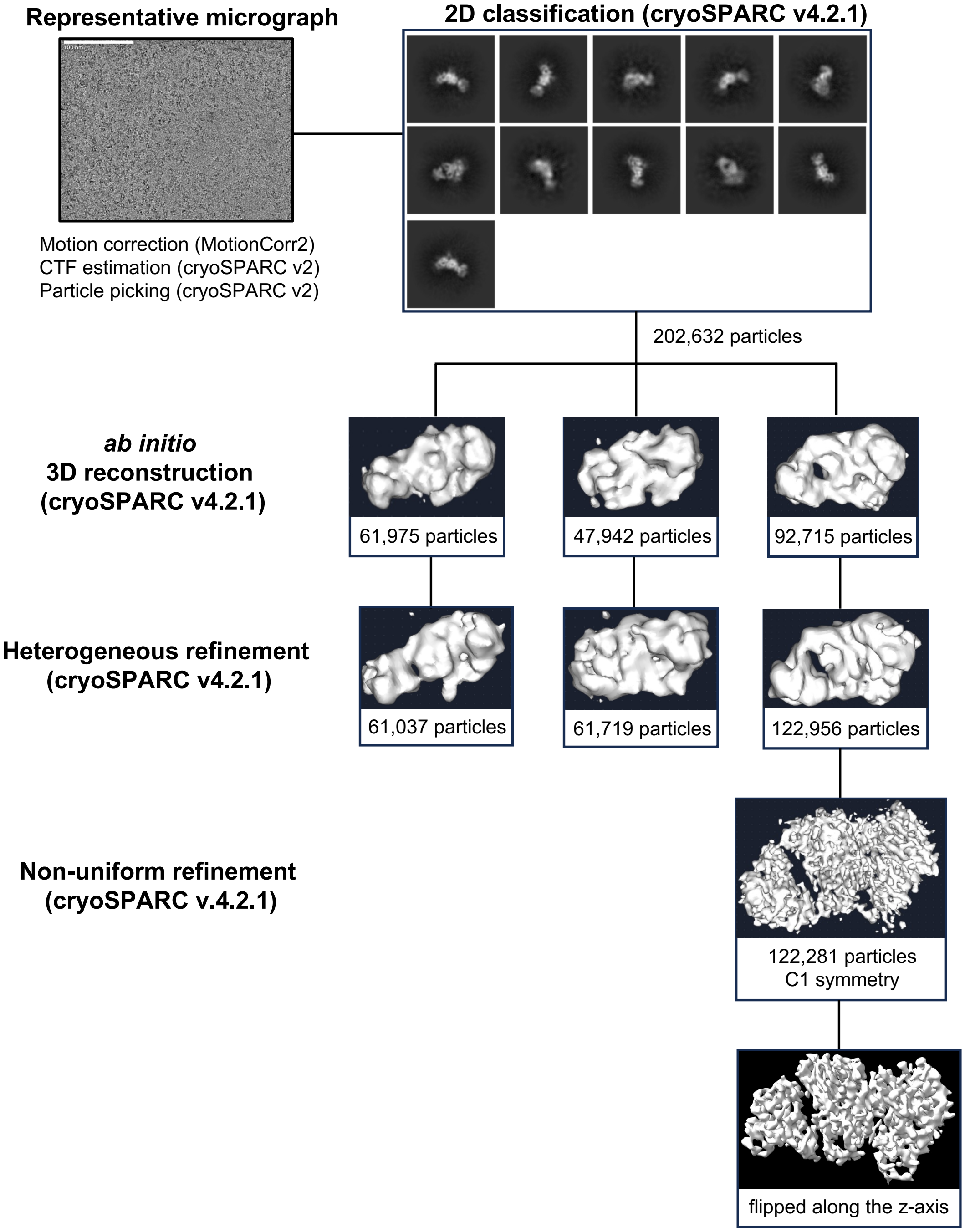

Cryo-EM Data processing workflow for UT14 Fab – HA structure.

Supplemental Figure 11. Validation of UT14 – HA Cryo-EM structure.

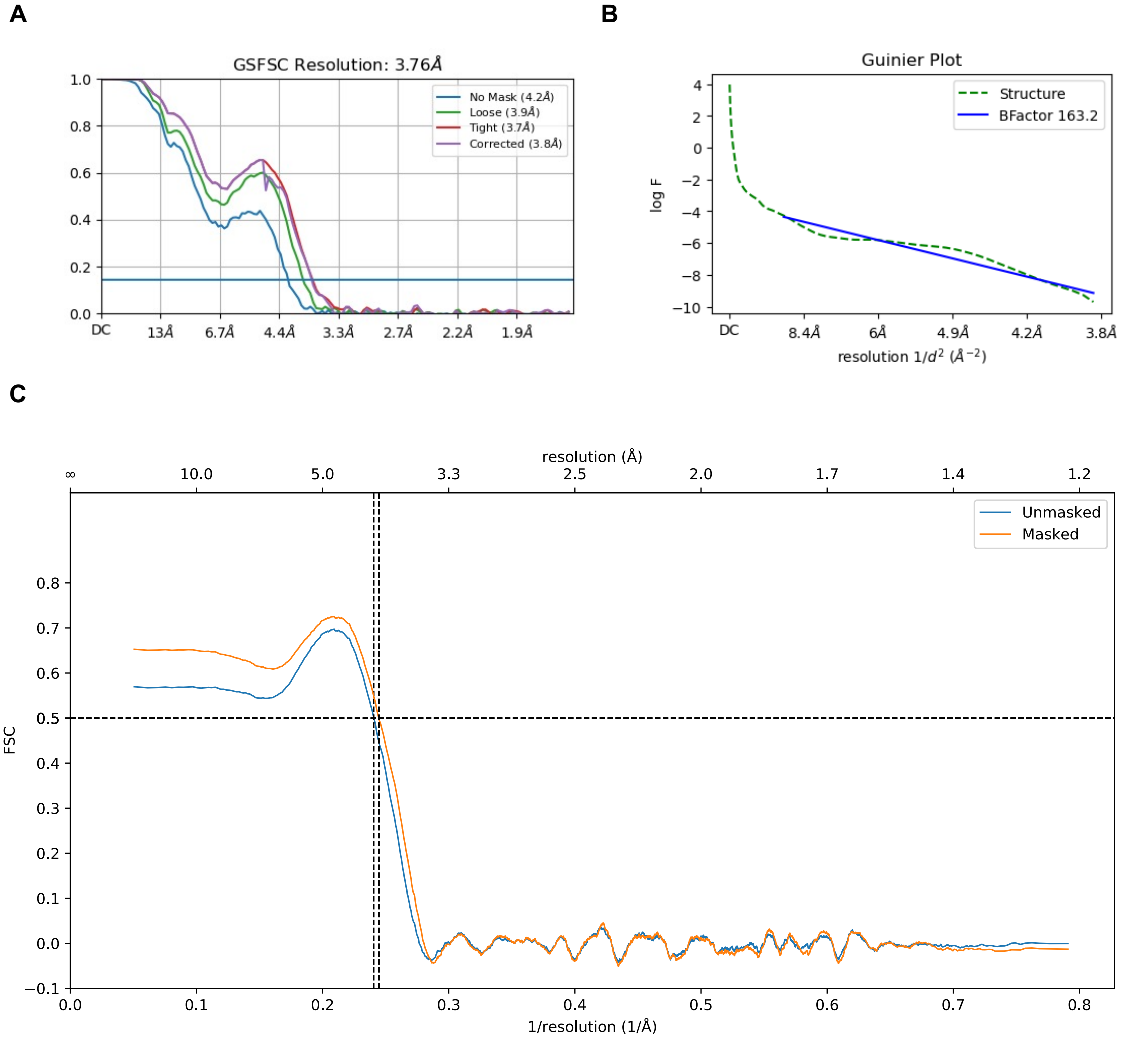

(A) FSC plot of the UT14_H3Singapore map generated in cryoSPARC v·4·2·1. **(B)** Guinier plot for b-factor sharpening. **(C)** Map-to-model resolution at an FSC of 0.5.

Supplemental Figure 12. Map to model fit and the interaction of UT14 CDRH3 or CDRK3 with the HA antigen.

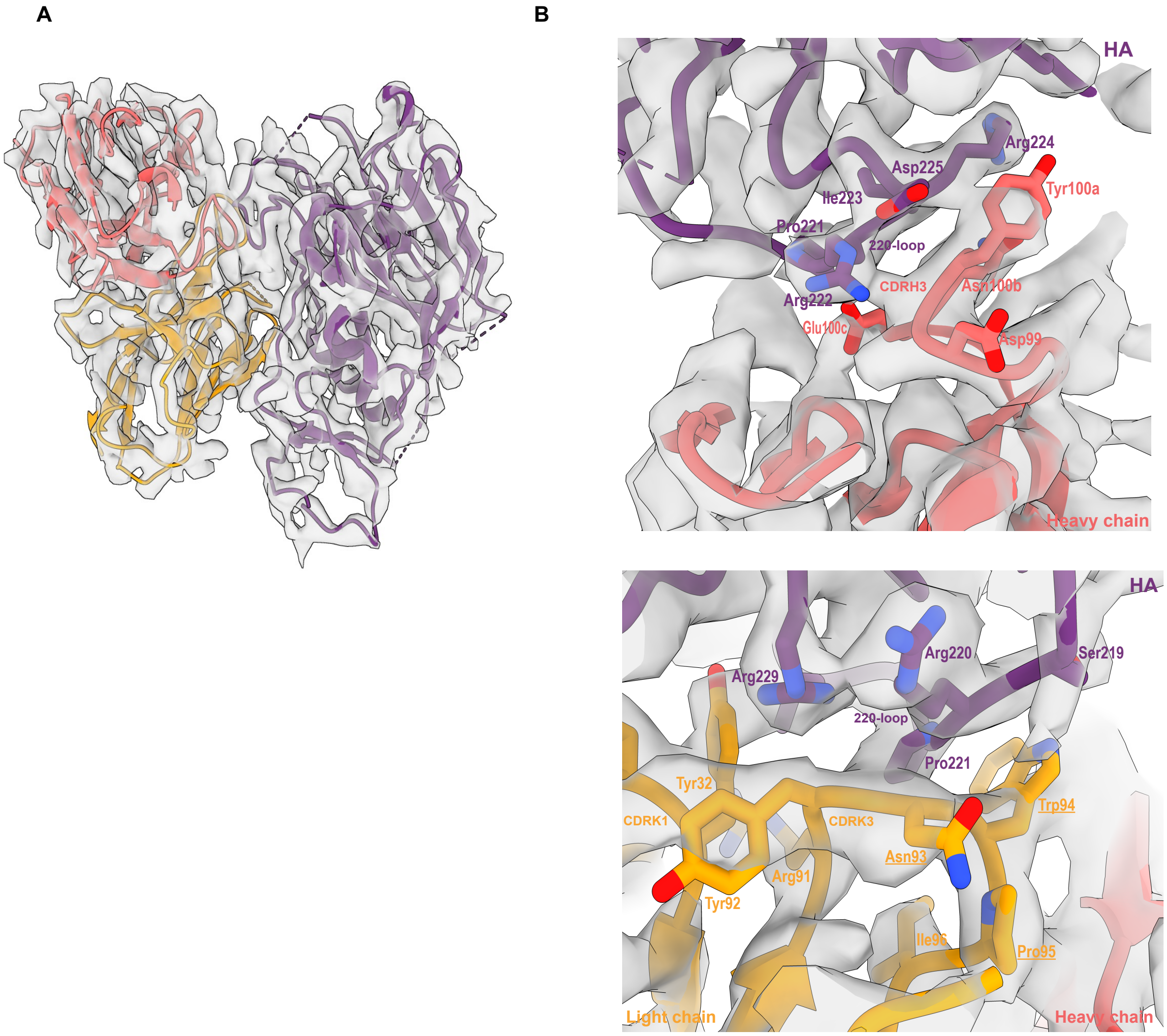

(A) UT14 – HA (purple) model fitted into the Cryo-EM density map. **(B)** Close-up views of UT14 CDRH3 (top, pink) and CDRK3 (bottom, yellow) paratopes and their cognate epitope interface.

Supplemental Table 1. Baseline characteristics of individuals in each vaccine cohort (n=15)

Individual ID	Vaccine cohort	Age, years	Birth year	Sex	Previous seasonal vaccinations as per electronic medical records	Peripheral mononuclear cells	Serum
A1	RIV4	52	1966	Female	5	Day 7	Day 0, day 28
A2	RIV4	41	1977	Female	5	Day 7	Day 0, day 28
A3	RIV4	45	1973	Female	5	Day 7	Day 0, day 28
A4	RIV4	51	1967	Female	5	Day 7	Day 0, day 28
A5	RIV4	50	1968	Female	5	Day 7	Day 0, day 28
B1	eIIV4	59	1959	Female	5	Day 7	Day 0, day 28
B2	eIIV4	40	1978	Female	4	Day 7	Day 0, day 28
B3	eIIV4	42	1976	Female	3	Day 7	Day 0, day 28
B4	eIIV4	38	1980	Female	5	Day 7	Day 0, day 28
B5	eIIV4	55	1963	Female	5	Day 7	Day 0, day 28
C1	ccIIV4	60	1957	Female	5	Day 7	Day 0, day 28
C2	ccIIV4	56	1962	Female	5	Day 7	Day 0, day 28
C3	ccIIV4	31	1987	Female	5	Day 7	Day 0, day 28
C4	ccIIV4	45	1973	Female	5	Day 7	Day 0, day 28
C5	ccIIV4	49	1968	Female	5	Day 7	Day 0, day 28

Supplemental Table 2. Representative serum mAbs selected for recombinant expression.

Subject ID	Vaccine	Clonotypes				Representative monoclonal antibodies (mAbs)							
		clonotype ID	Serum abundance (%) day 28	XIC	PSM	mAb ID	VH	DH	JH	CDRH3_aa	VL/VK	JL/JK	CDRL/K3_aa
A1	RIV4	47317	11.270	2.33E+09	46	M1	IGHV1-46	IGHD3-10	IGHJ4	CARRQNFYNSGSPLDYW	IGLV1-51	IGLJ3	CGTWDFRLNNWVF
A1	RIV4	48818	6.849	1.42E+09	29	M3	IGHV6-1	IGHD2-15	IGHJ2	CAGSPGACSGDRCYSALLTHFSYFNLW	IGLV1-44	IGLJ2	CAAWDDSLIGPLF
A1	RIV4	74276	4.182	8.66E+08	37	M4	IGHV4-59	IGHD6-13	IGHJ4	CARHGAASSCDHW	IGLV1-40	IGLJ1	CQSYDSSLSGYVF
A1	RIV4	68781	3.900	8.07E+08	14	M5	IGHV4-4	IGHD5-24	IGHJ4	CAREGDGYNSVLDYW	IGKV1-13	IGKJ4	CLQFYDYPLTF
A1	RIV4	1125	3.353	6.94E+08	10	M6	IGHV3-23	IGHD3-22	IGHJ4	CAKGGETYDYDSSGFIPHYW	IGKV1-12	IGKJ4	CQQANSFPLTF
A1	RIV4	55566	1.357	2.81E+08	5	M9	IGHV1-69	IGHD1-14	IGHJ2	CATDPRTSAWVFRYFDLW	IGLV3-21	IGLJ2	CQVWDSSSDHVVF
A1	RIV4	774	0.558	1.15E+08	15	M13	IGHV3-64	IGHD2-2	IGHJ4	CARDGLGRDGFCSSTSCFGGFDSW	IGLV3-25	IGLJ1	CQSADSTGTYVF
A2	RIV4	278	21.040	7.94E+08	40	M41	IGHV4-39	IGHD3-10	IGHJ5	CARQVGDTMVRGVISDWFDTW	IGLV3-1	IGLJ2	CQAWDSTAKALVF
A3	RIV4	35098	16.686	1.16E+09	174	M46	IGHV1-3	IGHD6-13	IGHJ6	CARDQQDLVHPYYYYYYMDVW	IGKV3-15	IGKJ2	CQHYHEWPPGMYTF
A3	RIV4	39176	15.913	1.11E+09	68	M47	IGHV4-30	IGHD1-26	IGHJ3	CARAYIESLYSKVGSFDVW	IGKV1-39	IGKJ1	CQQSYSAPLTF
A3	RIV4	38210	4.422	3.08E+08	20	M48	IGHV4-31	IGHD4-17	IGHJ3	CARGGFDYGDPLPRPDGFEFW	IGKV3-20	IGKJ1	CQYHGNSPWTF
A3	RIV4	6264	0.643	4.48E+07	8	M51	IGHV3-11	IGHD6-13	IGHJ4	CAGGIRGFSSSNFDYW	IGLV2-14	IGLJ2	CSSFSSTTTLVVF
A4	RIV4	83355	64.419	2.70E+09	110	M81	IGHV1-2	IGHD6-19	IGHJ4	CARSDRSGWFSDHW	IGLV3-25	IGLJ3	CQSADSGGIWVF
A4	RIV4	1347	14.171	5.93E+08	24	M82	IGHV5-51	IGHD1-26	IGHJ4	CASSLEKVGTVFGYFDYW	IGKV4-1	IGKJ2	CQQYHSTPYTF
A4	RIV4	706	3.384	1.42E+08	8	M83	IGHV1-46	IGHD4-17	IGHJ6	CARVSLNPTRQIYGMDVW	IGLV3-25	IGLJ2	CQSTETNGPYVIF
A4	RIV4	81405	0.913	3.82E+07	3	M84	IGHV4-30	IGHD3-3	IGHJ4	CARGGGGYYKELDYW	IGKV1-5	IGKJ2	CQQYNWYQKTF
A4	RIV4	780	0.673	2.82E+07	7	M85	IGHV1-46	IGHD1-14	IGHJ3	CVRAPDQAFDMW	IGKV2-24	IGKJ1	CMQATQFPRTF
A5	RIV4	329	35.805	1.74E+09	144	M87	IGHV1-69	IGHD3-16	IGHJ5	CAGERGHCANFDNCLHPDWFDPW	IGLV1-44	IGLJ3	CATWDDGLNGWVF
A5	RIV4	37931	17.019	8.26E+08	137	M88	IGHV4-38	IGHD3-22	IGHJ5	CATWHSSGYYEFPHSDWFDPW	IGLV1-40	IGLJ2	CQSFDSSLSAVVF
B1	eIIV4	35403	10.926	7.50E+07	23	M22	IGHV1-18	IGHD2-15	IGHJ6	CTRGKDTATVLDFYSGMDVW	IGLV3-25	IGLJ1	CQSVDSSGTYVF
B1	eIIV4	5974	0.839	5.75E+06	3	M26	IGHV4-4	IGHD6-13	IGHJ4	CARRYSSSWLGDW	IGKV1-12	IGKJ4	CQQASSFPLTF
B1	eIIV4	679	0.832	5.71E+06	4	M27	IGHV4-31	IGHD6-6	IGHJ6	CARDRGSSAVLSGMDVW	IGKV3-11	IGKJ4	CQQRSNWPLTF
B2	eIIV4	3363	2.832	9.14E+06	1	M32	IGHV3-23	IGHD3-10	IGHJ4	CAKRGSGGTGWYFDFW	IGKV1-5	IGKJ4	CQQYNSSSVTF

B2	eIIV4	78	0.632	2.04E+06	1	M37	IGHV5-51	IGHD2-2	IGHJ3	CSRLRHYGYDAFDIW	IGLV1-40	IGLJ3	CQSYDSSLGAVLF
B2	eIIV4	6352	0.488	1.57E+06	1	M38	IGHV3-23	IGHD5-12	IGHJ4	CGRGGISTTGPDYW	IGLV1-51	IGLJ2	CGTWDSSLSAGVF
B3	eIIV4	10763	1.248	6.09E+06	3	M53	IGHV3-30	IGHD3-9	IGHJ4	CAKETNPTWFFDYW	IGKV1-9	IGKJ4	CQQLNSYPVTF
B3	eIIV4	387	1.192	5.82E+06	2	M54	IGHV4-34	IGHD3-16	IGHJ4	CARRGLRLGEVSLYRGRSSVPRYYFDYW	IGLV2-14	IGLJ3	CSSYTSSSTLWVF
B5	eIIV4	20758	51.945	1.44E+09	139	M91	IGHV4-61	IGHD3-16	IGHJ4	CARFYGSVLDYW	IGKV1-12	IGKJ5	CQQGKTSPITF
B5	eIIV4	29883	8.373	2.32E+08	33	M92	IGHV2-70	IGHD4-23	IGHJ4	CTQMTPVGSGLSPYFHYW	IGKV3-20	IGKJ1	CQQFGNSFWTF
B5	eIIV4	34386	2.140	5.94E+07	7	M94	IGHV1-2	IGHD3-16	IGHJ6	CAKIILGGGGLDVW	IGLV2-11	IGLJ3	CSSYADTSNTWVF
B5	eIIV4	34038	1.571	4.36E+07	11	M96	IGHV4-31	IGHD7-27	IGHJ4	CASSLPSANWGNTGALRGW	IGKV1-5	IGKJ1	CQQYNGYPWTF
B5	eIIV4	1	0.948	2.63E+07	6	M97	IGHV4-30	IGHD1-7	IGHJ6	CARGEVWGTMDIW	IGLV1-51	IGLJ2	CGAWDSGLSAVVF
B5	eIIV4	597	0.880	2.44E+07	5	M98	IGHV5-51	IGHD3-16	IGHJ6	CARPKRELDYGSTYLFHGMDVW	IGKV4-1	IGKJ1	CQQYYFTPWTF
C1	ccIIV4	19670	17.516	4.46E+08	36	UT14	IGHV3-30	IGHD5-24	IGHJ4	CAKERDRDGYNEGIYDYW	IGKV3-11	IGKJ5	CQQRYNWPITF
C2	ccIIV4	1555	4.801	2.00E+08	16	M69	IGHV1-69	IGHD5-12	IGHJ6	CARDWELKPEHGSGWHDYSNYCLDVW	IGKV3-20	IGKJ1	CQQYDRSPWTF
C2	ccIIV4	451	4.726	1.97E+08	12	M70	IGHV1-3	IGHD2-2	IGHJ4	CARQSWLGFCRDTSCYPGDFW	IGKV3-20	IGKJ5	CQQYGSSPITF
C2	ccIIV4	20802	3.021	1.26E+08	13	M71	IGHV4-61	IGHD6-6	IGHJ4	CARDVIPRPGFDYW	IGLV10-54	IGLJ2	CSAWDSSLSAVVI
C3	ccIIV4	717	8.791	7.06E+07	14	M101	IGHV1-3	IGHD3-9	IGHJ5	CARDSQSRYFDWFSRRPSWFDPW	IGLV6-57	IGLJ2	CQSSDNDNVFF
C4	ccIIV4	9092	38.056	2.73E+08	7	M105	IGHV1-46	IGHD3-22	IGHJ6	CARDLSPHYDSSGYKYFANYLYGMDVW	IGLV1-44	IGLJ1	CGTWDDSLNGYVF
C4	ccIIV4	8234	6.596	4.74E+07	19	M106	IGHV4-38	IGHD2-15	IGHJ4	CARATTTRYFDSW	IGKV1-5	IGKJ1	CQQYTSPPWTF
C4	ccIIV4	19125	0.963	6.91E+06	5	M107	IGHV4-39	IGHD6-25	IGHJ4	CARHRSAADLDYW	IGLV8-61	IGLJ3	CLLHMGSGIWVF
C4	ccIIV4	23763	0.804	5.77E+06	7	M109	IGHV1-18	IGHD4-23	IGHJ4	CVRGNGVTTGGLDYW	IGLV7-46	IGLJ2	CLLFDSDGRGVF
C4	ccIIV4	19940	0.502	3.60E+06	5	M110	IGHV3-15	IGHD4-11	IGHJ4	CIASPDWSHSW	IGKV1-5	IGKJ4	CQNYDGCF
C5	ccIIV4	63	10.451	6.49E+07	13	M111	IGHV4-59	IGHD3-3	IGHJ5	CARGGQFNFWSAYRSGEGFEPW	IGKV2-30	IGKJ1	CMQGTYWPPTF
C5	ccIIV4	28419	4.505	2.80E+07	7	M112	IGHV5-51	IGHD3-3	IGHJ6	CATSSPHHDFWSGSLDGPDYYYAMAVW	IGLV1-40	IGLJ3	CQSYDSSLSGSYWVF
C5	ccIIV4	1419	3.232	2.01E+07	8	M114	IGHV3-48	IGHD6-6	IGHJ6	CARDVASTSDWYRSSRLTYAMDVW	IGLV3-19	IGLJ3	CNSRDSTGNHGTQMF
C5	ccIIV4	17893	2.575	1.60E+07	8	M115	IGHV1-2	IGHD4-11	IGHJ1	CARGQDYTYENL	IGLV2-8	IGLJ2	CSSYAGNVDLVF

Ig-Seq, BCR-Seq, and biochemical Information of recombinant mAbs representing dominant clonotypes identified in the serological repertoire on day 28 following vaccination.

Supplemental Table 3. Antigens used in multiplexed Luminex MIADA assay.

Abbreviation	Virus strain	Type (subtype or lineage)	Egg or Cell-origin	Ecto/GH HA1	GISAID Accession No.	Resource
H1.SC.18	A/South Carolina/1/18	A(H1N1)	lung biopsy	Ecto	EPI5571	CDC
H1.PR.34	A/Puerto Rico/8/34	A(H1N1)	Unknown	Ecto	EPI252235	CDC
H1.MA.43	A/Marton/43	A(H1N1)	Egg	Ecto	EPI240837	CDC
H1.USS.77	A/USSR/90/77	A(H1N1)	Egg	Ecto	EPI390455	CDC
H1.TW.86	A/Taiwan/01/86	A(H1N1)	Cell	Ecto	EPI318034	CDC
H1.NC.99	A/New Caledonia/20/99	A(H1N1)	Unknown	Ecto	EPI18473	CDC
H1.CA.09	A/California/7/2009	A(H1N1)	Cell	Ecto	EPI177294	IRR
H1.MI.15	A/Michigan/45/2015	A(H1N1)	Egg	Ecto	EPI685579	CDC
H3.HK.68	A/Hong Kong/8/68	A(H3N2)	Unknown	Ecto	EPI240947	CDC
H3.Vic.75	A/Victoria/3/75	A(H3N2)	Unknown	Ecto	EPI131278	CDC
H3.BNK.79	A/Bangkok/1/79	A(H3N2)	Egg	Ecto	EPI377537	CDC
H3.LNG.86	A/Leningrad/360/86	A(H3N2)	Egg	Ecto	EPI385984	CDC
H3.SH.87	A/Shanghai/11/87	A(H3N2)	Egg	Ecto	EPI390002	CDC
H3.BJ.92	A/Beijing/32/92	A(H3N2)	Cell	Ecto	EPI365898	CDC
H3.JHN.94	A/Johannesburg/33/94	A(H3N2)	Egg	Ecto	EPI390018	CDC
H3.NC.95	A/Nanchang/933/95	A(H3N2)	Cell	Ecto	EPI362794	CDC
H3.Syd.97	A/Sydney/5/97	A(H3N2)	Cell	Ecto	EPI362863	CDC
H3.Pan.99	A/Panama/2007/99	A(H3N2)	Unknown	Ecto	EPI105036	CDC
H3.WY.03	A/Wyoming/03/2003	A(H3N2)	Cell	Ecto	EPI152388	CDC
H3.CA.04	A/California/7/2004	A(H3N2)	Cell	Ecto	EPI367105	CDC
H3.WI.05	A/Wisconsin/67/2005	A(H3N2)	Cell	GH HA1	EPI106424	CDC
H3.Per.09	A/Perth/16/2009	A(H3N2)	Cell	GH HA1	EPI182941	IRR
H3.TX.12	A/Texas/50/2012	A(H3N2)	Cell	GH HA1	EPI398417	CDC
H3.MD.14	A/Maryland/26/2014	A(H3N2)	Cell	GH HA1	EPI550983	CDC
H3.Sin.16	A/Singapore/INFIMH-16–0019/2016	A(H3N2)	Cell	GH HA1	EPI780183	CDC
B-V.BR.08	B/Brisbane/60/2008	B(Victoria)	Egg	GH HA1	EPI173277	IRR
B-V.Co.17	B/Colorado/06/2017	B(Victoria)	Cell	GH HA1	EPI1056637	CDC
B-Y.Phu.13	B/Phuket/3073/2013	B(Yamagata)	Cell	GH HA1	EPI363743	CDC
A/NP	A/Brisbance/10/2007	A(H3N2)	Cell	N/A	EPI353307	CDC
H2.Jap.57	A/Japan/305/57	A(H2N2)	Unknown	Ecto	EPI128485	IRR
H5.Ind.05	A/Indonesia/5/2005	A(H5N1)	Egg	Ecto	EPI376537	IRR
H7.SH.13	A/Shanghai/2/2013	A(H7N9)	Egg	Ecto	EPI439502	CDC
PA	Protein A (control)	N/A	N/A	N/A	N/A	Fisher Scientific

Information of influenza antigens used in multiplexed Luminex MIADA assay. Serum or recombinant mAbs were used to measure the binding breadth against a panel of antigens. N/A, non-applicable; Ecto, ectodomain; GH HA1, globular head region (HA1) of HA.

Supplemental Table 4. Complete serum binding responses and breadth determined by multiplexed Luminex assay.

Luminex MFI	RIV4			eIIV4			ccIIV4		
**H3 strain Abbreviation**	**day 0**	**day 28**	**Fold Change**	**day 0**	**day 28**	**Fold Change**	**day 0**	**day 28**	**Fold Change**
H3.HK.68	1382 [470 – 4041]	2120 [538 – 4779]	1.4 [1.1 – 1.6]	685 [173 – 1454]	610 [269 – 1435]	1.1 [0.9 – 1.6]	712 [442 – 2874]	1257 [682 – 2653]	1.2 [0.9 – 3.2]
H3.Vic.75	1341 [865 – 2475]	2259 [1396 – 3261]	1.6 [1.2 – 1.9]	781 [659 – 2013]	1053 [966 – 2046]	1.3 [0.9 – 1.6]	1113 [623 – 1444]	1405 [1060 – 2341]	1.2 [1 – 2.5]
H3.BNK.79	813 [539 – 2037]	1053 [1002 – 4666]	1.7 [1.1 – 2.3]	1322 [509 – 2428]	1366 [585 – 2128]	1.1 [0.9 – 1.5]	755 [386 – 7382]	1007 [567 – 3231]	1.1 [0.4 – 2]
H3.LNG.86	659 [481 – 4071]	1145 [877 – 5600]	1.4 [1.1 – 2.4]	1703 [947 – 4879]	1664 [1110 – 4371]	1.2 [0.9 – 1.3]	1054 [360 – 6031]	1178 [645 – 4585]	1.2 [0.7 – 1.8]
H3.SH.87	567 [295 – 1554]	956 [807 – 1491]	1.4 [1 – 4]	801 [441 – 2287]	1085 [594 – 1986]	1.2 [0.9 – 1.5]	842 [266 – 1833]	980 [540 – 2128]	1.2 [0.9 – 2.2]
H3.BJ.92	880 [301 – 1421]	1273 [1066 – 1896]	1.3 [1.1 – 5.1]	916 [417 – 2410]	1135 [577 – 2552]	1.2 [0.9 – 1.5]	872 [466 – 1210]	1110 [453 – 2174]	1.6 [1 – 2.3]
H3.JHN.94	445 [102 – 1511]	966 [637 – 1880]	1.4 [1 – 9.5]	631 [144 – 2650]	581 [280 – 2604]	1.2 [0.9 – 1.9]	520 [95 – 657]	858 [107 – 1381]	1.7 [1.1 – 2.3]
H3.NC.95	129 [115 – 331]	300 [139 – 686]	1.5 [1.1 – 6]	257 [119 – 853]	352 [150 – 896]	1.2 [0.9 – 1.5]	247 [133 – 364]	453 [135 – 707]	1.2 [1 – 2.9]
H3.Syd.97	523 [284 – 1338]	1018 [911 – 1777]	1.7 [1.3 – 3.6]	724 [237 – 2472]	807 [400 – 2580]	1.2 [0.9 – 1.7]	646 [265 – 1177]	1211 [278 – 2433]	1.9 [1 – 3.2]
H3.Pan.99	519 [119 – 1926]	1728 [1074 – 2438]	2.1 [1.3 – 14.5]	877 [225 – 4347]	1078 [586 – 4338]	1.2 [0.9 – 2.6]	804 [180 – 1437]	1987 [198 – 4420]	2 [1.1 – 5.5]
H3.WY.03	450 [144 – 979]	1379 [810 – 2677]	2.3 [1.4 – 18.6]	739 [178 – 4221]	766 [375 – 4148]	1.2 [0.9 – 2.1]	396 [333 – 951]	1227 [393 – 3834]	2 [1 – 4]
H3.CA.04	355 [260 – 1281]	2753 [874 – 4772]	2.5 [2.1 – 16.2]	670 [181 – 1460]	1129 [411 – 1575]	1.6 [1 – 2.3]	537 [235 – 1030]	944 [576 – 2222]	2.2 [0.9 – 4.1]
H3.WI.05	512 [325 – 1241]	2971 [2449 – 5866]	4.8 [2.4 – 18.1]	740 [567 – 1587]	1314 [668 – 2472]	1.6 [1 – 2.3]	737 [323 – 1899]	1938 [1279 – 2515]	2.4 [0.9 – 6.5]
H3.Per.09	1455 [739 – 1667]	6715 [2294 – 13374]	4.7 [3.1 – 8]	1166 [701 – 1739]	2036 [903 – 3641]	1.6 [1.2 – 3.1]	856 [577 – 2629]	1794 [1591 – 6711]	2.1 [0.7 – 10.6]
H3.TX.12	1525 [967 – 1794]	6827 [2789 – 13619]	4.6 [2.9 – 7.6]	1130 [769 – 1753]	2279 [1003 – 3805]	1.6 [1.3 – 3.4]	1180 [630 – 2916]	2329 [1701 – 6773]	2 [0.6 – 10.8]
H3.MD.14	750 [358 – 787]	4073 [1266 – 7619]	5.2 [2.7 – 10]	535 [378 – 988]	738 [615 – 2915]	1.7 [1.3 – 3]	619 [253 – 2573]	1282 [644 – 4993]	2.1 [0.4 – 14.5]
H3.Sin.16	866 [414 – 913]	4732 [1465 – 8801]	5.2 [2.6 – 9.6]	561 [308 – 1109]	913 [586 – 3339]	1.9 [1.3 – 3]	591 [226 – 2928]	1349 [794 – 5520]	2.1 [0.5 – 14.4]

MFI: mean fluorescence intensity measured using 1:10,000 diluted sera. N=5 subjects for each vaccine group.

Supplemental Table 5. Stereotypical sequence signatures of antibodies binding to public HA epitopes.

Stereotyped features	Egg-glycan	Membrane-proximal anchor	Central stalk	Trimer-interface	Multi-donor group1+2 broadly neutralizing (bn) - stem		
	**Class 1**	**Class 2**	**Class 3**	**Class 4**	**Class 5-1**	**Class 5-2**	**Class 5-3**
**VH**	IGHV3-7	IGHV3-23 or IGHV3-30 or IGHV3-30-3 or IGHV3-48	IGHV1-69	-	IGHV6-1	IGHV1-18	IGHV1-18
**CDRH2**	-	-	[ILVM]53, and F54	-			Y53
**CDRH3**	CDRH3 length (aa) ≤ 14	-	contains Y	D or E at the 6th aa position	[LVIM]-F-G-[LVIM] motif, and 16≤ CDRH3 length (aa) ≤ 18	R-x-x-I-L-T-G motif, and CDRH3 length (aa) =15	Q-x-x-V motif, and 17≤ CDRH3 length (aa) ≤ 21
**JH**	IGHJ4 or IGHJ5	-	-	-	-		
**Isotype**	IgM or IgG	-	-	-	-		
**VL**	IGLV1-44 or IGLV1-51	IGKV3-11 or IGKV3-15	-	IGKV1-39	-		
**FRL2**	-	-	-	N34, [YF]36, and Y49	-		
**CDRL2**	-	-	-	[AV]50	-		
**FRL3**	-	-	-	N53, and Q55	-		
**CDRL3**	-	NWP (or NWPP) motif, and CDRK3 length (aa) = 10	-	Q at the 1st aa position	-		
**JL**	IGLJ1 or IGLJ2 or IGLJ3	IGKJ4 or IGKJ5	-	-	-		
**NGS source**	Paired VH:VL (Illumina Miseq, 2x300bp)	Paired VH:VL (Illumina Miseq, 2x300bp)	VH-only (Illumina Miseq, 2x300bp)	Paired VH:VL (Pacbio SMRT)	VH-only (Illumina Miseq, 2x300bp)		
**Ref.**	Jung, Jiwon et al., 2021; Guthmiller, Jenna J et al., 2021	Guthmiller, Jenna J et al., 2021	Ekiert et al.,2009; Sui et al.,2009; Ekiert et al.,2011; Wrammert et al., 2011; Dreyfus et al., 2012; Lingwood et al., 2012; Pappas et al.,2014; Avnir et al., 2014; Lang et al., 2017	McCarthy, Kevin R., et al., 2021; Watanabe et al., 2019; Zost, Seth J., et al., 2021; Bangaru & Lang et al., 2019	Joyce et al., 2016; Kallewaard et al., 2016; Chuang, G.Y., et al., 2021		

The table summarizes stereotyped sequence information of anti-HA antibodies binding to public HA epitopes, including egg glycan-targeting antibodies,[20,35] membrane-proximal anchor targeting antibodies,[24] IGHV1-69 central-stalk targeting antibodies[36,37,38,39,40,41,42,43,44] trimer-interface antibodies,[21,45,46,22] and multi-donor stem-directed broadly neutralizing antibodies capable of neutralizing group 1 and 2 HAs.[47,48,49] The source of NGS sequencing files that were used for the analysis of stereotypical BCR clonotypes is also provided. References for each class of stereotypical antibodies are shown.

Supplemental Table 6. UT14 – H3/HA Cryo-EM Statistics.

**EM data collection**	
Microscope	FEI Titan Krios
Voltage (kV)	300
Detector	Gatan K3
Magnification (nominal)	105,000
Pixel size (Å/pix)	0.83
Flux (e^-/pix/sec)	14.5
Frames per exposure	100
Exposure (e^-/Å^2)	80
Defocus range (mm)	0.91-2.55
Micrographs collected	3198
Tilt angle (º)	30
Sample	H3 Singapore + UT14 Fab
**3D reconstruction statistics**	
Particles	122,281
Symmetry	C1
Map sharpening B-factor	163
Unmasked resolution at 0.143 FSC (Å)	4.3
Masked resolution at 0.143 FSC (Å)	3.8
**Model refinement and validation statistics**	
Composition	
Amino acids	443
RMSD bonds (Å)	0.004
RMSD angles (º)	0.712
Average B-factors	
Amino acids	53.81
Ramachandran	
Favored (%)	90.02
Allowed (%)	9.74
Outliers (%)	0.23
Rotamer outliers (%)	3.45
Clash score	14.17
C-beta outliers (%)	0
CaBLAM outliers (%)	7.88
CC (mask)	0.69
MolProbity score	2.62
EMRinger score	2.04

3. References.

1 Dawood FS, Naleway AL, Flannery B, *et al.* Comparison of the Immunogenicity of Cell Culture-Based and Recombinant Quadrivalent Influenza Vaccines to Conventional Egg-Based Quadrivalent Influenza Vaccines Among Healthcare Personnel Aged 18-64 Years: A Randomized Open-Label Trial. *Clin Infect Dis Off Publ Infect Dis Soc Am* 2021; **73**: 1973–81.

2 Gross FL, Bai Y, Jefferson S, Holiday C, Levine MZ. Measuring Influenza Neutralizing Antibody Responses to A(H3N2) Viruses in Human Sera by Microneutralization Assays Using MDCK-SIAT1 Cells. *J Vis Exp JoVE* 2017; published online Nov 22. DOI:10.3791/56448.

3 Yang H, Carney P, Stevens J. Structure and Receptor binding properties of a pandemic H1N1 virus hemagglutinin. *PLOS Curr Influenza* 2010; published online March 22. DOI:10.1371/currents.RRN1152.

4 Yang H, Carney PJ, Chang JC, Villanueva JM, Stevens J. Structural Analysis of the Hemagglutinin from the Recent 2013 H7N9 Influenza Virus. *J Virol* 2013; **87**: 12433–46.

5 Yang H, Chang JC, Guo Z, *et al.* Structural Stability of Influenza A(H1N1)pdm09 Virus Hemagglutinins. *J Virol* 2014; **88**: 4828–38.

6 Li Z-N, Liu F, Gross FL, *et al.* Antibody Landscape Analysis following Influenza Vaccination and Natural Infection in Humans with a High-Throughput Multiplex Influenza Antibody Detection Assay. *mBio* 2021; **12**: e02808-20.

7 Li Z-N, Trost JF, Weber KM, *et al.* Novel multiplex assay platforms to detect influenza A hemagglutinin subtype-specific antibody responses for high-throughput and in-field applications. *Influenza Other Respir Viruses* 2017; **11**: 289–97.

8 Li ZN, Liu F, Jefferson S, *et al.* Multiplex Detection of Antibody Landscapes to Severe Acute Respiratory Syndrome Coronavirus 2 (SARS-CoV-2)/Influenza/Common Human Coronaviruses Following Vaccination or Infection With SARS-CoV-2 and Influenza. *Clin Infect Dis* 2022; **75**: S271–84.

9 Lee J, Boutz DR, Chromikova V, *et al.* Molecular-level analysis of the serum antibody repertoire in young adults before and after seasonal influenza vaccination. *Nat Med* 2016; **22**: 1456–64.

10 Ippolito GC, Hoi KH, Reddy ST, *et al.* Antibody Repertoires in Humanized NOD-scid-IL2Rγnull Mice and Human B Cells Reveals Human-Like Diversification and Tolerance Checkpoints in the Mouse. *PLOS ONE* 2012; **7**: e35497.

11 McDaniel JR, DeKosky BJ, Tanno H, Ellington AD, Georgiou G. Ultra-high-throughput sequencing of the immune receptor repertoire from millions of lymphocytes. *Nat Protoc* 2016; **11**: 429–42.

12 Tanno H, McDaniel JR, Stevens CA, *et al.* A facile technology for the high-throughput sequencing of the paired VH:VL and TCRβ:TCRα repertoires. *Sci Adv* 2020; **6**: eaay9093.

13 Ellefson JW, Gollihar J, Shroff R, Shivram H, Iyer VR, Ellington AD. Synthetic evolutionary origin of a proofreading reverse transcriptase. *Science* 2016; **352**: 1590–3.

14 Zhang J, Kobert K, Flouri T, Stamatakis A. PEAR: a fast and accurate Illumina Paired-End reAd mergeR. *Bioinforma Oxf Engl* 2014; **30**: 614–20.

15 Bolotin DA, Poslavsky S, Mitrophanov I, *et al.* MiXCR: software for comprehensive adaptive immunity profiling. *Nat Methods* 2015; **12**: 380–1.

16 Bolger AM, Lohse M, Usadel B. Trimmomatic: a flexible trimmer for Illumina sequence data. *Bioinforma Oxf Engl* 2014; **30**: 2114–20.

17 Edgar RC. Search and clustering orders of magnitude faster than BLAST. *Bioinforma Oxf Engl* 2010; **26**: 2460–1.

18 DeKosky BJ, Kojima T, Rodin A, *et al.* In-depth determination and analysis of the human paired heavy- and light-chain antibody repertoire. *Nat Med* 2015; **21**: 86–91.

19 Lavinder JJ, Wine Y, Giesecke C, *et al.* Identification and characterization of the constituent human serum antibodies elicited by vaccination. *Proc Natl Acad Sci U S A* 2014; **111**: 2259–64.

20 Jung J, Mundle ST, Ustyugova IV, *et al.* Influenza vaccination in the elderly boosts antibodies against conserved viral proteins and egg-produced glycans. *J Clin Invest* 2021; **131**: 148763.

21 McCarthy KR, Lee J, Watanabe A, *et al.* A Prevalent Focused Human Antibody Response to the Influenza Virus Hemagglutinin Head Interface. *mBio* 2021; **12**: e0114421.

22 Bangaru S, Lang S, Schotsaert M, *et al.* A Site of Vulnerability on the Influenza Virus Hemagglutinin Head Domain Trimer Interface. *Cell* 2019; **177**: 1136-1152.e18.

23 Watanabe A, McCarthy KR, Kuraoka M, *et al.* Antibodies to a Conserved Influenza Head Interface Epitope Protect by an IgG Subtype-Dependent Mechanism. *Cell* 2019; **177**: 1124-1135.e16.

24 Guthmiller JJ, Han J, Utset HA, *et al.* Broadly neutralizing antibodies target a haemagglutinin anchor epitope. *Nature* 2022; **602**: 314–20.

25 Corti D, Voss J, Gamblin SJ, *et al.* A neutralizing antibody selected from plasma cells that binds to group 1 and group 2 influenza A hemagglutinins. *Science* 2011; **333**: 850–6.

26 Dreyfus C, Laursen NS, Kwaks T, *et al.* Highly Conserved Protective Epitopes on Influenza B Viruses. *Science* 2012; **337**: 1343–8.

27 Zheng SQ, Palovcak E, Armache J-P, Cheng Y, Agard DA. Anisotropic Correction of Beam-induced Motion for Improved Single-particle Electron Cryo-microscopy. 2016; : 061960.

28 Punjani A, Rubinstein JL, Fleet DJ, Brubaker MA. cryoSPARC: algorithms for rapid unsupervised cryo-EM structure determination. *Nat Methods* 2017; **14**: 290–6.

29 Jumper J, Evans R, Pritzel A, *et al.* Highly accurate protein structure prediction with AlphaFold. *Nature* 2021; **596**: 583–9.

30 Abanades B, Wong WK, Boyles F, Georges G, Bujotzek A, Deane CM. ImmuneBuilder: Deep-Learning models for predicting the structures of immune proteins. *Commun Biol* 2023; **6**: 1–8.

31 Pettersen EF, Goddard TD, Huang CC, *et al.* UCSF ChimeraX: Structure visualization for researchers, educators, and developers. *Protein Sci* 2021; **30**: 70–82.

32 Emsley P, Cowtan K. Coot: model-building tools for molecular graphics. *Acta Crystallogr D Biol Crystallogr* 2004; **60**: 2126–32.

33 Adams PD, Afonine PV, Bunkóczi G, *et al.* PHENIX: a comprehensive Python-based system for macromolecular structure solution. *Acta Crystallogr D Biol Crystallogr* 2010; **66**: 213–21.

34 Croll TI. ISOLDE: a physically realistic environment for model building into low-resolution electron-density maps. *Acta Crystallogr Sect Struct Biol* 2018; **74**: 519–30.

35 Guthmiller JJ, Utset HA, Henry C, *et al.* An Egg-Derived Sulfated N-Acetyllactosamine Glycan Is an Antigenic Decoy of Influenza Virus Vaccines. *mBio* 2021; **12**: 10.1128/mbio.00838-21.

36 Ekiert DC, Bhabha G, Elsliger M-A, *et al.* Antibody Recognition of a Highly Conserved Influenza Virus Epitope. *Science* 2009; **324**: 246–51.

37 Sui J, Hwang WC, Perez S, *et al.* Structural and functional bases for broad-spectrum neutralization of avian and human influenza A viruses. *Nat Struct Mol Biol* 2009; **16**: 265–73.

38 Ekiert DC, Friesen RHE, Bhabha G, *et al.* A Highly Conserved Neutralizing Epitope on Group 2 Influenza A Viruses. *Science* 2011; **333**: 843–50.

39 Wrammert J, Koutsonanos D, Li G-M, *et al.* Broadly cross-reactive antibodies dominate the human B cell response against 2009 pandemic H1N1 influenza virus infection. *J Exp Med* 2011; **208**: 181–93.

40 Dreyfus C, Laursen NS, Kwaks T, *et al.* Highly Conserved Protective Epitopes on Influenza B Viruses. *Science* 2012; **337**: 1343–8.

41 Lingwood D, McTamney PM, Yassine HM, *et al.* Structural and genetic basis for development of broadly neutralizing influenza antibodies. *Nature* 2012; **489**: 566–70.

42 Pappas L, Foglierini M, Piccoli L, *et al.* Rapid development of broadly influenza neutralizing antibodies through redundant mutations. *Nature* 2014; **516**: 418–22.

43 Avnir Y, Tallarico AS, Zhu Q, *et al.* Molecular Signatures of Hemagglutinin Stem-Directed Heterosubtypic Human Neutralizing Antibodies against Influenza A Viruses. *PLOS Pathog* 2014; **10**: e1004103.

44 Lang S, Xie J, Zhu X, Wu NC, Lerner RA, Wilson IA. Antibody 27F3 Broadly Targets Influenza A Group 1 and 2 Hemagglutinins through a Further Variation in VH1-69 Antibody Orientation on the HA Stem. *Cell Rep* 2017; **20**: 2935–43.

45 Watanabe A, McCarthy KR, Kuraoka M, *et al.* Antibodies to a Conserved Influenza Head-interface Epitope Protect by an IgG Subtype Dependent Mechanism. *Cell* 2019; **177**: 1124-1135.e16.

46 Zost SJ, Dong J, Gilchuk IM, *et al.* Canonical features of human antibodies recognizing the influenza hemagglutinin trimer interface. *J Clin Invest*; **131**: e146791.

47 Joyce MG, Wheatley AK, Thomas PV, *et al.* Vaccine-Induced Antibodies that Neutralize Group 1 and 2 Influenza A Viruses. *Cell* 2016; **166**: 609–23.

48 Kallewaard NL, Corti D, Collins PJ, *et al.* Structure and Function Analysis of an Antibody Recognizing All Influenza A Subtypes. *Cell* 2016; **166**: 596–608.

49 Chuang G-Y, Shen C-H, Cheung CS-F, *et al.* Sequence-Signature Optimization Enables Improved Identification of Human HV6-1-Derived Class Antibodies That Neutralize Diverse Influenza A Viruses. *Front Immunol* 2021; **12**. https://www.frontiersin.org/articles/10.3389/fimmu.2021.662909 (accessed June 28, 2023).
